# Advances in Structural Colors-Mechanisms, Quantitative Evaluation, and Applications: A Review

**DOI:** 10.3390/nano16161031

**Published:** 2026-08-19

**Authors:** Chung-Yu Yu, Chin-An Ku, Chen-Kuei Chung

**Affiliations:** Department of Mechanical Engineering, National Cheng Kung University, Tainan 701, Taiwan

**Keywords:** structural colors, iridescent, non-iridescent, nanomaterials, nanofilms, photonic crystals, quantification, sensing, anti-counterfeiting, spectroscopy, tunable color

## Abstract

Structural colors, generated by the physical interaction of light with micro- and nanostructured architectures, have emerged as an important platform in nanophotonics owing to their high color saturation, exceptional photostability, and long-term color durability. This review provides a comprehensive overview of recent advances in structural colors and establishes a unified classification framework based on their macroscopic angular optical responses. The intrinsic angular characteristics of four fundamental color-generation mechanisms are first distinguished, providing the physical basis for classifying structural colors into iridescent and non-iridescent systems. Representative iridescent architectures, including thin films, one-dimensional (1D) to three-dimensional (3D) photonic crystals, and diffraction gratings, are systematically reviewed, together with non-iridescent strategies based on independent plasmonic and dielectric resonators, quasi-amorphous structures, and engineered metasurfaces. Strategies for enhancing structural color visibility and saturation through absorption management are further discussed, particularly for suppressing undesired broadband and multiple-scattering backgrounds. Additionally, this review systematically summarizes quantitative methodologies for evaluating structural colors, including spectral metrics, CIE 1931 and CIE1976 color spaces, CIEDE2000 color difference, quantitative angular-response metrics, spatial resolution and pixel limits, and structural-order characterization using orientation parameters and two-dimensional fast Fourier transform (2D FFT) analysis. Particular attention is given to the quantitative assessment of angular stability through wavelength shifts and perceptual color differences, while recognizing that a universally accepted numerical boundary between iridescent and non-iridescent coloration has not yet been established. Representative functional applications are also reviewed, including self-cleaning coatings, passive daytime radiative cooling, label-free chemical and gas sensing, reflectometric interference spectroscopy (RIfS), surface-enhanced Raman scattering (SERS), and anti-counterfeiting. By integrating color-generation mechanisms, angular optical responses, quantitative evaluation methods, and functional applications, this review provides a unified framework for objectively comparing structural color platforms and highlights key trade-offs among color quality, angular stability, structural precision, durability, scalability, and multifunctionality, thereby providing design guidance for next-generation optical materials and devices.

## 1. Introduction: Overview of Structural Color and Its Advances

Structural color is a fascinating phenomenon in which color is generated solely through the interaction of light with microscopic structures. Structural colors arise from optical mechanisms such as interference, diffraction, scattering, and resonance, which are produced by micro- and nanoscale architectures [1,2,3,4,5]. Owing to their high reflectance, high color saturation, and excellent durability without fading, structural colors have attracted considerable scientific and ecological interest [6,7,8,9,10]. In recent years, advances in nanotechnology have greatly improved the feasibility of fabricating structural colors. Using micro- and nanostructured materials, researchers can precisely engineer optical architectures through two main approaches. The first is the top–down approach, in which techniques such as electron-beam lithography, photolithography, etching, and laser processing are used to fabricate highly precise periodic structures [11,12,13]. Although these methods provide excellent structural control, they are often expensive and limited to small-scale production. The second is the bottom–up approach. By using colloidal suspensions of monodisperse micro- or nanoparticles, 3D printing, or self-assembly templates [14,15,16,17,18], photonic crystals and thin-film nanostructures can be fabricated for structural color applications. Compared with top–down methods, this approach is more scalable, cost-effective, and suitable for producing large-area structural color films.

Unlike conventional pigments and dyes, which generate color through the selective absorption of specific wavelengths via electronic transitions within chemical molecules, structural colors originate from the interaction between light and micro- and nanostructured surfaces [19,20,21,22,23,24]. Because of their optical origin, structural colors exhibit several remarkable characteristics, including high color saturation [9], high brightness [25], or iridescence [26]. One of the most significant advantages of structural colors is their exceptional photostability. Since the reflected wavelength is determined by precisely designed structural parameters, vivid and pure colors can be produced without relying on chemical additives. In contrast, conventional dyes often suffer from spectral broadening and unwanted absorption, resulting in relatively dull colors that gradually fade over time. Consequently, structural colors can provide more vibrant visual effects while maintaining their chromatic quality over extended periods. Another important feature of structural colors is their high brightness. Carefully designed optical structures can selectively enhance the reflection of specific wavelengths while minimizing energy loss. As a result, structural colors generally appear brighter and more vivid than conventional dyes [27,28], which absorb a substantial portion of the incident light. This advantage makes structural colors highly attractive for applications such as optical devices, displays, sensors, and decorative materials that require strong visual contrast. In addition, structural colors can exhibit iridescence, where the observed color changes with the viewing angle or illumination direction. Owing to these unique optical properties, structural colors have attracted significant attention for applications in anti-counterfeiting technologies, optical sensing, energy-efficient displays, and advanced photonic devices [29,30,31,32].

## 2. Classification of Structural Colors: Iridescent and Non-Iridescent

**Figure 1:**
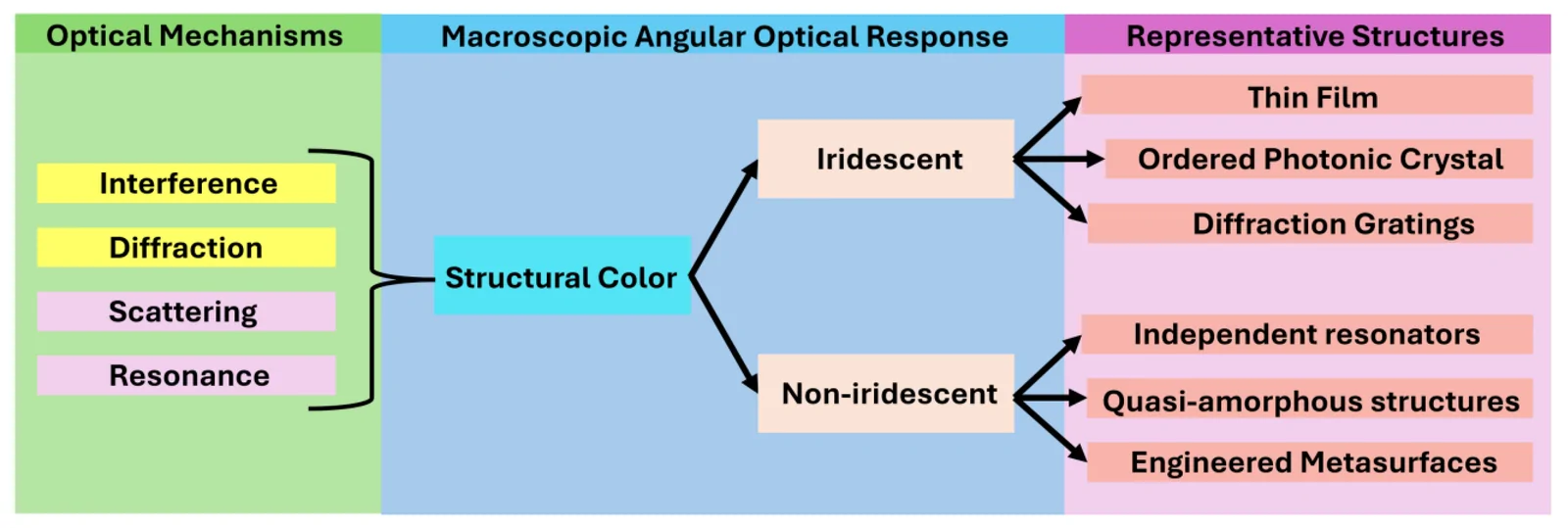
Schematic classification of representative structural color systems according to their angular optical characteristics.

Figure 1 illustrates the classification framework adopted in this review. Structural colors can be generated through four fundamental optical mechanisms: interference, diffraction, scattering, and resonance. According to their underlying physical principles, these mechanisms inherently exhibit different angular characteristics. Interference and diffraction are intrinsically angle-dependent because their wavelength-selection conditions vary with the propagation direction of light, whereas scattering and resonance are intrinsically angle-independent because their characteristic spectral responses are primarily determined by the scattering units or local resonant conditions rather than by the illumination or viewing angle. These intrinsic differences provide the physical basis for classifying structural colors according to their macroscopic angular optical responses into iridescent and non-iridescent categories. Here, iridescent structural color refers to a macroscopic optical response in which the observed color or characteristic spectrum changes appreciably with variations in the illumination-observation geometry, including the incidence angle or observation angle. In contrast, non-iridescent structural color refers to a response in which the color or characteristic spectrum remains relatively stable over a specified range of the relevant angular variables. Therefore, the term “non-iridescent” does not imply absolute invariance under all geometries, but rather sufficiently weak angular variation under the measurement conditions considered. Polarization-dependent structural coloration, in which the color changes primarily with the polarization state of the incident or detected light at a fixed illumination-observation geometry, is beyond the scope of this review and is not included in the present iridescent/non-iridescent classification. Polarization-dependent structural coloration has already been thoroughly discussed in numerous comprehensive review articles [33,34,35]. In this review, representative structures that produce iridescent structural colors include thin films, ordered PCs, and diffraction gratings, whereas representative structures that produce non-iridescent structural colors include independent resonators, quasi-amorphous structures, and engineered metasurfaces. These representative structures are discussed individually in the following sections.

It should be emphasized, however, that the optical mechanism and the macroscopic angular response are not necessarily related in a one-to-one manner. A given structure may involve one or multiple color-generation mechanisms simultaneously, and the relative contribution of each mechanism depends on material properties, structural order, characteristic dimensions, and other structural parameters. Consequently, the intrinsic angular characteristics of the underlying mechanisms may be enhanced, suppressed, or combined when implemented in an actual structure, ultimately determining its macroscopic iridescent or non-iridescent appearance.

### 2.1. Iridescent Structural Colors: Angle-Dependent Coloration

**Figure 2:**
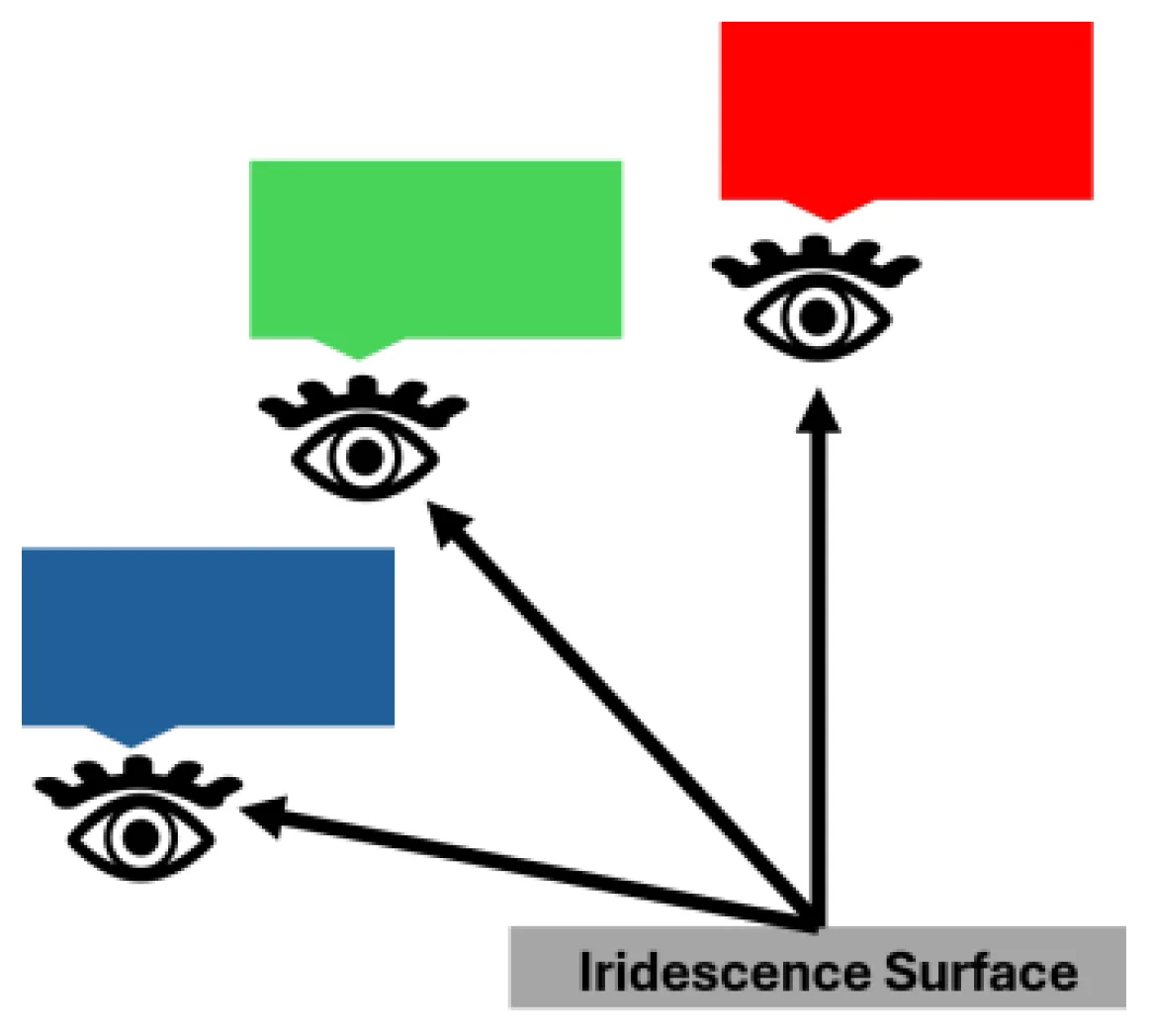
Schematic of Iridescent, which changes dynamically with variations in the incident light or viewing angle.

Iridescent structural colors, also referred to as angle-dependent structural colors, are illustrated in Figure 2 [36]. They are characterized by a macroscopic optical response in which the perceived color changes with variations in the illumination-observation geometry. Compared with conventional pigments, whose colors originate from electronic transitions within chemical molecules, iridescent structural colors provide a much higher degree of tunability through the design of optical structures [37]. Most iridescent structural colors are generated by structures possessing long-range structural order, in which the constituent structural units maintain a well-defined and continuous structural arrangement over macroscopic distances. Such structural order supports coherent collective optical responses and enables the intrinsic angle-dependent characteristics of interference and diffraction to be effectively expressed [38]. In this review, representative structures for generating iridescent structural colors include thin films, ordered photonic crystals (PCs), and diffraction gratings. These representative structures are discussed individually in the following subsections.

#### 2.1.1. Thin-Film: Single Layer or Multi-Layer

Thin-film interference is one of the most fundamental mechanisms for generating structural colors. It originates from the reflection and coherent superposition of light waves at the interfaces between materials with different refractive indices [39]. When incident light reaches an interface where the refractive index changes abruptly, part of the light is reflected back into the original medium. If the film thickness is comparable to the wavelength of light, multiple reflected beams generated at the upper and lower interfaces overlap and undergo constructive or destructive interference depending on their phase difference [39,40,41,42]. This selective enhancement or suppression of specific wavelengths is the fundamental optical mechanism by which thin-film interference produces structural colors. This section introduces thin-film interference structures, beginning with basic single-layer systems, followed by high-reflectivity Fabry–Pérot cavities, and finally multilayer structures that gradually evolve into one-dimensional photonic crystals.

**Figure 3:**
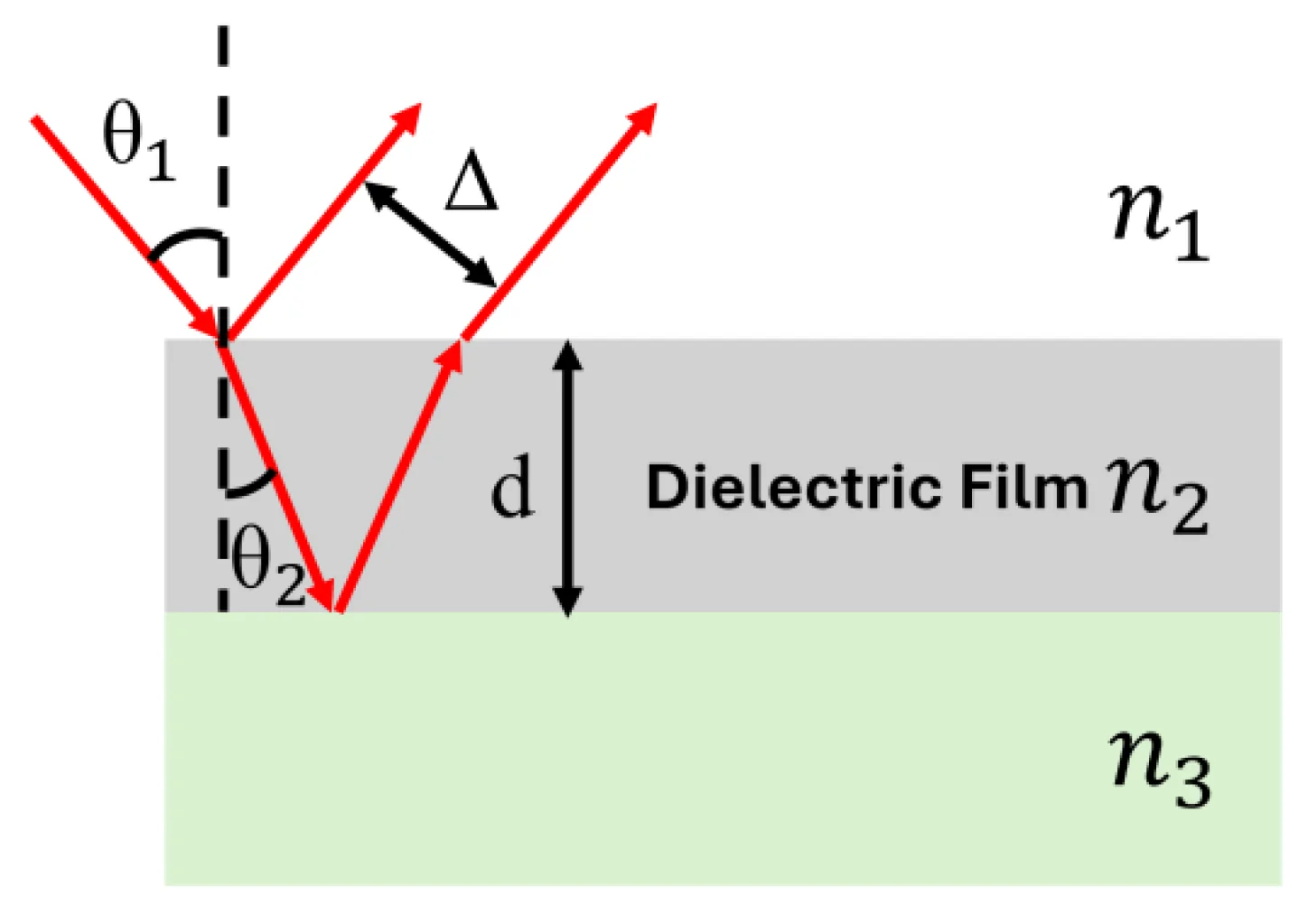
The schematic of a single-layer thin-film interference. The red arrows represent the incident/reflected light.

The simplest structure capable of generating structural color consists of a single dielectric thin film with a thickness d and a refractive index of $n_{2}$, sandwiched between a semi-infinite incident medium ($n_{1}$) and a substrate ($n_{3}$), as shown in Figure 3. When incident light reaches the upper interface at an incident angle of $\theta_{1}$, it is divided into reflected and refracted components. The refracted light propagates through the dielectric film at an angle of $\theta_{2}$, reflects from the lower interface, and then re-enters the incident medium parallel to the initially reflected beam.

The optical path difference (∆) between the two primary reflected waves is expressed as Equation (1):

(1) $$∆=2n_{2}dcos(\theta_{2})$$

To determine the conditions for constructive or destructive interference, the phase shift at the reflecting interface must first be considered. When light is reflected from a medium with a higher refractive index (a “hard reflection”), the condition for constructive interference can be expressed by Equation (2).

(2) $$∆=2n_{2}dcos\left(\theta_{2}\right)=m\lambda$$

where the *m* is the order of constructive interference (*m* = 1, 2, 3, …), and λ is the wavelength of the interference peak. For the case where $n_{1}$ < $n_{2}$ and $n_{2}$ > $n_{3}$, a phase shift occurs only at the first interface [43]. Therefore, the condition for constructive interference is expressed by Equation (3).

(3) $$∆=2n_{2}dcos\left(\theta_{2}\right)=(m+0.5)\lambda$$

By utilizing single-layer thin-film interference and controlling the dielectric film thickness (d) during fabrication, different structural colors can be readily obtained. For example, Pang et al. fabricated anodized niobium oxide films with thicknesses ranging from 81.2 to 166.6 nm on niobium foil as the dielectric layer, producing structural colors including brown, light blue, light green, earthy yellow, and pink [44]. Tang et al. used soft plasma etching to reduce the thickness of organic thin films prepared by spin-coating on glass or silicon substrates, thereby tuning the thin-film interference to generate structural colors such as light green, purple, yellow, and blue [45]. Shanker et al. employed membrane emulsification to fabricate self-assembled thin films of acetylated lignin nanoparticles. By adjusting the concentration of the nanoparticle colloid, different film thicknesses were obtained, resulting in structural colors including green, yellow, orange, and red [46].

**Figure 4:**
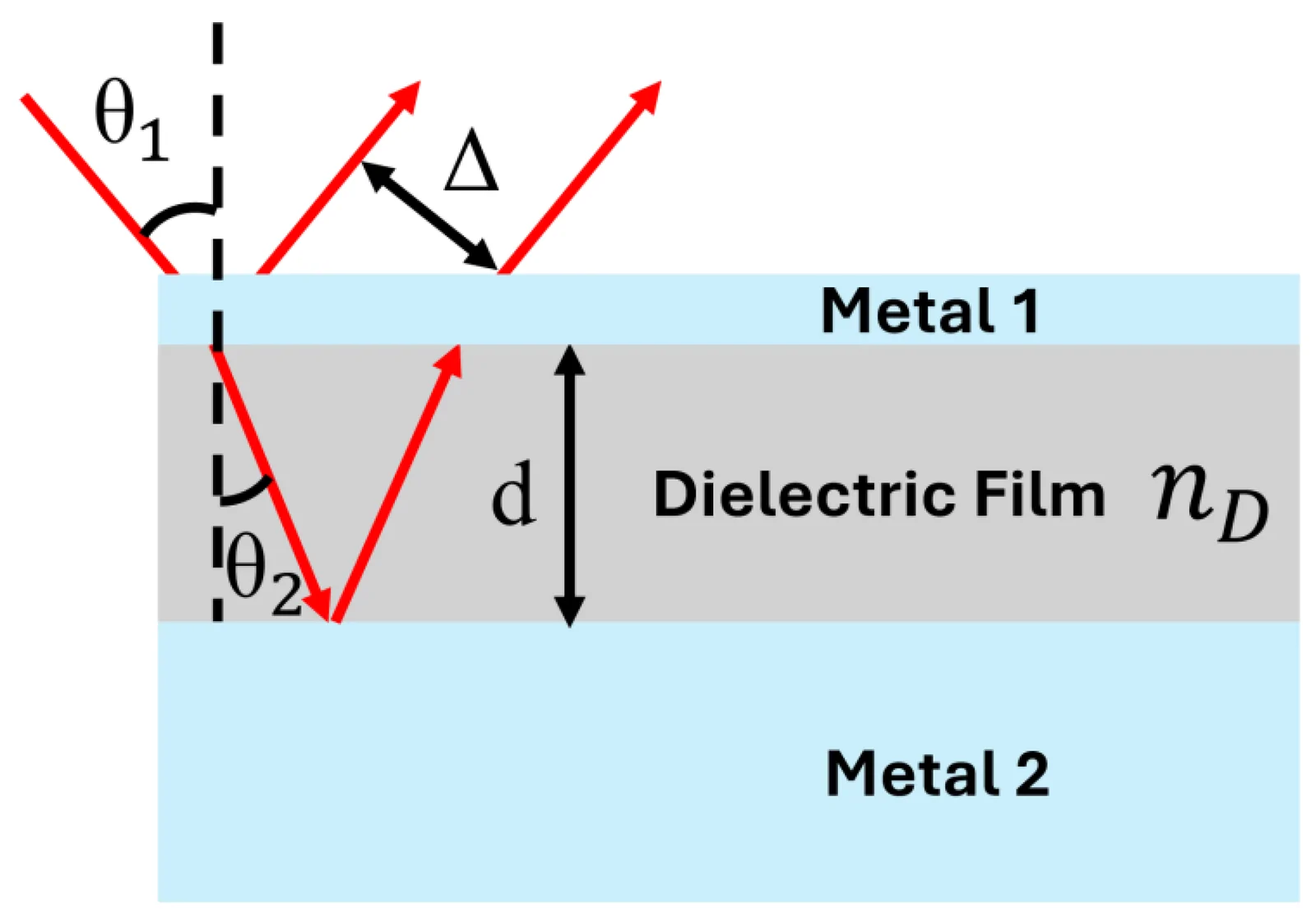
The schematic of an MDM structure. The red arrows represent the incident/reflected light.

Because the dielectric materials used as interference films (typically metal oxides or polymers) have limited reflectivity, incident light undergoes relatively weak reflection at the film interfaces. As a result, structural colors generated by single-layer thin-film interference generally exhibit low color saturation. To enhance the optical response, highly reflective layers can be coated on both sides of the dielectric film to form a Fabry-Pérot (F-P) resonant cavity. Depending on the thickness and optical properties of the cavity medium, specific wavelengths of light undergo multiple reflections within the cavity, forming standing waves that significantly enhance the saturation of the interference spectrum [2]. Because highly reflective materials in the visible wavelength range are typically metals, this configuration is commonly referred to as a metal-dielectric-metal or metal-insulator-metal (MDM or MIM) structure, as shown in Figure 4 [47,48,49,50]. Since metals absorb light, the reflected light experiences a complex phase shift at the metal interface rather than a simple phase change of 0 or π. Therefore, the interference wavelength of an MDM structure must be described by considering the phase shift, as shown in Equations (4) and (5).

(4) $$\varphi_{DM}+\varphi_{MD}+\varphi_{D}=2m\pi$$

(5) $$\varphi_{D}=\frac{2\pi n_{D}}{\lambda }2dcos(\theta_{2})$$

Here, $\varphi_{DM}$ is the phase shift at the interface between metal layer 1 and the dielectric film, $\varphi_{MD}$ is the phase shift at the interface between the dielectric film and metal layer 2, and $\varphi_{D}$ is the total phase shift accumulated within the transparent dielectric film.

**Figure 5:**
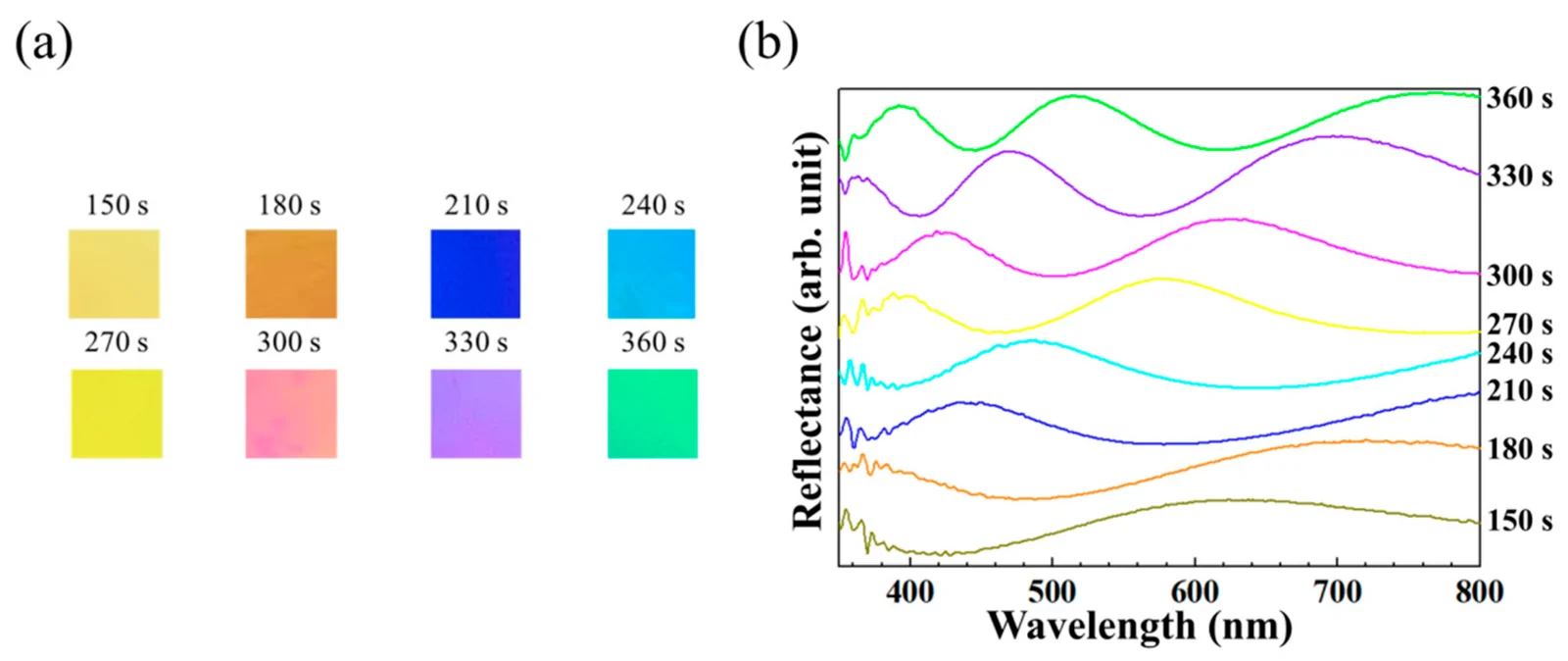
(**a**) The structure of the Al/AAO/Pt on an ITO glass with different thickness of the AAO generated by different anodization time and (**b**) The reflectance spectra of the Al/AAO/Pt on an ITO glass with different thickness of the AAO [42].

Although the phase shifts introduced by the metal layers make the interference analysis more complicated, the dielectric layer thickness and the interference wavelength still exhibit a linear relationship as long as the top and bottom metal layers remain unchanged. Therefore, the interference wavelength can be precisely tuned by controlling the thickness of the dielectric layer. For example, Yu et al. deposited an aluminum thin film on ITO glass, formed an anodic aluminum oxide (AAO) dielectric layer, and then deposited a platinum layer to fabricate an Al/AAO/Pt MDM structure [42]. By increasing the AAO thickness from 213.7 to 420.7 nm through different anodization times, the structural color was continuously tuned, producing highly saturated colors including light yellow, brown, cobalt blue, sky blue, yellow, pink, purple, and green on the glass substrate, as shown in Figure 5a,b [42].

It is worth noting that when the top metal layer of an MDM structure reaches an appropriate thickness and reflectivity, Fano resonance can occur, resulting in an asymmetric reflection spectrum [8,49,50,51,52]. This asymmetric spectral profile arises from the interference between two optical pathways: the broadband continuum produced by the direct reflection from the top metal layer and the narrowband discrete resonance leaking from multiple reflections within the dielectric cavity. The underlying mechanism is that the phase of the resonant light changes rapidly by nearly 180° as it passes through the resonance wavelength. Consequently, the resonant light undergoes constructive interference with the directly reflected light on one side of the resonance wavelength, producing a gradual increase in reflectance, while destructive interference occurs on the other side, resulting in a sharp decrease in reflectance. This interference between the two optical pathways produces the characteristic asymmetric line shape of Fano resonance, which appears as a narrow reflection valley with one gentle slope and one steep slope.

For example, Hernández-Eguía et al. deposited gold films with thicknesses of 10 and 20 nm on AAO and measured their reflection spectra [52]. When the top gold layer was 20 nm thick, a pronounced asymmetric narrow reflection valley appeared in the near-infrared region. This behavior was attributed to the interference between the strong broadband reflection from the porous gold surface and the narrowband cavity resonance generated by multiple reflections within the dielectric layer. As the porosity of the AAO increased through pore widening, the porosity of the top porous gold layer also increased, reducing the metal volume fraction and weakening the broadband reflection. As a result, the interference between the two optical pathways became weaker, causing the asymmetric Fano line shape to gradually disappear and the reflection spectrum to return to a more symmetric profile [52]. Therefore, the material and thickness of the top metal layer must be carefully designed to achieve the characteristic asymmetric spectral response associated with Fano resonance.

Multilayer films have been developed to overcome the limitations of single-layer and three-layer MDM structures. These simple structures often struggle to produce high-purity structural colors without unwanted spectral components. In addition, their optical response is highly sensitive to the incident and viewing angles, causing noticeable color changes with slight variations in viewing direction.

For example, pure red is one of the most difficult colors to achieve using single-layer interference or three-layer F-P structures. By adjusting the dielectric layer thickness, these structures typically produce orange-red or purplish-red rather than pure red. This is because the red spectral region covers a relatively broad wavelength range, making it difficult to generate a reflection peak located exclusively within the red region. When the dielectric layer is relatively thin, the red reflection band is broad and partially overlaps with the yellow region, resulting in an orange-red appearance. Increasing the dielectric layer thickness narrows the red reflection band and reduces its overlap with the yellow region. However, a thicker dielectric layer also introduces a second-order interference peak in the blue spectral region, causing the reflected color to appear purplish red [53].

**Figure 6:**
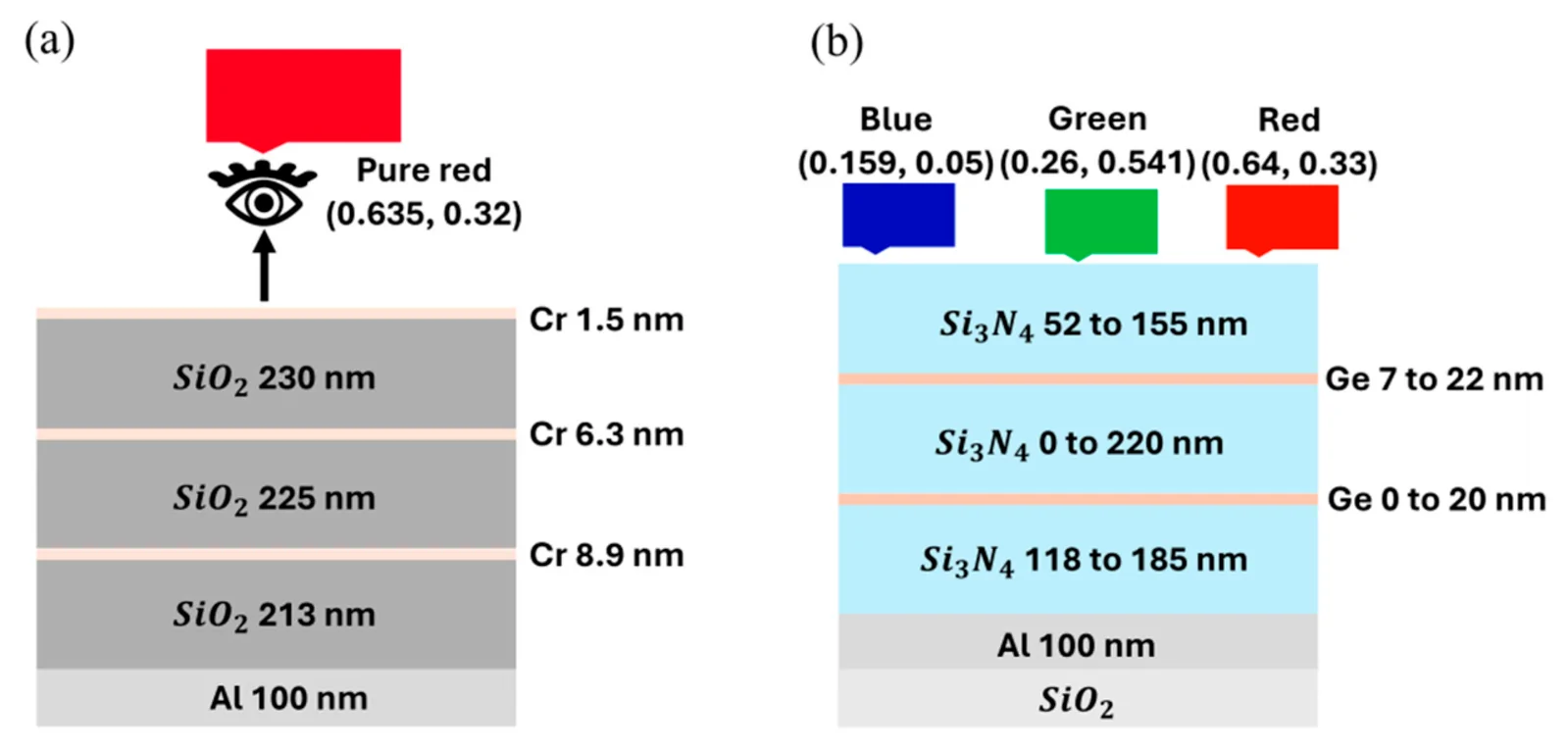
(**a**) A seven-layer F-P structure that generates a pure red color with CIE 1931 chromaticity coordinates of (0.635, 0.320). (**b**) A six-layer F-P structure that generates blue, green, and red colors with CIE 1931 chromaticity coordinates of (0.159, 0.050), (0.260, 0.541), and (0.640, 0.330), respectively.

To overcome this limitation, Chen et al. fabricated a seven-layer F-P structure consisting of three pairs of Cr/SiO_2_ layers and a bottom aluminum layer, as shown in Figure 6a. By optimizing the thickness of each interference layer, the width of the red reflection peak was reduced, producing a pure red color with CIE 1931 chromaticity coordinates of (0.635, 0.320), which is difficult to achieve using a conventional three-layer F-P structure [53]. Similarly, as shown in Figure 6b, Tao et al. fabricated a six-layer Fabry–Pérot structure using Ge and Si_3_N_4_. By adjusting the thickness of each Ge and Si_3_N_4_ layer, blue, green, and red structural colors with CIE 1931 chromaticity coordinates of (0.159, 0.050), (0.260, 0.541), and (0.640, 0.330), respectively, were obtained [54]. Owing to the high refractive index of the dielectric materials, the structure also exhibited excellent angle insensitivity, maintaining stable colors over a wide range of viewing angles [54].

In addition, it is worth noting that each layer in a multilayer interference structure generally serves a different function and therefore differs in material, thickness, or both [55,56]. In contrast, multilayer interference structures composed of periodically repeated layers with the same material combination and thickness are generally referred to as photonic crystals, emphasizing their periodic architecture and the resulting optical properties. The structural colors of photonic crystals are discussed in detail in the following section.

#### 2.1.2. Photonic Crystals: 1 to 3 Dimensional Photonic Crystals for Iridescence

Photonic crystals (PCs) are formed by the periodic modulation of the dielectric constant or refractive index within a material. They are typically composed of two or more materials with different refractive indices arranged in a periodic structure. Depending on the dimensionality of the periodicity, PCs are classified as one-dimensional (1D), two-dimensional (2D), or three-dimensional (3D) structures.

The two fundamental properties of PCs, the photonic bandgap (PBG) and photon localization, were proposed by Yablonovitch [57] and John [58], respectively, and provide the theoretical foundation for modern photonic crystal devices. The PBG originates from energy splitting at the Bragg symmetry boundary [59]. When incident and backward-reflected waves interfere within a periodic structure, standing waves are formed. The spatial distribution of these standing waves relative to the dielectric structure determines the corresponding photonic energy states [60]. According to the variational principle of electromagnetism, the electric field tends to concentrate in regions with a high dielectric constant to minimize the total electromagnetic energy. Therefore, when the antinodes of the standing wave overlap with the high-refractive-index material, the system exhibits a lower photonic energy state. In contrast, when the antinodes are located in the low-refractive-index regions, the effective refractive index experienced by the wave decreases, resulting in a higher photonic energy state. The energy splitting between these two field distributions forms the PBG in the photonic dispersion relation, preventing light within a specific wavelength range from propagating through the structure and leading to strong reflection [61]. Consequently, light with wavelengths within the PBG is reflected, producing structural color [61].

Unlike the PBG generated by a perfectly periodic structure, photon localization is achieved by intentionally introducing structural defects that break the lattice symmetry. When defects, such as point defects that modify a single lattice site or line defects that remove an entire row of lattice sites, are introduced into a PC, localized defect states are created within the original PBG. These defect states confine photons of specific frequencies within a wavelength-scale region, producing strong electromagnetic field enhancement [62]. As a result, the reflection or transmission of selected wavelengths can be controlled to generate structural colors [62,63]. It is worth noting that 1D, 2D, and 3D PCs can all produce either iridescent or non-iridescent structural colors through appropriate material selection and structural design. This section focuses on PC structures that generate iridescent structural colors.

**Figure 7:**
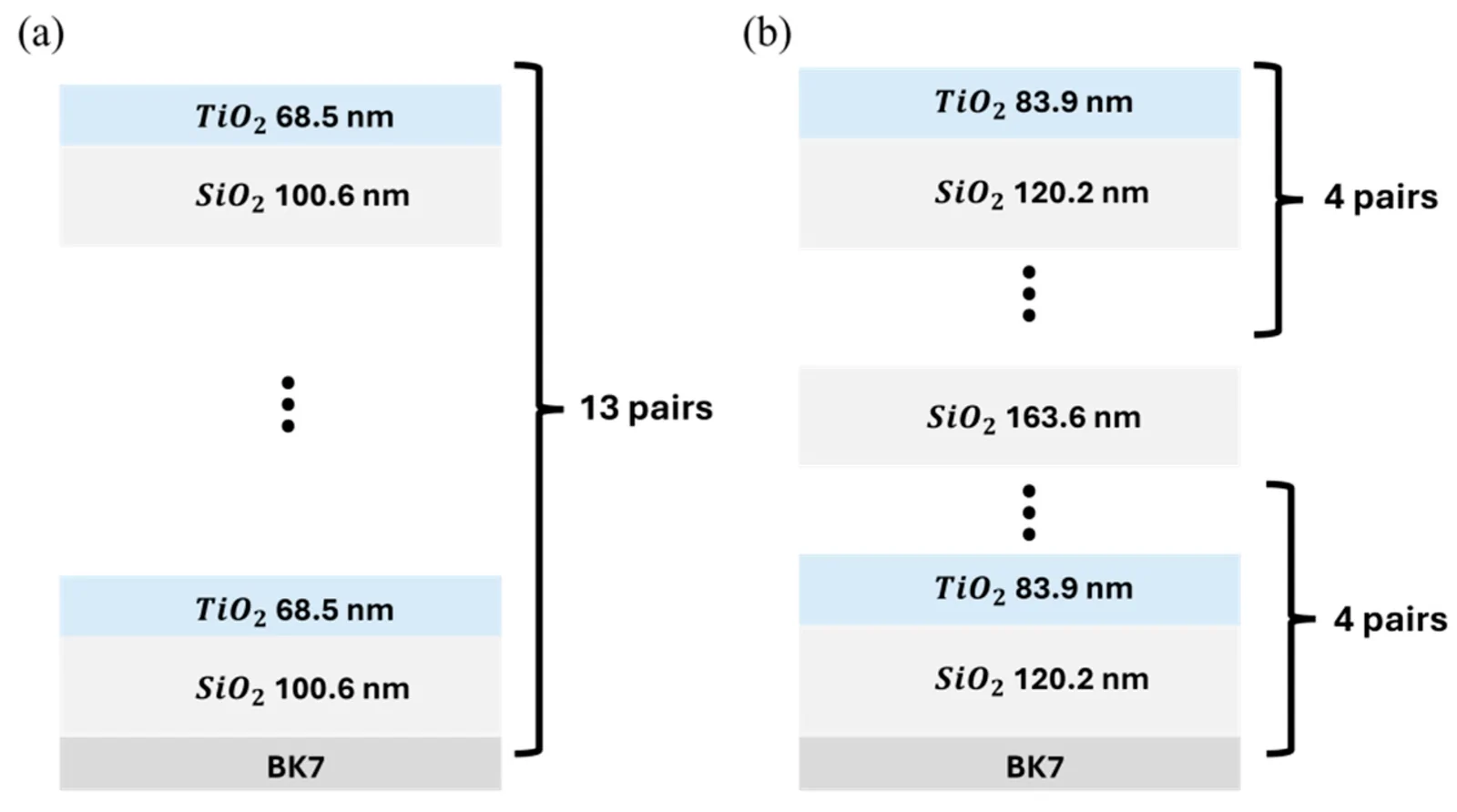
Schematic of TiO_2_/SiO_2_ PCs designed for (**a**) bandgap reflection and (**b**) defect-mode transmission [63].

As described in the previous section, a 1D PC is essentially a multilayer film composed of periodically repeated dielectric layers, and this simple structure has been widely used to generate structural colors. For example, Li et al. fabricated a 1D PC by electron-beam thermal evaporation using 13 pairs of TiO_2_ (68.5 nm) and SiO_2_ (100.6 nm), as shown in Figure 7a, producing a PBG centered in the yellow wavelength region [63]. In addition, they fabricated another structure consisting of four pairs of TiO_2_ (83.9 nm) and SiO_2_ (120.2 nm) on both the top and bottom sides, with a 163.6 nm SiO_2_ defect layer inserted at the center, as shown in Figure 7b [63]. The periodic layers generated the PBG, while the central defect layer introduced photon localization. As a result, selected wavelengths were transmitted through the structure, causing the reflected structural color on the front side of the glass to differ completely from the transmitted structural color on the back side, thereby achieving bidirectional structural colors [63].

Pan et al. developed a 1D borophene PC with a multilayer borophene/SiO_2_ structure. By combining the tunable carrier density of borophene with the thickness of the SiO_2_ defect layer, the transmission wavelength could be controlled, enabling an ultra-wide color gamut of up to 269.2% of the sRGB color space [64]. Mancarella et al. fabricated a 1D PC using pulsed laser deposition by adjusting the oxygen partial pressure to alternately grow two Ta:TiO_2_ layers with distinct microstructures and electrical properties in a single deposition process [65]. Furthermore, Li et al. fabricated a 1D PC with multiple reflection bands in the Vis-NIR region by alternately applying high and low anodization voltages to produce anodic aluminum oxide layers with different porosities [66].

The fabrication of 1D PCs is highly compatible with a wide range of thin-film deposition techniques, allowing many material systems to be used. By selecting materials with a high refractive index contrast, structural colors with high color saturation and strong reflectivity can be achieved. However, the fabrication of 1D PCs generally requires layer-by-layer deposition of multiple thin films, resulting in long fabrication times. In addition, precisely controlling the thickness and uniformity of each layer remains a significant challenge.

2D PCs are structures in which the dielectric constant or refractive index is periodically modulated within a two-dimensional plane at the wavelength scale, while no periodicity exists along the direction perpendicular to the plane. When used for structural color generation, the in-plane periodic lattice modulates the momentum and phase of incident light, producing wavelength-selective reflection.

This section focuses on iridescent structural colors generated by 2D PCs, particularly monolayer colloidal self-assembled films such as polystyrene (PS) microsphere arrays. These structures mainly function as 2D Bragg diffraction gratings. Because their reflected wavelength strongly depends on the optical path difference and the angle of incidence, they exhibit pronounced angle-dependent color changes, which are the characteristic feature of iridescent structural colors.

In contrast, high-precision dielectric metasurfaces fabricated using advanced micro- and nanofabrication techniques, such as high-refractive-index silicon or titanium dioxide nanopillar arrays, also possess two-dimensional periodicity at the subwavelength scale. However, their color-generation mechanism no longer relies on Bragg diffraction. Instead, it is dominated by localized Mie resonances within individual nanostructures. This strong near-field electromagnetic confinement effectively suppresses the spectral blueshift caused by changes in the viewing angle, allowing the metasurfaces to maintain highly saturated, non-iridescent structural colors over a wide angular range. These metasurface-based structural colors are discussed in the non-iridescent section of this review.

**Figure 8:**
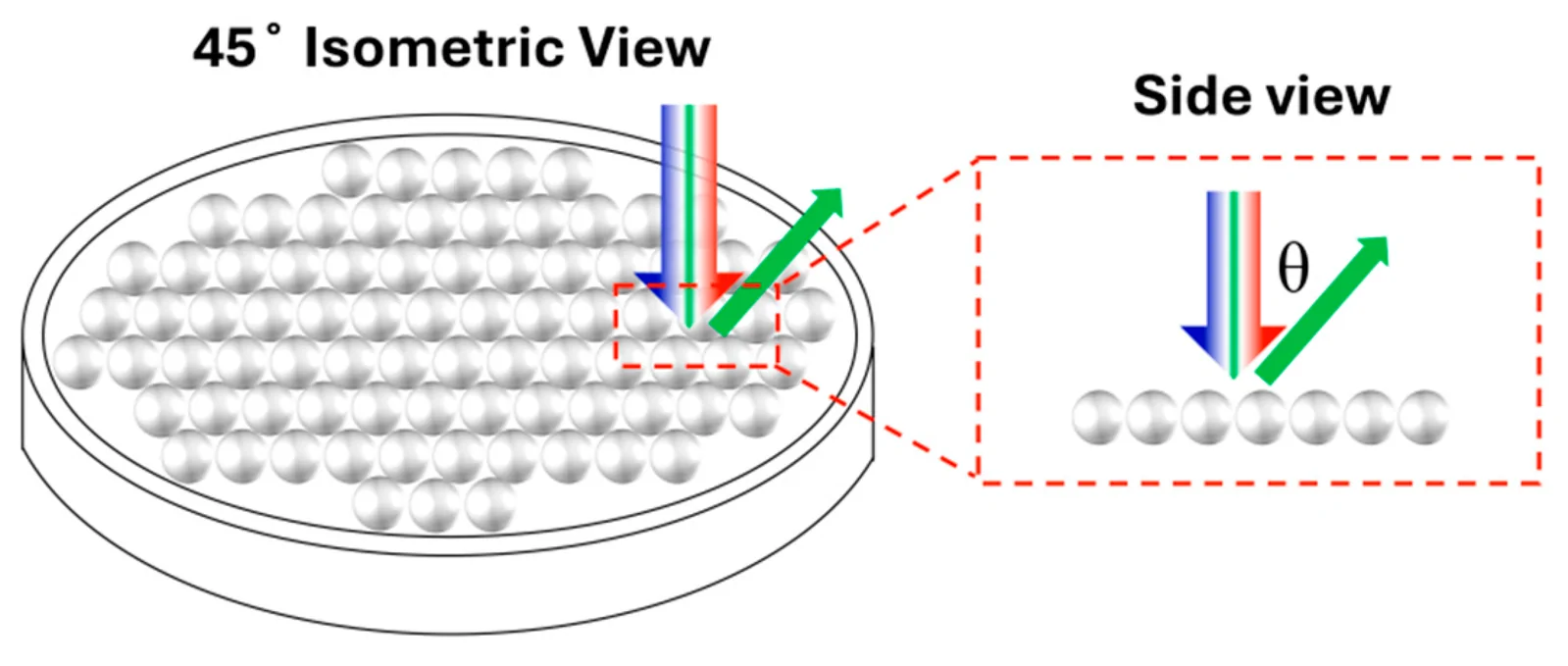
Schematic of two-dimensional PCs formed by a single layer of self-assembled microspheres. The arrows represent the incident/diffracted light.

2D PCs are widely used to fabricate structural colors, and the most common structure consists of a single layer of self-assembled microspheres, as shown in Figure 8. Because the microspheres form only a single layer, the structure lacks periodicity in the third dimension and exhibits periodicity only within the plane. During the fabrication of two-dimensional colloidal PCs, the Marangoni effect plays a key role in regulating the spatial arrangement of the microspheres and the interfacial flow during self-assembly [67]. The Marangoni effect is a mass transport phenomenon driven by surface tension gradients at the liquid interface. Gas–liquid interfacial self-assembly is one of the most widely used methods for fabricating two-dimensional colloidal PCs for structural color applications.

For example, Zhao et al. utilized poly(styrene/2-hydroxyethyl methacrylate) (PSH) microspheres to fabricate a 2D PSH PC film with angle-dependent structural colors through air-liquid interfacial self-assembly using water-to-ethanol volume ratios ranging from 10:1 to 1:3. By varying the styrene content, the PSH microsphere diameter was tuned to 240, 300, and 380 nm, thereby adjusting the structural colors of the PC [68]. Similarly, Tu et al. synthesized SiO_2_ microspheres with diameters of 250, 280, and 300 nm using the Stöber method and assembled them into an angle-dependent 2D PC film on a glass substrate through air-liquid interfacial self-assembly. They further infiltrated the interstitial spaces of the SiO_2_ 2D PC with a photocurable prepolymer, followed by HF etching to remove the SiO_2_ microspheres and produce a 2D inverse opal structure with periodically arranged spherical cavities [69]. Compared with the original SiO_2_ 2D PC, the inverse opal structure exhibited a higher refractive index contrast and stronger color contrast.

For a hexagonal close-packed microsphere array, the diffraction wavelength of a 2D PC is given by Equation (6) [69].

(6) $$m\lambda =\sqrt{3}nDsin\theta$$

where *m* is a positive integer, D represents the microsphere diameter, θ is the angle of incidence (as shown in Figure 8), λ is the diffraction wavelength, and *n* is the refractive index of the surrounding medium [69]. It is worth noting that, unlike thin-film interference or 1D PCs, where increasing the angle between the viewing direction and the surface normal causes the reflected wavelength to decrease (blueshift) [70], the reflected wavelength of 2D PCs increases with viewing angle (redshift) [69]. This difference arises from their distinct color-generation mechanisms. Structural colors produced by thin-film interference originate from phase differences caused by the optical path differences in reflected light at multiple interfaces. In contrast, the structural colors of 2D PCs originate from Bragg diffraction, which satisfies the in-plane momentum conservation condition imposed by the periodically modulated dielectric structure. Because 2D microsphere PCs consist of only a single layer and can be fabricated by simple self-assembly, their fabrication process is relatively simple and fast. However, the choice of microsphere materials is relatively limited, making it difficult to achieve structural colors with very high color contrast.

**Figure 9:**
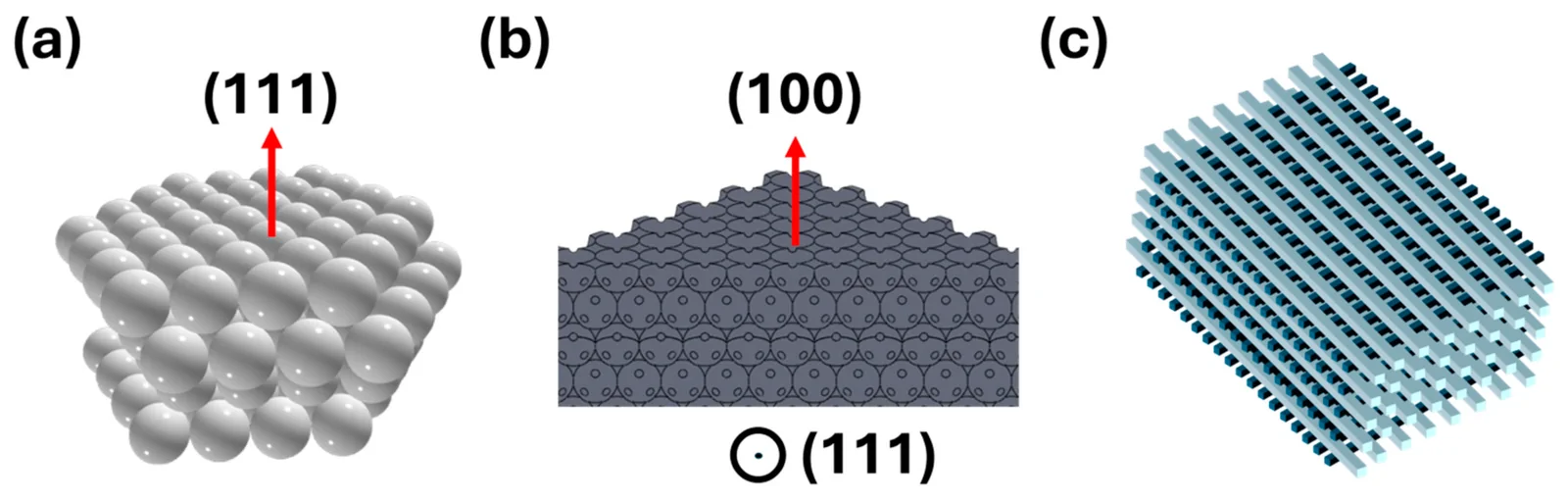
The schematic of 3D PCs constructed by (**a**) opal, (**b**) inverse opal, and (**c**) woodpile structures.

3D PCs are structures in which the dielectric constant or refractive index is periodically modulated in all three spatial dimensions on the wavelength scale. Compared with the multilayer interference of 1D PCs and the planar diffraction of 2D PCs, the most significant advantage of 3D PCs is their three-dimensionally interconnected pore network, which allows gas or liquid molecules to rapidly diffuse throughout the entire structure. This architecture provides a high specific surface area and enables rapid color responses. In addition, when the 3D lattice is subjected to external stimuli, its lattice constant can be tuned in three dimensions, making 3D PCs highly suitable for high-sensitivity strain, humidity, and biomedical sensors [71]. When a 3D PC does not exhibit a complete PBG, its structural color is mainly produced by Bragg diffraction arising from the periodic lattice. The diffracted light undergoes coherent interference, producing angle-dependent iridescent structural colors. Non-iridescent 3D PCs are discussed in the next section. Based on their geometric structures, 3D PCs used for structural color applications can be broadly classified into three categories: opal structures, inverse opal structures, and woodpile structures, as shown in Figure 9a, Figure 9b and Figure 9c, respectively. Each structure exhibits distinct optical properties because of its unique three-dimensional architecture.

Opal structures are typical 3D PCs formed by the close packing of microspheres, such as PS or silica microspheres, into a hexagonal close-packed (HCP) lattice. The diffraction behavior of these structures is primarily described by the Bragg-Snell law, as given in Equation (7) [72,73].

(7) $$m\lambda =2d_{hkl}\sqrt{n_{eff}^{2}-{sin}^{2}\theta_{hkl}}$$

where λ is the diffraction peak wavelength, $d_{hkl}$ is the interplanar spacing of a specific crystal plane, $\theta_{hkl}$ is the angle of incidence measured with respect to the normal of the (hkl) crystal plane, and $n_{eff}$ is the effective refractive index of the colloidal crystal, which can be calculated using a volume-fraction-weighted average of the refractive indices of the microspheres and the surrounding matrix. For the (111) crystal plane of a HCP structure, d_111_ is given by Equation (8).

(8) $$d_{111}=\sqrt{\frac{2}{3}}D$$

where D is the microsphere diameter. In self-assembled opal PCs, the (111) crystal plane is typically the dominant diffraction plane because of the HCP arrangement of the microspheres. It is worth noting that, unlike single-layer 2D PCs, opal structures exhibit a blueshift in the reflected wavelength as the angle of incidence, $\theta_{hkl}$ in Equation (7) increases.

Microsphere colloids used to fabricate opal structures have been widely explored as structural color inks. After solvent evaporation, the microspheres spontaneously self-assemble into an opal structure, generating structural colors. This simple fabrication process offers broad potential for printing and writing applications [74]. For example, Chi et al. used a 20 wt% SiO_2_-EtOH suspension as a fast-drying 3D PC ink for writing applications [75]. Han et al. used a poly(methyl methacrylate) (PMMA) colloidal suspension to fabricate large-area 3D PCs on a low-cost, easily removable porous paper substrate. They then coated the PC layer with a transparent encapsulation layer of the high-surface-tension waterborne acrylic resin poly(acrylic acid) (PAA). After removing the porous paper substrate, a flexible PC-polymer composite film was obtained [76]. Li et al. presented a printable meta-assembly strategy that combined self-assembled PS nanoparticle lattices with a PDMS-based microconcave optical interface. The periodic nanolattice provided PBG-based reflection, whereas the curved interface introduced TIR-mediated multibounce interference, enabling synergetic structural coloration and continuous roll-to-roll fabrication of meter-scale prints with single-pixel customization, including a 1.2 m film containing approximately 4 × 10^7^ meta-assemblies [77]. These structures are typically fabricated by bottom–up colloidal self-assembly. Although this approach is low-cost and suitable for large-area fabrication, the relatively low refractive index contrast generally results in a narrow PBG. Consequently, the resulting structural colors usually exhibit relatively low color contrast and saturation.

Inverse opal structures (IO) are shown in Figure 9b. An IO structure is typically fabricated by using silica or polymer microspheres as sacrificial templates. The interstitial spaces are infiltrated with a dielectric material, and the microsphere template is subsequently removed by chemical etching or high-temperature calcination, leaving a three-dimensionally interconnected network of periodically arranged air pores. Compared with opal structures, IO structures exhibit a higher refractive index contrast and greater porosity, making their optical response highly sensitive to changes in the refractive index of the surrounding medium. When the pores are filled with or adsorb specific gases or liquids, the resulting change in n_eff_ causes a significant shift in the diffraction peak. This property makes IO structures highly attractive for chemical sensing, biosensing, and smart color-changing labels [78]. Like opal structures, IO structures also follow the Bragg-Snell law. As the angle of incidence (θ) increases, the optical path difference decreases, resulting in a blueshift of the reflected wavelength. During fabrication, the opal template is typically assembled along the (111) crystal plane, and the corresponding IO structure also forms along the (111) crystal plane. Consequently, the replicated IO structure also exposes the (111) plane. As shown in Figure 9b, this surface exhibits the characteristic morphology in which each spherical cavity is connected to three neighboring cavities through three openings, corresponding to the (111) plane viewed normal to the surface. A 3D model of the IO structure is provided as an STL file in Supplementary Materials.

Transition metal oxides such as titanium dioxide TiO_2_ [79], zinc oxide ZnO [80], and zirconium dioxide ZrO_2_ [81] are among the most widely used materials for fabricating IO structures. These materials are typically infiltrated into the interstitial spaces of polymer colloidal crystal templates, such as PS or PMMA, by the sol–gel method [82] or atomic layer deposition (ALD) [83]. The polymer templates are then removed by high-temperature calcination or chemical etching to produce the IO structure. Because of the large refractive index contrast between the air pores (n = 1.0) and the high-refractive-index solid framework, IO structures exhibit enhanced reflectivity, color saturation, and visual contrast [84]. In recent years, highly biocompatible materials such as silk fibroin [85,86] and hydrogels [87,88,89,90] have also been used to fabricate IO structures for structural color applications.

For example, Qiu et al. fabricated opal templates using SiO_2_ microspheres with average diameters of 182.6, 232.6, and 285.3 nm. The SiO_2_ colloidal suspension was injected into a microneedle mold to form opal-structured microneedles. A hydrogel precursor solution was then introduced and cured under ultraviolet (UV) irradiation. Subsequently, the hydrogel microneedles were immersed in a 3% hydrofluoric acid (HF) solution to completely dissolve the SiO_2_ microspheres, leaving a highly ordered three-dimensional porous IO structure within the hydrogel. This IO structure produced structural colors through the PC effect, giving the microneedles their characteristic appearance [87].

It is worth noting that removing the sacrificial microsphere template remains one of the most critical steps in fabricating IO structures. During this process, such as high-temperature calcination for inorganic materials or chemical etching for polymer templates, the solid framework may develop microcracks or even undergo local pore collapse because of volume shrinkage, non-uniform surface tension, or internal stress concentration. These structural defects disrupt the long-range optical coherence of the periodic lattice and increase background light scattering, thereby reducing the reflection intensity and color purity of the iridescent structural colors. Therefore, developing milder template-removal processes with minimal shrinkage, improving the structural uniformity of large-area fabrication, and enhancing the mechanical durability of IO structures while preserving their sensitive iridescent optical response remain the major challenges for their practical applications.

Woodpile structures are formed by precisely stacking multiple layers of mutually perpendicular dielectric nanorods, as shown in Figure 9c. When light is incident on a woodpile structure, it propagates through a three-dimensionally periodic refractive index distribution. Reflections from the interfaces between adjacent dielectric layers interfere with one another, and constructive interference at specific wavelengths produces strong reflection. By controlling the dimensions and arrangement of the dielectric rods, woodpile structures can be designed with customized three-dimensional geometries. When the lattice constant is comparable to the wavelength of visible light, the structure exhibits structural color. Because woodpile structures possess highly ordered three-dimensional periodicity, their fabrication generally relies on advanced micro- and nanofabrication techniques, such as two-photon polymerization [91,92,93], direct ink writing [94], and UV-soft nanoimprint lithography [95]. However, fabricating woodpile structures with lattice constants suitable for visible structural colors remains challenging because of the resolution limits of current fabrication technologies.

To overcome the limited resolution of commercially available two-photon polymerization systems, Liu et al. fabricated a woodpile structure in IP-Dip photoresist with an initial lateral lattice constant a_xy_ = 1.57 μm, which was readily achievable using a commercial system. Although this lattice constant was too large to produce structural color, thermal shrinkage reduced the lattice constant by 78% to approximately 350 nm, enabling structural color generation. The same approach was also applied to fabricate three-dimensional structural color microstructures, including a 54 μm-high Eiffel Tower and 20 μm-high Chinese characters [96]. Liu et al. from another research group developed a solvent-free hydrogel formulation by directly dissolving acrylic acid (AAC) and polyvinylpyrrolidone (PVP) through ultrasonication without adding a solvent. The hydrogen bonding between AAC and PVP significantly increased both the density of photoreactive groups and the viscosity of the resin, allowing femtosecond laser direct writing with an ultrahigh resolution of 100 ± 2 nm. Using this material, woodpile structures with lateral periods of 0.7–1.2 μm and a fixed axial period of 1 μm were directly fabricated by two-photon polymerization without any post-fabrication shrinkage treatment [97].

Because of their high geometric programmability, woodpile structures allow precise control of three-dimensional lattices, including the incorporation of designed defects, providing exceptional flexibility for tailoring structural colors and optical responses. However, their fabrication still requires expensive equipment and time-consuming layer-by-layer processing. As a result, woodpile structures are currently used mainly for high-performance photonic devices and micro-optical display components rather than large-area decorative applications.

#### 2.1.3. Diffraction Gratings

**Figure 10:**
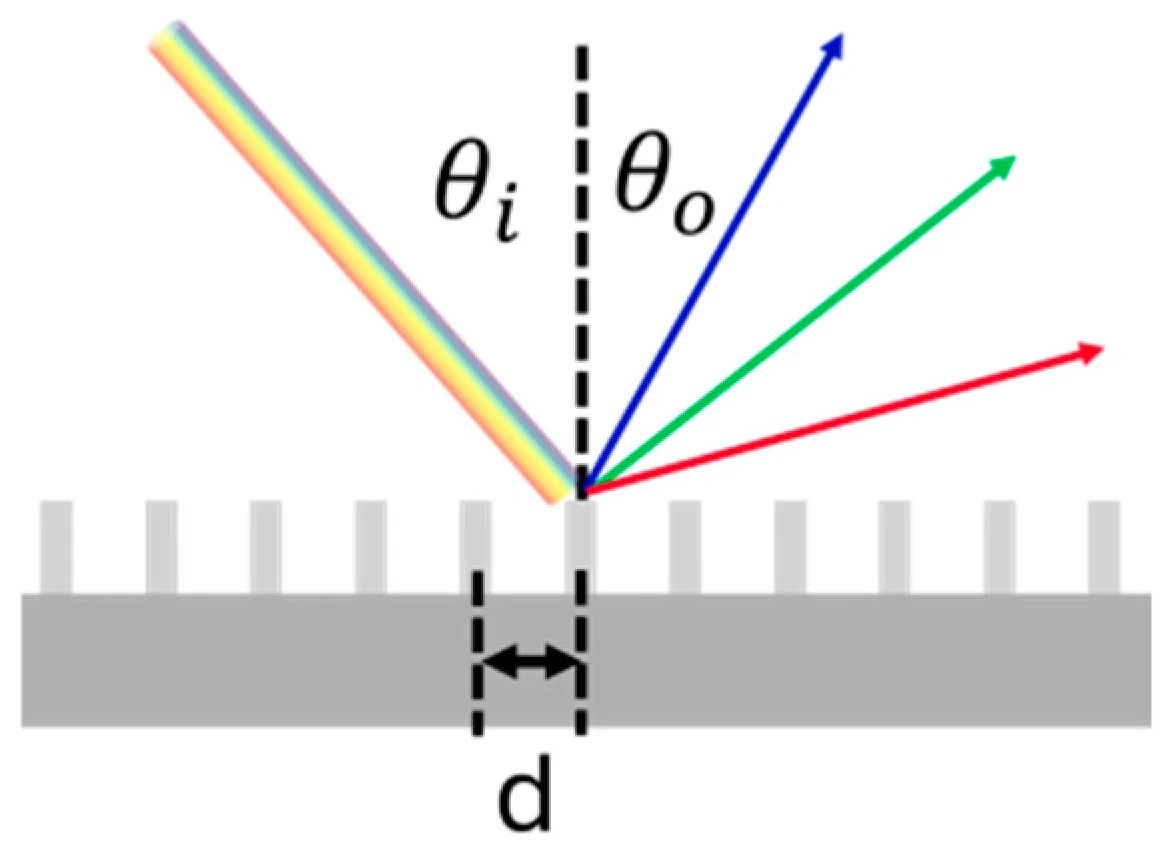
Schematic of the incident light and the diffracted light on a grating. The arrows represent incident/refracted light.

Grating structures are periodic micro- and nanostructures composed of a series of parallel lines or grooves. Although their geometry is similar to that of 1D or 2D PCs, gratings emphasize a different optical mechanism. Their primary optical effect is diffraction, which is why they are commonly referred to as diffraction gratings. When light is incident on a grating, the periodic structure diffracts the incident light into different directions, producing wavelength dispersion and angle-dependent structural colors, as shown in Figure 10.

For a diffraction grating with a uniform period, the relationship between the grating period and the diffraction wavelength is given by the grating equation as Equation (9) [98].

(9) $$m\lambda =d(sin\theta_{i}+sin\theta_{o})$$

where *m* is a positive integer, λ is the diffraction peak wavelength, d is the grating period, $\theta_{i}$ is the angle of the incident light, and $\theta_{o}$ is the observation angle. Under fixed illumination and observation conditions, decreasing the grating period shifts the diffraction wavelength toward shorter wavelengths, resulting in a blueshift of the perceived color. By precisely controlling the grating period, line width, and height, the structural color can be effectively tuned. Similarly, increasing the observation angle causes a redshift in the diffraction wavelength, producing iridescent structural colors. Optical disks are among the most common artificial diffraction gratings found in everyday life, and their low cost is largely attributable to large-scale nanoimprint manufacturing [99].

In addition to replicating commercial grating structures, techniques such as laser direct writing [100], 3D printing [101], and elliptical vibration cylindrical turning [102] have also been used to fabricate diffraction gratings for structural color applications. For example, Somers et al. proposed a superimposed grating geometry consisting of multiple square-wave phase gratings to improve mechanical stability. The structure was fabricated by two-photon polymerization (TPP). Using particle swarm optimization (PSO) combined with finite-difference time-domain (FDTD) simulations, they optimized both conventional single-line gratings and the proposed superimposed grating to develop zero-order transmission optical filters with high color purity in the visible wavelength range. Numerical simulations and experimental results confirmed that the superimposed grating distributed the required phase modulation across three independent height levels and a wider structural area. This design significantly improved its mechanical resistance to capillary forces. It also overcame the theoretical limitation of conventional square-wave gratings, which cannot achieve high color saturation in the cyan and green wavelength regions. As a result, the superimposed grating achieved improved color purity and optical performance across the visible spectrum [96]. Bae et al. demonstrated a vertically integrated multilayer diffraction grating fabricated by 3D printing, enabling additive color mixing within a single pixel. This approach addressed the difficulty of generating non-spectral colors, such as magenta, using diffraction gratings without sacrificing spatial resolution. The researchers fabricated a bilayer grating composed of orthogonally arranged PS nanowire arrays. Because the two nanowire layers exhibited strong azimuthal selectivity, they could operate independently under angle-resolved illumination from two separate light sources with minimal optical interference. This design allowed diffracted red and blue light to overlap and mix within a single pixel smaller than 7 μm. Consequently, the structure produced colors such as magenta, yellow, and cyan, while also allowing continuous control of color intensity [101]. Yang et al. proposed an elliptical vibration cylindrical turning (EVCT) method based on a conventional turning system. This method enabled the large-area fabrication of nanogratings on cylindrical surfaces for structural color applications. By adjusting the tool-tip position, the equivalent clearance angle could be varied, allowing gratings with different blaze angles and color saturations to be fabricated. The resulting cylindrical structures could also serve as master molds for roll-to-plate imprinting [98]. In recent years, diffraction gratings for structural color applications have progressed from conventional single-layer structures with limited spectral control toward multidimensional geometric designs, high-resolution full-color rendering, and large-scale fabrication on curved surfaces.

In this section, the structures used to generate iridescent structural colors are reviewed and compared in Table 1. Iridescent structural colors are commonly generated by well-defined micro- and nanostructures that support angle-dependent interference or diffraction, resulting in dynamic optical responses. As the viewing angle changes, the reflected wavelength undergoes a blueshift or redshift, producing distinctive dynamic visual effects. These characteristics make iridescent structural colors particularly attractive for applications such as angle-dependent anti-counterfeiting, smart optical sensing, and dynamic photonic devices. However, iridescent structural colors represent only one approach to structural color design. For applications that require stable color under different viewing angles, such as decorative coatings and optical displays, non-iridescent structural colors have also been extensively developed. The following sections discuss the color-generation mechanisms and design strategies of non-iridescent structural colors.

### 2.2. Non-Iridescent: Angle-Stable Coloration

Non-iridescent structural colors exhibit angle-independent optical properties and maintain stable colors over a wide range of viewing angles. Unlike iridescent structural colors, whose spectral responses generally vary with the illumination or viewing angle because of angle-dependent interference or diffraction, non-iridescent structural colors commonly suppress such angular variation through isotropic scattering, localized optical resonances, or other structural design strategies. To achieve angle-independent coloration, current fabrication strategies generally follow three main approaches. The first approach uses independent resonators to selectively absorb or scatter specific wavelengths. The second employs disordered or quasi-amorphous structures with short-range order and long-range disorder, thereby suppressing the angle dependence associated with Bragg diffraction through isotropic scattering. The third approach utilizes engineered metasurfaces composed of subwavelength arrays to precisely manipulate near-field light, minimizing spectral shifts over a wide range of viewing angles. Together, these optical mechanisms provide the physical basis for non-iridescent structural colors and enable applications such as decorative coatings and wide-viewing-angle optical displays.

Because non-iridescent structural colors rely on localized optical structures rather than long-range periodicity, they can achieve extremely high spatial resolution and, in some cases, even exceed the optical diffraction limit [103]. It is worth noting that several excellent review articles have summarized recent advances in plasmonic and dielectric structural colors from the perspective of material systems [104,105,106]. In contrast, this review classifies non-iridescent structural colors according to their structural design rather than their constituent materials. Regardless of whether the structures are based on plasmonic or dielectric materials, they are categorized into three groups: independent resonators, disordered structures, and engineered metasurfaces. The following sections provide a comprehensive overview of the color-generation mechanisms, design strategies, and recent advances in these three categories.

#### 2.2.1. Independent Resonators

The optical response of independent resonators originates from localized resonances within individual subwavelength structures. These resonators selectively absorb or scatter specific wavelengths through strongly confined electromagnetic modes. Because the optical field of each resonator is highly localized, the overall extinction spectrum can be regarded as the linear superposition of the responses from individual resonators, provided that the spacing between neighboring resonators is larger than the near-field coupling distance. This weak coupling effectively suppresses angle-dependent optical interactions, resulting in non-iridescent structural colors. Depending on the material system, the localized optical response is mainly governed by two mechanisms: localized surface plasmon resonance (LSPR) and Mie resonance in high-refractive-index dielectric resonators.

**Figure 11:**
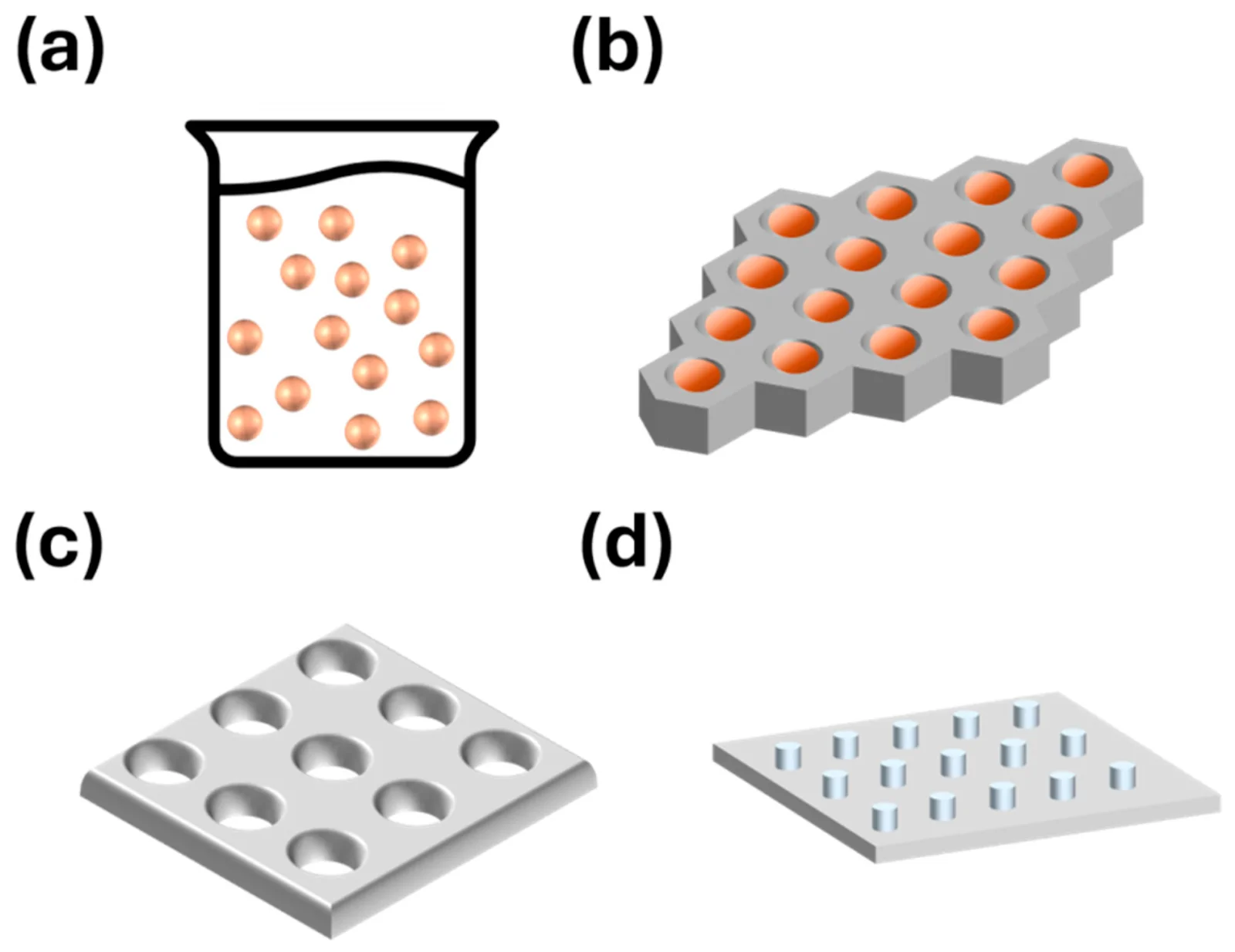
Schematic of independent resonators based on (**a**) nanoparticle colloids, (**b**) nanoparticles separated by nanostructures, (**c**) nanopores array, and (**d**) nanopillar array.

LSPR occurs when the electric field of the incident light strongly couples with the free electrons in a metallic nanostructure, inducing collective electron oscillations. When the resonance condition is satisfied, the electromagnetic field near the resonator is enhanced by several orders of magnitude. Consequently, the extinction cross-section can greatly exceed the geometric cross-section, producing strong wavelength-selective absorption and intense near-field enhancement. Among independent resonators, silver and gold nanoparticles are the most representative and widely studied systems, as shown in Figure 11a. Their high free-electron densities support strong LSPR within the visible wavelength range, resulting in large extinction cross-sections and vivid structural colors. In addition, a wide variety of metallic nanoparticle geometries can be synthesized to tune the resonance wavelength, including nanospheres [107], nanorods [107], nanoprisms [108], nanostars [109], and nanodumbbells [110]. To improve their colloidal stability and prevent particle aggregation, various dispersants have also been developed [111].

However, because it is difficult to precisely control the spatial distribution of metallic nanoparticles in colloidal solutions, solid substrates have been developed to immobilize and isolate individual nanoparticles. For example, Nam et al. dispersed single silver or gold nanoparticles into the nanopores of anodic aluminum oxide (AAO). Each AAO nanopore accommodated only one metallic nanoparticle, resulting in a uniform and well-isolated nanoparticle array, as shown in Figure 11b [112]. In addition to metallic nanoparticles, advances in bottom–up fabrication have enabled a variety of uncoupled subwavelength arrays, including nanopore (Figure 11c) [113], nanopillar (Figure 11d) [114], and nanodesk arrays [115]. For example, Lu et al. proposed a subwavelength nanopore array for transmissive plasmonic structural colors with ultrahigh spatial resolution. They systematically investigated both the theoretical performance and the large-scale fabrication feasibility of nanoimprint lithography (NIL). This approach was applied to structural color printing of miniaturized images, achieving an ultrahigh spatial resolution of up to 127,000 DPI [113]. Zhou et al. developed a plasmonic reflector consisting of three layers deposited sequentially on a glass substrate by physical vapor deposition (PVD): silver nanoparticles, an aluminum oxide dielectric spacer, and a thick silver reflector. The structure generated bright structural colors through LSPR coupling between the silver nanoparticles and the underlying silver film. This design demonstrated excellent performance for rendering intricate patterns, including school emblems, flowers, and red dragons [115].

In recent years, deep learning has also been applied to predict the relationship between LSPR nanostructures and the resulting structural colors [116,117]. Liu et al. proposed an inverse design strategy based on a bidirectional deep neural network for silver subwavelength nanopore arrays. By jointly learning the transmission spectra and the corresponding structural colors, the model successfully addressed the many-to-one mapping problem commonly encountered in the inverse design of optical structures [116]. Driven by the growing demand for localized electromagnetic field enhancement, LSPR technologies have advanced rapidly in recent years. Numerous plasmonic nanostructures have been developed, providing a solid foundation for structural color applications based on LSPR [118,119,120,121].

LSPR offers great potential for sensing and catalysis. However, the strong optical extinction of metals also results in significant absorption losses in the visible wavelength range. Therefore, high-refractive-index dielectric materials with negligible intrinsic absorption in the visible region have been widely used to fabricate independent resonators for structural color generation. When the dimensions of a dielectric resonator are comparable to the wavelength of light, its optical response is accurately described by Mie scattering theory [122,123]. When incident light enters a high-refractive-index dielectric resonator, such as a microsphere or micropillar, particularly with a refractive index (n) greater than 3, the light is strongly confined within the structure by total internal reflection, giving rise to displacement currents [124]. According to multipole theory, these currents simultaneously excite electric dipole (ED) and magnetic dipole (MD) resonances. The interference between the ED and MD resonances enables highly directional forward or backward scattering, as described by the first and second Kerker conditions. As a result, dielectric resonators can produce low-loss, high-saturation, non-iridescent structural colors.

Silicon is one of the most widely studied materials for Mie-resonant dielectric structures because of its excellent compatibility with existing semiconductor fabrication technologies. For example, Yamana et al. developed an aqueous inkjet ink based on crystalline silicon nanoparticles (Si NPs). To prevent particle aggregation and the resulting color degradation, the Si NPs were encapsulated with a silica shell to form Si@SiO_2_ core–shell particles. This design preserved the Mie scattering characteristics of individual nanoparticles after they were embedded in an acrylic resin matrix [125].

Although silicon has a high refractive index, its extinction coefficient is relatively large, especially at wavelengths below 450 nm. Consequently, achieving full-spectrum structural colors using silicon remains challenging [126,127]. To overcome this limitation, dielectric materials with lower optical absorption, including ZnS [128,129], ZnO [130], TiO_2_ [131], and Cu_2_O [132], have been increasingly investigated as Mie resonators. For example, Liu et al. used ZnS polycrystalline colloidal spheres with a refractive index of approximately 2.1 as Mie resonators and developed a photocurable hydrogel ink for 3D printing. By incorporating only 0.5 wt% ZnS colloidal spheres into the hydrogel precursor and adding carbon black to suppress multiple scattering, vivid and uniform structural colors were obtained without any post-treatment or shrinkage [129]. Ren et al. used ZnO polycrystalline spheres with a moderate refractive index of approximately 1.7 and negligible absorption in the visible wavelength range as Mie resonators. When the ED resonance was located in the red wavelength region, the higher-order multipole resonances were shifted into the ultraviolet region. This design enabled a high-saturation pure red structural color in a monolayer Mie resonator film. In addition, the reflection wavelength could be precisely tuned by varying the diameter of the ZnO nanospheres from 320 to 630 nm. As a result, full-color structural colors covering deep blue, green, yellow, orange, red, and purple were achieved, spanning approximately 90% of the sRGB color gamut [130]. Kim et al. utilized TiO_2_ which has a moderate refractive index of approximately 2 and negligible absorption in the visible wavelength range. By changing the resonator geometry from single particles to trimers, the magnetic resonance wavelength shift in the TiO_2_ trimers became 5.2 times greater than that of silicon structures with the same dimensions. In addition, the TiO_2_ trimers exhibited stronger scattering intensity [131].

In summary, independent resonators provide a robust and intuitive strategy for generating non-iridescent structural colors by eliminating the need for long-range periodic structures. By confining light–matter interactions within individual subwavelength resonators, either through the localized free-electron oscillations of LSPR or the low-loss ED and MD resonances of dielectric materials, the overall optical response becomes the superposition of independent resonator responses. Recent advances have expanded the available material systems from plasmonic metals to low-loss dielectric materials while integrating deep learning and printable material systems to accelerate practical applications. However, independent resonators still face several challenges. In bottom–up colloidal systems, maintaining sufficient spacing between neighboring particles is essential to suppress near-field coupling and prevent color degradation caused by particle aggregation. In contrast, top–down fabricated uncoupled arrays can achieve ultrahigh spatial resolution but still rely on expensive, low-throughput nanofabrication techniques, limiting their large-scale industrial implementation.

#### 2.2.2. Disordered or Quasi-Amorphous Structures

**Figure 12:**
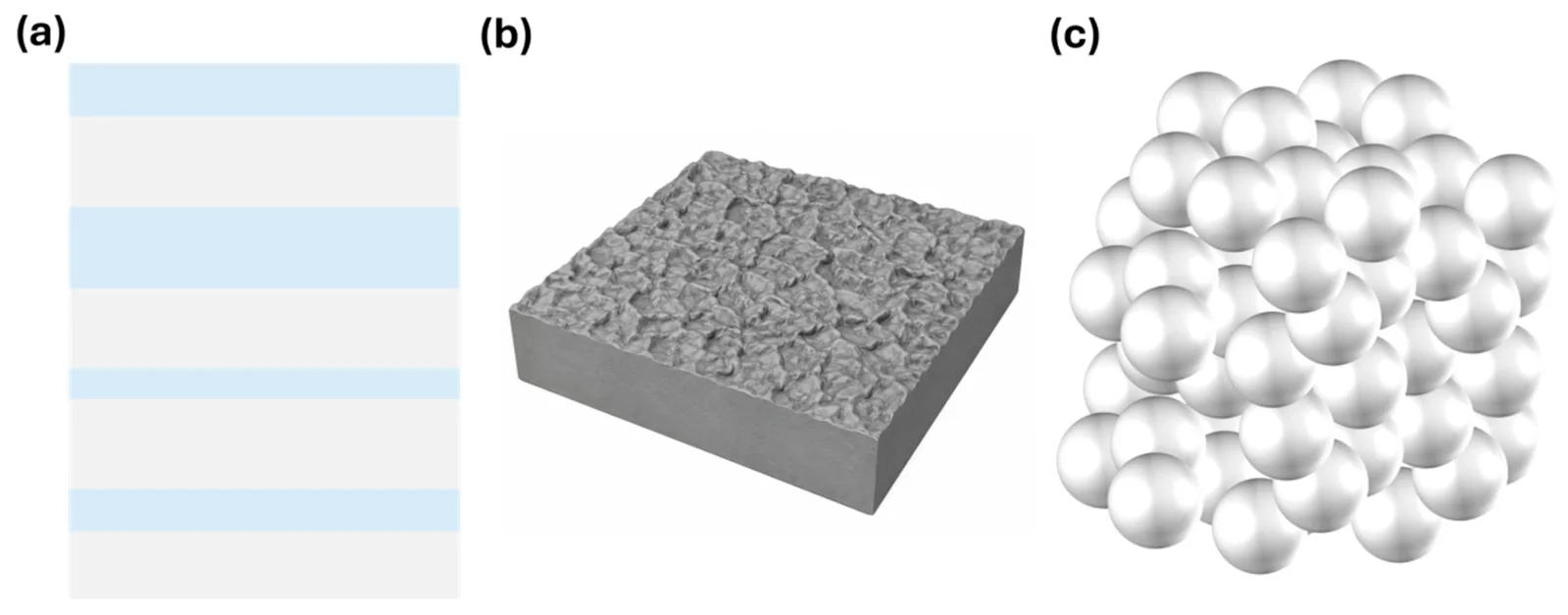
Schematic of (**a**) 1D, (**b**) 2D, and (**c**) 3D disorder structures for generating non-iridescent structural coloration.

While independent resonators confine optical responses within individual subwavelength structures, disordered or quasi-amorphous structures generate non-iridescent structural colors through collective light scattering from multiple scattering units. The underlying mechanism relies on the coexistence of short-range order (SRO) and long-range disorder [133]. At the microscopic scale, neighboring structural units maintain a nearly uniform spacing, which promotes constructive interference at selected wavelengths. At the same time, the absence of long-range periodicity suppresses the formation of well-defined Bragg diffraction peaks. Instead, the optical response becomes a broad scattering feature with much weaker angle dependence [134], giving rise to additive color effects [135]. As a result, light is scattered nearly isotropically, producing a macroscopic appearance that remains nearly unchanged over a wide range of illumination and viewing angles. It is important to note, however, that the color-generation mechanism in these structures is still fundamentally based on light interference and diffraction. Structural disorder does not eliminate iridescence completely but instead reduces its angular dependence. Therefore, the distinction between iridescent and non-iridescent structural colors should be regarded as a continuum rather than two strictly separate categories. The degree of angle dependence depends on the level of structural disorder. Quantitative characterization is therefore important for evaluating the non-iridescent performance of these structures, and the relevant methods are discussed in the next section. Depending on the spatial dimensionality of the structural disorder, these architectures can be classified into 1D, 2D, and 3D structures, as shown in Figure 12.

1D quasi-amorphous structures typically consist of multilayer stacks of alternating high- and low-refractive-index materials with intentionally varied layer thicknesses, as shown in Figure 12a. Unlike periodic 1D Bragg reflectors, which exhibit strong iridescence, these structures introduce controlled thickness fluctuations to reduce angle dependence. Their structural colors arise from the interference of light reflected at the interfaces between layers with different thicknesses. If the thickness variation is too large, the reflection spectrum becomes broadband and eventually appears white. In contrast, when the layer thicknesses fluctuate within a narrow statistical distribution, the reflection band remains sufficiently narrow to produce a distinct color while greatly reducing its angle dependence. This strategy is commonly found in nature. For example, the scales of the beetle *Chrysochroa raja* (*C. raja*) contain multilayers with disordered thicknesses that produce structural colors. Although the coloration is still based on thin-film interference, the beetle exhibits an overall green appearance with only weak angle dependence [136]. Mouchet et al. reported that the standard deviation of the normalized layer-thickness distribution in *C. raja* ranged from 4% to 22% of the median thickness. Within this range, the structural colors maintained high saturation. However, further increasing the degree of disorder gradually reduced the color saturation of the average reflection spectrum [137].

Although similar structures are widely found in nature, fabricating dozens of dielectric layers remains impractical for many artificial applications. Simpler structures that provide sufficiently low angle dependence at a lower fabrication cost are therefore more attractive. For example, Dong et al. proposed a self-assembly strategy for cellulose nanocrystals (CNCs) by introducing positively charged polydiallyldimethylammonium chloride (PDDA). The addition of PDDA induced electrostatic co-assembly with the negatively charged CNCs, disrupting their original long-range periodicity. When the PDDA content exceeded 1.13 wt%, the chiral structure of the CNC film transformed into an amorphous arrangement. This transition suppressed Bragg reflection and produced vivid, angle-independent structural colors. Over a viewing-angle range of 0° to 60°, the reflection peak shifted by less than 10 nm [138]. In 1D quasi-amorphous structures, the structural disorder is confined to the z-direction, perpendicular to the film surface, while the xy plane remains highly ordered. As a result, these structures exhibit excellent color uniformity. However, their layered geometry makes them less suitable for coating irregular curved surfaces.

2D quasi-amorphous structures confine structural disorder to a single plane while maintaining order in the out-of-plane direction. These structures are typically formed as single-layer films. Although colloidal particles can be used to fabricate 2D quasi-amorphous structures for non-iridescent structural colors [139], they offer few advantages over their 3D counterparts and therefore have attracted relatively limited attention. The non-iridescent structural colors generated by quasi-amorphous colloidal assemblies are discussed in detail in the following section on 3D quasi-amorphous structures. Compared with quasi-amorphous colloidal assemblies, isotropic wrinkle structures are the most representative example of 2D quasi-amorphous architectures, as shown in Figure 12b [140,141]. Isotropic wrinkles are formed when the surface of a material develops irregular wavelengths and amplitudes, producing patterns with short-range order but no long-range order. These structures are also referred to as labyrinth-like, random-like, or spinodal-like patterns and exhibit polycrystalline characteristics with domains of multiple orientations [140]. In contrast, directional wrinkles resemble diffraction gratings and behave as one-dimensional ordered structures, producing iridescent colors with strong angle dependence [142]. Isotropic wrinkles, however, maintain only local periodicity between neighboring crests and troughs. This short-range order enables nearly isotropic diffraction of specific wavelengths, greatly reducing the angle dependence of the resulting structural colors. For example, Tan et al. formed a thin, stiff surface layer on an unstretched circular PDMS substrate by oxygen plasma treatment. The bilayer structure was subsequently heated to 200 °C for 30 min, generating mechanical instability through thermal expansion during heating and contraction during cooling. This process produced isotropic reverse wrinkles with random orientations. Under laser illumination, these wrinkles generated a single-frequency, centrosymmetric ring-shaped diffraction pattern. Macroscopically, the isotropic wrinkle structure uniformly scattered the structural colors produced by thin-film interference in all directions. As a result, the diffraction intensity remained nearly unchanged as the viewing azimuth angle was varied [143]. Similarly, Zhou et al. fabricated disordered wrinkle films in a bilayer system consisting of a photopolymer film on a carbon nanotube-polydimethylsiloxane substrate through high-temperature treatment. The resulting labyrinth-like wrinkle morphology scattered structural colors that would otherwise be observed only at the specular reflection angle, allowing them to be viewed uniformly over a much wider angular range. Both experiments and finite element simulations demonstrated that the wrinkle structure increased the viewing angle from 4° for a flat film to 55°. Moreover, the viewing angle could be precisely controlled by increasing the processing temperature, which increased the wrinkle amplitude-to-wavelength ratio [144].

3D quasi-amorphous structures, particularly those composed of dielectric microspheres, are currently among the most widely studied and commercially promising platforms for non-iridescent structural colors [145,146]. They consist of densely packed colloidal spheres that exhibit short-range order but long-range disorder. Long-range disorder can be achieved in two ways. The first approach uses monodisperse particles assembled into non-close-packed structures [147]. The second uses polydisperse particles with different diameters to suppress long-range ordering during self-assembly [148]. The former was first described as “photonic glass” by García et al. in 2007 [147]. Over time, however, the term “photonic glass” has also been used to describe disordered colloidal assemblies composed of particles with different sizes [149]. The non-iridescent structural colors of 3D quasi-amorphous structures originate from isotropic scattering and interference arising from their short-range ordered microstructure rather than from incoherent scattering by air voids within the material [150]. This indicates that short-range order is essential for generating non-iridescent structural colors, whereas completely random structures cannot produce the same optical effect [151]. Because colloidal particles naturally tend to assemble into close-packed ordered structures, additional strategies are required to suppress the formation of long-range order during self-assembly. These approaches disrupt the interactions between neighboring particles, resulting in quasi-amorphous structures with only short-range order. Current fabrication methods include spray coating [152], UV curing [153], and 3D printing [154].

For example, Hao et al. developed a carbon-encapsulated silica photonic glass pigment for structural color applications. The structure was fabricated by coating monodisperse silica microspheres, prepared by the Stöber method, with an alkylsilane hybrid shell. The coated particles were then carbonized in situ by calcination at 800 °C under a protective atmosphere. During this process, the organic components were removed and converted into a uniform amorphous carbon shell with nanoscale thickness control. The resulting microsphere suspension was deposited onto a substrate by spray coating, where the particles spontaneously assembled into a photonic glass with short-range order and long-range disorder, as confirmed by a ring-shaped 2D-FFT pattern. By adjusting the silica core diameter and carbon shell thickness, the reflection peak could be continuously tuned from 426 to 717 nm. In addition, the broadband absorption of the carbon shell effectively suppressed diffuse multiple scattering caused by the disordered structure, significantly improving the color saturation, particularly in the blue-green wavelength region. The optimized blue pigment exhibited a reflection peak at approximately 460 nm with a reflectance of about 28% [152]. Jeon et al. developed a metallodielectric photonic glass pigment based on Au-SiO_2_ core–shell particles. By adding approximately 0.9 mM NaCl, the Debye length was reduced, effectively suppressing electrostatic repulsion and allowing particles at a volume fraction of 34% to self-assemble into an amorphous photonic glass with short-range order in a refractive-index-matched UV-curable resin. The gold core selectively absorbed wavelengths below 500 nm, reducing the single-particle scattering background. By tuning the particle diameter from 160 to 230 nm, the researchers achieved highly saturated, angle-independent red structural colors. The resulting reflection spectrum exhibited a narrow bandwidth with a significantly reduced full width at half maximum (FWHM), and the reflection band was confined to 600–800 nm [153]. It is worth noting that, compared with ordered PCs, 3D quasi-amorphous structures generally exhibit lower color saturation and have greater difficulty producing highly saturated long-wavelength colors, particularly red. Therefore, current strategies often employ black backgrounds to suppress broadband scattering or selectively absorb blue light to enhance red structural colors.

In this section, disordered or quasi-amorphous structures, including 1D random multilayers, 2D isotropic wrinkle structures, and 3D photonic glasses, are reviewed as a scalable bottom–up approach for generating non-iridescent structural colors inspired by nature. By balancing short-range order with long-range disorder, these structures suppress the strong angle dependence associated with Bragg diffraction while maintaining selective reflection at specific wavelengths. As a result, they produce nearly omnidirectional structural colors with improved color saturation. Recent advances have further enhanced their optical performance while avoiding the low throughput and high cost associated with advanced nanofabrication techniques. Unlike disordered structures, which rely on the collective optical response of statistically distributed building blocks, engineered metasurfaces generate non-iridescent structural colors through the deterministic design of individual subwavelength elements. Rather than controlling light through structural disorder, metasurfaces precisely tailor the geometry of each unit cell to achieve the desired optical response. This concept forms the third major strategy for generating non-iridescent structural colors and is discussed in the following section.

#### 2.2.3. Engineered Metasurfaces

Standing in contrast to the statistical approach of quasi-amorphous structures, engineered metasurfaces provide a deterministic platform for manipulating light at the subwavelength scale. Rather than describing optical responses through effective-medium approximations, metasurfaces consist of spatially engineered arrays of subwavelength nanostructures, commonly referred to as meta-atoms [155,156]. By tailoring the geometry of individual meta-atoms, localized optical properties, including phase, amplitude, polarization, and resonance, can be precisely controlled. This deterministic design strategy enables structural color generation through engineered light–matter interactions, extending beyond conventional approaches based primarily on spectral filtering [157,158].

Engineered metasurfaces are generally regarded as the two-dimensional counterparts of bulk metamaterials. The modern development of metamaterials began with Pendry’s proposal of split-ring resonators (SRRs) in 1999, which enabled artificial magnetic responses at microwave frequencies [159]. Shortly afterward, Smith et al. combined SRRs with metallic wire arrays to realize a composite medium exhibiting simultaneously negative permittivity and permeability [160]. This work led to the first experimental demonstration of negative refraction by Shelby et al. in 2001 [161]. Although the early negative-index metamaterials developed by Smith et al. employed planar SRRs fabricated on printed circuit boards, these structures were periodically stacked to form three-dimensional effective media, allowing electromagnetic waves to propagate through the bulk and accumulate the required phase response. A major milestone was achieved in 2011 when Yu et al. introduced the generalized laws of reflection and refraction, establishing the modern framework for optical metasurfaces [162]. Their work demonstrated that a single layer of subwavelength nanoantennas could introduce abrupt phase discontinuities at an interface, enabling deterministic wavefront control without relying on phase accumulation through bulk propagation. Despite their outstanding optical performance, the large-scale fabrication of engineered metasurfaces remains challenging. Fabricating subwavelength meta-atoms with precisely defined nanoscale geometries typically relies on advanced nanofabrication techniques, most commonly electron-beam lithography (EBL) followed by reactive-ion etching (RIE) [163]. Because EBL is an inherently serial, low-throughput, and high-cost process, scaling metasurfaces to macroscopic areas remains difficult. Although high-throughput replication techniques such as NIL can improve manufacturing efficiency, fabricating the original master mold still requires sophisticated nanofabrication and substantial upfront investment [164,165]. Consequently, the widespread use of engineered metasurfaces for large-area decorative structural colors or commercial color printing remains economically challenging. To maximize the value of these fabrication processes, metasurface structural colors are increasingly integrated with additional optical functionalities, including information encoding [166], optical encryption [167], holography [168], or polarization control [169], thereby providing capabilities beyond passive color generation.

In summary, the development of non-iridescent structural colors represents a major shift from conventional iridescent systems by minimizing the dependence of optical appearance on long-range structural periodicity. As summarized in Table 2, the three major design strategies achieve angle-independent coloration through fundamentally different optical mechanisms and structural architectures.

Independent resonators rely on localized optical resonances within isolated subwavelength structures, providing excellent color purity and precise spectral tuning. However, their performance depends strongly on optical isolation between neighboring resonators, while high-resolution nanofabrication or particle stabilization remains a significant challenge. In contrast, quasi-amorphous structures suppress angle dependence through short-range order and long-range disorder, offering a scalable and cost-effective bottom–up approach for large-area structural coloration. Nevertheless, their color saturation, particularly in the long-wavelength region, is generally lower than that of ordered nanostructures. Engineered metasurfaces represent the most versatile platform by enabling deterministic control of phase, amplitude, and polarization at the level of individual meta-atoms.

Although they provide unparalleled optical functionality, their widespread application is still restricted by the high cost and limited throughput of current nanofabrication technologies. Rather than representing competing technologies, these three approaches should be viewed as complementary design strategies operating at different structural length scales, ranging from localized resonators and statistical disorder to deterministic wavefront engineering. Overall, these three approaches reflect different trade-offs between optical performance, fabrication complexity, scalability, and commercial applicability. Future developments are expected to combine the advantages of these complementary strategies to simultaneously achieve high color saturation, angle-independent optical responses, multifunctionality, and scalable manufacturing for practical structural color applications.

### 2.3. Enhancing Structural Color Visibility and Saturation Through Absorption Management

Although structural colors originate primarily from interference, diffraction, scattering, or resonance, the generation of a wavelength-selective optical response does not necessarily guarantee a vivid color perceived by the human eye. This issue is particularly important in colloidal PCs, photonic glasses, and other particle-based structures, where light can undergo repeated scattering before leaving the material. In addition to the desired wavelength-selective reflection or coherent scattering, these multiple-scattering processes generate a broadband optical background across the visible spectrum. When the broadband component becomes comparable to the wavelength-selective signal, the reflected light contains substantial contributions from different wavelengths and the structural color consequently appears pale, whitish, or desaturated. Therefore, suppressing undesired broadband scattering while preserving the desired structural reflection is an important strategy for improving the visibility and saturation of structural colors [150].

One of the most widely used approaches is the introduction of broadband absorbing materials, particularly carbon-based black absorbers. The absorber preferentially attenuates light that propagates over long and complex paths through the structure and undergoes multiple scattering, while a substantial fraction of the selectively reflected light generated near the surface can still escape from the material. Consequently, the broadband background is reduced and the spectral contrast of the structural color is increased. Colloidal PC pigments that appeared white developed well-defined structural colors after being deposited on a black light-absorbing background [170]. Similarly, the addition of a small amount of carbon black to Polystyrene nanoparticles (PSNPs) colloidal assemblies was shown to substantially suppress incoherent multiple scattering while only slightly reducing the intensity of the wavelength-selective reflection peak [171]. Demirörs et al. incorporated carbon black into silica-based photonic colloidal glass inks for 3D printing. Increasing the carbon black concentration from 0.3 to 1.4 wt% reduced the reflectance at the green structural-color peak from approximately 47% to 28% without significantly changing the peak position or bandwidth, demonstrating that broadband absorption can suppress multiple-scattering contributions without altering the characteristic structural wavelength [172].

Another important strategy is to incorporate absorption directly into the structural building blocks rather than adding a separate black absorber. Melanin provides an important biological model because it combines a relatively high refractive index with broadband absorption in the visible region. Inspired by natural melanosomes, Polydopamine (PDA), a synthetic melanin-like material, has been widely employed to fabricate absorption-assisted structural color materials. Kohri et al. assembled black PDA particles into amorphous structures and obtained bright non-iridescent structural colors without additional absorbing additives [173]. In hybrid systems, PDA can also be introduced as an absorbing coating or secondary component. For example, silica particles coated or co-assembled with PDA have been used to suppress unwanted scattering and increase the saturation of non-iridescent structural colors while simultaneously improving particle adhesion and mechanical stability [174].

Carbon-based nanomaterials can provide similar absorption control while introducing additional optical functionalities. Zhang et al. incorporated graphene nanosheets containing graphene quantum dots into short-range ordered amorphous photonic structures. The broadband absorption of the graphene nanosheets suppressed unwanted visible-light contributions and increased color saturation, while the photoluminescence of the graphene quantum dots contributed to maintaining high brightness, thereby addressing the common trade-off between saturation and brightness [175]. Absorption can also be incorporated through core–shell or hybrid particle designs. For example, carbon-coated dielectric particles can suppress diffuse multiple scattering, whereas metal-dielectric core–shell particles can selectively absorb unwanted spectral components while retaining the desired structural reflection. Such approaches are particularly useful for long-wavelength structural colors, for which short-wavelength scattering often produces a strong blue background and substantially decreases red color purity [150,151]. Overall, absorption management provides a general strategy for improving structural color visibility without changing the fundamental mechanism responsible for wavelength selection. Black absorbers, melanin-like materials, graphene-based components, and absorbing core–shell structures can reduce unwanted broadband scattering and increase the spectral contrast between the desired structural-color signal and its optical background.

## 3. Quantification Methods of Structural Colors

The previous sections have focused on the physical mechanisms responsible for structural color generation and the nanostructures used to manipulate light. However, understanding how structural colors are generated is only one part of structural color research. Equally important is the ability to quantitatively evaluate their optical performance using standardized and reproducible metrics. As structural colors continue to move from laboratory demonstrations toward practical applications in manufacturing, sensing, displays, and security technologies, quantitative characterization has become essential for objectively comparing different material systems, fabrication strategies, and device performance. This shift from qualitative description to quantitative evaluation has become a key trend in modern structural color research. Accordingly, the following section reviews the principal methods used to quantitatively evaluate structural colors, including optical spectroscopy, colorimetry, angular response, spatial resolution, and structural order.

### 3.1. Optical Spectroscopy

Reflectance, transmittance, and absorptance spectra provide the most fundamental metrics for quantitatively evaluating structural colors because the perceived color originates directly from wavelength-selective reflection, transmission, and absorption. Unlike conventional pigments, whose optical response mainly depends on molecular absorption, structural colors are governed by interference, diffraction, scattering, or resonance. Consequently, nearly every optical characteristic of a structural color can be quantitatively extracted from its reflectance, transmittance, or absorptance spectrum. These spectra not only determine the displayed color but also reveal the optical quality, resonance behavior, fabrication accuracy, and environmental response of the underlying nanostructure. It should be emphasized that the applicability of individual spectral parameters depends strongly on the spectral line shape and the underlying coloration mechanism. Peak-based metrics, such as peak intensity or full width at half maximum (FWHM) are most directly applicable to spectra containing an isolated and well-defined resonance. For multimode spectra, these parameters can be applied to individual modes only when the resonances are sufficiently separated; otherwise, spectral decomposition or line-shape fitting is required. For asymmetric resonances, fitted line-shape parameters, such as the Fano asymmetry parameter and resonance linewidth, provide a more appropriate description than a conventional FWHM alone. In broadband or diffuse-scattering systems, where a distinct resonance peak may not exist, integrated spectral quantities, spectral centroid, diffuse reflectance, and colorimetric parameters are more meaningful. Similarly, for absorption-assisted structural colors, reflectance should be interpreted together with transmittance and absorptance because background suppression may originate from intentional material absorption rather than enhanced resonance selectivity. Therefore, the spectral metrics discussed below should be regarded as complementary parameters selected according to the characteristics of the measured spectrum rather than as universally applicable descriptors.

#### 3.1.1. Peak Wavelength (λ_peak_)

**Figure 13:**
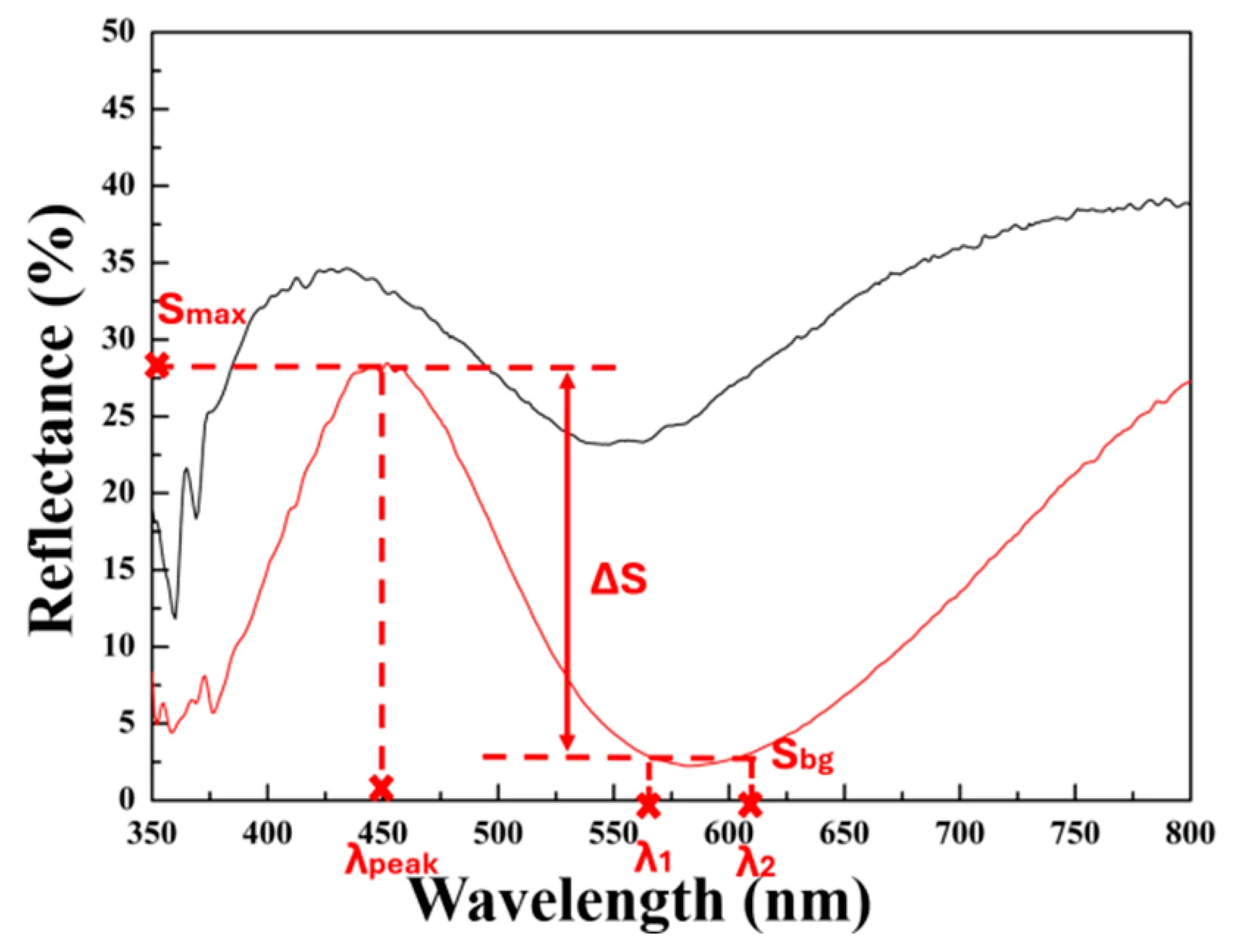
Schematic representation of reflection spectra and definitions of spectral quantification metrics. (The dashed lines are auxiliary guide lines).

Peak wavelength (λ_peak_) is the most fundamental parameter for quantitatively characterizing structural colors because it directly determines the perceived color of a material. In reflectance spectra, λ_peak_ is defined as the wavelength corresponding to the maximum reflectance (as shown in Figure 13), whereas in transmittance spectra it represents the wavelength of maximum transmission. Since structural colors originate from wavelength-selective optical interactions rather than chemical absorption, the peak wavelength serves as the primary indicator of the optical response regardless of whether the coloration mechanism is based on thin-film interference, photonic crystals, diffraction gratings, LSPR or metasurfaces. The peak wavelength is closely associated with the characteristic structural dimensions of the optical nanostructure. For example, in thin-film interference, λ_peak_ is primarily governed by the optical thickness of the dielectric layer. In photonic crystals, it is determined by the lattice spacing and effective refractive index according to Bragg diffraction, while in plasmonic and dielectric nanostructures it is dictated by the resonant dimensions and geometry of the individual nanoresonators. Consequently, measuring λ_peak_ provides a direct means of correlating optical properties with structural parameters and evaluating fabrication accuracy.

Because wavelength can be measured with nanometer-level precision, λ_peak_ provides a highly reproducible and objective metric that is independent of illumination intensity, making it considerably more reliable than visual color observation. Although λ_peak_ determines the dominant reflected or transmitted color, it alone cannot fully describe the optical quality of structural colors. Parameters such as peak intensity, spectral bandwidth, background reflectance, and peak symmetry also influence color brightness, saturation, and spectral selectivity. Therefore, peak wavelength should be considered together with these complementary spectral metrics to achieve a comprehensive quantitative evaluation of structural color performance.

#### 3.1.2. Peak Intensity (S_max_)

While the peak wavelength determines the dominant color produced by a structural color system, the peak intensity (S_max_) describes the strength of the optical response at that wavelength, as shown in Figure 13. In reflectance spectra, the peak intensity corresponds to the maximum reflectance (R_max_), whereas in transmittance spectra it represents the maximum transmittance (T_max_). Because structural colors arise from wavelength-selective optical interactions rather than broadband reflection, the peak intensity directly reflects the efficiency with which a nanostructure selectively reflects or transmits light at the resonant wavelength. Peak intensity is strongly influenced by both the optical design and the structural quality of the nanostructure. Factors such as refractive-index contrast, resonance strength, material absorption, surface roughness, fabrication defects, and structural disorder all affect the maximum reflected or transmitted intensity. For example, photonic crystals with a large refractive-index contrast generally exhibit stronger Bragg reflection and therefore higher reflectance peaks, whereas plasmonic nanostructures often suffer from ohmic losses in metals, which can reduce the reflected or transmitted intensity despite exhibiting narrow resonance features. Similarly, fabrication imperfections and structural non-uniformity increase optical scattering and reduce the peak intensity, making this parameter a useful indicator of fabrication quality. Peak intensity also plays an important role in determining the visual appearance of structural colors. Higher reflectance generally produces brighter and more vivid colors, whereas weak spectral peaks often result in dull or desaturated appearances even if the peak wavelength remains unchanged. However, peak intensity alone does not determine color purity because a high background reflectance may still reduce visual contrast. Consequently, peak intensity should be interpreted together with background reflectance and spectral bandwidth to provide a comprehensive assessment of color quality.

In dynamic structural color systems, variations in peak intensity are frequently used to quantify changes in optical modulation. External stimuli such as mechanical deformation, refractive-index variation, molecular adsorption, or electrical tuning may alter the resonance strength without producing a significant wavelength shift. Therefore, monitoring changes in peak intensity provides complementary information to peak wavelength and is particularly useful for sensing applications and actively tunable optical devices.

#### 3.1.3. Spectral Selectivity

Although the peak wavelength and peak intensity determine the dominant color and brightness of a structural color, they do not fully describe its spectral quality. In practical optical systems, light outside the resonance wavelength is rarely completely suppressed. Residual reflection or transmission from non-resonant wavelengths forms the spectral background, which significantly influences color purity, visual contrast, and resonance selectivity. Consequently, the evaluation of structural colors requires not only the characterization of the resonance peak itself but also quantitative assessment of the spectral background and the sharpness of the resonance.

The spectral background generally originates from non-resonant optical processes, including Fresnel reflection, diffuse scattering caused by structural disorder or surface roughness, multiple optical modes, and material absorption. Depending on the coloration mechanism, the background may appear as a nearly constant broadband response or as slowly varying spectral features. A low spectral background indicates that wavelengths outside the desired resonance are effectively suppressed, resulting in higher color saturation and better spectral selectivity. Conversely, an elevated background broadens the color spectrum, leading to reduced chromatic purity even when the resonance wavelength remains unchanged.

One of the simplest approaches is to calculate the average background over a selected wavelength range while excluding the resonance region, and the average background (${\bar{S}}_{bg}$) can be described by weighted average formula as Equation (10) [176].

(10) $${\bar{S}}_{bg}=\frac{1}{\lambda_{2}-\lambda_{1}}\int_{\lambda_{1}}^{\lambda_{2}}S(\lambda )d\lambda$$

where S(λ) denotes the measured spectral response, which may represent reflectance, transmittance, absorbance, or other wavelength-dependent optical quantities according to the characterization method employed, and the resonance region is excluded from the integration. $\lambda_{1}$ and $\lambda_{2}$ are the beginning and the end of selected wavelength range, as shown in Figure 13 [176]. In the broader field of optics, the peak-to-background ratio (PBR) is a standard metric used to describe resonance prominence, which can be expressed by the ratio of the peak intensity ($S_{max}$) and the average background (${\bar{S}}_{bg}$) as Equation (11) [177].

(11) $$PBR=\frac{S_{max}}{{\bar{S}}_{bg}}$$

where $S_{max}$ and ${\bar{S}}_{bg}$ can be applied to reflectance, transmittance, or absorptance. When the ${\bar{S}}_{bg}$ in every case is about the same, a larger ratio indicates that the desired optical mode dominates the spectrum, producing a visually cleaner structural color [178]. However, when narrowing the focus to the structural color field, PBR is seldom utilized as a quantitative indicator. Although such ratio-based metrics are occasionally adopted, they fail to preserve the absolute optical modulation amplitude. Because the perceived brightness and saturation of structural colors are strictly dictated by the absolute spectral response, the peak-to-background difference provides a far more physically meaningful measure of resonance strength.

Therefore, in addition to ratio-based metrics, the absolute peak-to-background difference provides a more direct measure of the spectral modulation. This parameter is defined as the difference between the peak spectral response and the average off-resonance background (∆S), as expressed in Equation (12) [169]:

(12) $$∆S=S_{max}-{\bar{S}}_{bg}$$

Compared with peak-to-background ratios, this difference-based metric (∆S) better preserves the absolute modulation amplitude, which is particularly important for structural colors because visual brightness and chromatic contrast are strongly affected by the magnitude of reflected or transmitted light.

Besides the spectral background, the peak width of the spectra is another essential parameter describing spectral selectivity. The FWHM is defined as the spectral width measured at one-half of the peak intensity. Narrow FWHM values indicate highly selective wavelength filtering and are generally associated with high color purity, strong resonance confinement, and reduced spectral overlap between neighboring colors [179]. In contrast, broad resonances produce wider reflection bands, resulting in lower color saturation even when the peak wavelength remains unchanged. FWHM is widely used to compare the optical performance of different structural color platforms. Plasmonic resonances often exhibit broader linewidths because of intrinsic metallic losses [180], whereas dielectric metasurfaces [181], photonic crystals [182], and high-quality Fabry-Pérot resonators [183] are capable of producing considerably narrower resonances. Consequently, FWHM serves as an important indicator of resonance quality and fabrication precision. For resonant structural color systems, the resonance quality is sometimes represented by the quality factor (Q), although its exact definition depends on the resonance mechanism [184,185]. For simple single-mode resonators, Q is often approximated by the ratio between the resonance wavelength and its linewidth, whereas more rigorous definitions are based on the ratio of stored electromagnetic energy to energy dissipation per optical cycle. Consequently, Q is mainly employed for resonant photonic structures rather than being a universal metric for structural colors.

#### 3.1.4. Peak Symmetry

Besides the resonance wavelength and linewidth, the spectral line shape provides additional information regarding the underlying optical mechanism. An ideal resonance generated by a single optical mode generally exhibits a symmetric spectral profile, whereas interactions between multiple optical modes or structural inhomogeneity often produce asymmetric or distorted resonance peaks. Therefore, peak symmetry has become an important qualitative and quantitative parameter for identifying resonance mechanisms and evaluating structural quality. For an isolated resonance dominated by a single optical mode, the spectral response is often well approximated by a symmetric Lorentzian line shape. Such behavior is commonly observed in single-mode resonant systems including Fabry-Pérot cavities, Bragg resonances in photonic crystals, and Mie resonances of dielectric nanostructures when interference with additional optical pathways is negligible. However, many practical structural color systems involve multiple optical pathways or coupled resonances. When a narrow resonant mode interferes with a broadband continuum, the spectral profile evolves into an asymmetric Fano line shape. One of the most representative asymmetric spectral profiles is the Fano resonance, which originates from the interference between a broadband continuum state and a narrow discrete resonance. Unlike the symmetric Lorentzian line shape, Fano resonances exhibit a characteristic asymmetric peak–dip profile with one side rising gradually and the other dropping sharply. This unique spectral feature has attracted considerable interest because the steep spectral slope greatly enhances wavelength discrimination and optical sensitivity, making Fano resonances particularly attractive for structural color sensing, optical filtering, and high-resolution spectroscopy.

The degree of spectral asymmetry can be quantitatively described using the Fano line-shape equation, expressed as Equations (13) and (14) [186]:

(13) $$F(\varepsilon )=I_{0}\frac{{\left(q+\varepsilon \right)}^{2}}{1+\varepsilon^{2}}$$

(14) $$\varepsilon =\frac{\lambda -\lambda_{0}}{\Gamma /2}$$

where $I_{0}$ is resonance intensity, λ represents the angular wavelength of incident light, $\lambda_{0}$ is the resonance wavelength, Γ is the linewidth, and q is the Fano asymmetry parameter. The Fano asymmetry parameter q determines the resonance line shape. As |q|→∞, the spectrum approaches a symmetric Lorentzian peak. In contrast, q = 0 produces an inverted Lorentzian (anti-resonance) characterized by a symmetric spectral dip. Intermediate values of q give rise to the characteristic asymmetric Fano profile. Consequently, the Fano parameter has become a standard quantitative metric for evaluating asymmetric resonances in plasmonic nanostructures, dielectric metasurfaces, and guided-mode resonance devices. It should be noted that spectral asymmetry is not exclusively associated with Fano resonance [187]. Structural disorder, overlapping resonance modes, polarization coupling, and fabrication non-uniformity may also distort the spectral profile [188]. Therefore, peak symmetry should be interpreted together with other spectral parameters, including the peak wavelength, peak intensity, and linewidth, to provide a comprehensive evaluation of the optical characteristics of structural color systems.

#### 3.1.5. Peak Shift Sensitivity

Peak shift sensitivity is one of the most important quantitative parameters for evaluating dynamic structural color systems because it describes how effectively external stimuli are converted into measurable spectral changes. Unlike static structural colors, whose optical performance is primarily characterized by the resonance wavelength and spectral profile, tunable structural colors rely on changes in the resonance condition induced by environmental or physical perturbations. Such perturbations modify the effective refractive index, lattice spacing, optical path length, or resonator geometry, resulting in a shift in the resonance wavelength that can be accurately monitored using optical spectroscopy.

The peak shift is generally expressed as Equation (15) [189].

(15) $$∆\lambda =\lambda -\lambda_{0}$$

where $\lambda_{0}$ and λ represent the resonance wavelengths before and after the external stimulus, respectively. A positive wavelength shift corresponds to a redshift, whereas a negative shift indicates a blueshift. Since modern spectrometers can routinely resolve wavelength variations of less than one nanometer, peak-shift measurements provide a highly reproducible and objective method for quantifying optical responses. In addition, to compare the performance of different structural color platforms, the peak shift is normalized by the magnitude of the applied stimulus to obtain the sensitivity (*S*), as expressed in Equation (16) [189].

(16) $$S=\frac{∆\lambda }{∆X}$$

where X denotes the physical quantity being measured. The unit of sensitivity depends on the sensing mechanism.

Although sensitivity is the most frequently reported performance metric, it does not completely describe sensing capability. A large wavelength shift is beneficial only when the resonance peak remains sufficiently narrow to allow accurate wavelength determination. Therefore, the figure of merit (FOM) is often employed to simultaneously account for wavelength sensitivity and spectral linewidth. FOM can be expressed as Equation (17) [190]

(17) $$FOM=\frac{S}{FWHM}$$

A higher FOM indicates that a small variation in the measured parameter produces a readily distinguishable wavelength shift, making the sensor more suitable for high-resolution detection. The minimum detectable wavelength shift is further determined by the spectral resolution of the measurement system and the signal-to-noise ratio. Consequently, the practical detection limit depends not only on the intrinsic sensitivity of the structural color device but also on the linewidth, resonance stability, and instrumental performance. These factors should therefore be considered collectively when comparing the sensing performance of different structural color platforms. Peak shift sensitivity has become the standard quantitative metric for dynamic structural colors based on thin-film interference, photonic crystals, plasmonic nanostructures, dielectric metasurfaces, and inverse opal structures. Because wavelength can be measured with high precision and is largely independent of illumination intensity, peak-shift analysis provides a universal and robust strategy for quantifying optical responses across a broad range of structural color sensing applications.

Collectively, the spectral metrics discussed above (including the peak wavelength, peak intensity, spectral selectivity, peak symmetry, and peak shift sensitivity) provide a comprehensive quantitative framework for evaluating the optical characteristics of structural colors. These parameters establish the relationship between the measured optical spectrum and the underlying nanostructure, enabling objective comparisons of resonance behavior, fabrication quality, and dynamic optical responses. However, although spectral analysis accurately describes wavelength-dependent optical properties, it does not directly represent how colors are perceived by the human visual system. Since the same spectral profile may produce different visual impressions under different illumination conditions or observer responses, a perceptually standardized color representation is required. Consequently, the Commission Internationale de l’éclairage (CIE) 1931 chromaticity diagram and the perceptually uniform CIE 1976 color space have become the most widely adopted standards for quantitatively evaluating structural colors and objectively comparing their chromatic performance.

### 3.2. Colorimetry and Color Spaces

Colorimetry provides the theoretical framework for quantitatively describing color based on human visual perception, whereas a color space serves as the mathematical representation that maps perceived colors into standardized coordinates. By transforming measured spectral information into numerical color coordinates, color spaces enable objective comparison of color appearance independent of subjective visual judgment. Among the numerous color spaces developed for color characterization, the CIE 1931 chromaticity diagram and the CIE 1976 color spaces remain the two most influential standards. The CIE 1931 system establishes the fundamental relationship between optical spectra and perceived chromaticity, while the CIE 1976 color spaces introduce perceptually uniform coordinates that enable more accurate quantification of color differences. Accordingly, the following sections introduce the principles of the CIE 1931 chromaticity diagram and the CIE 1976 color spaces, together with their applications in the quantitative evaluation of structural colors.

#### 3.2.1. CIE 1931

**Figure 14:**
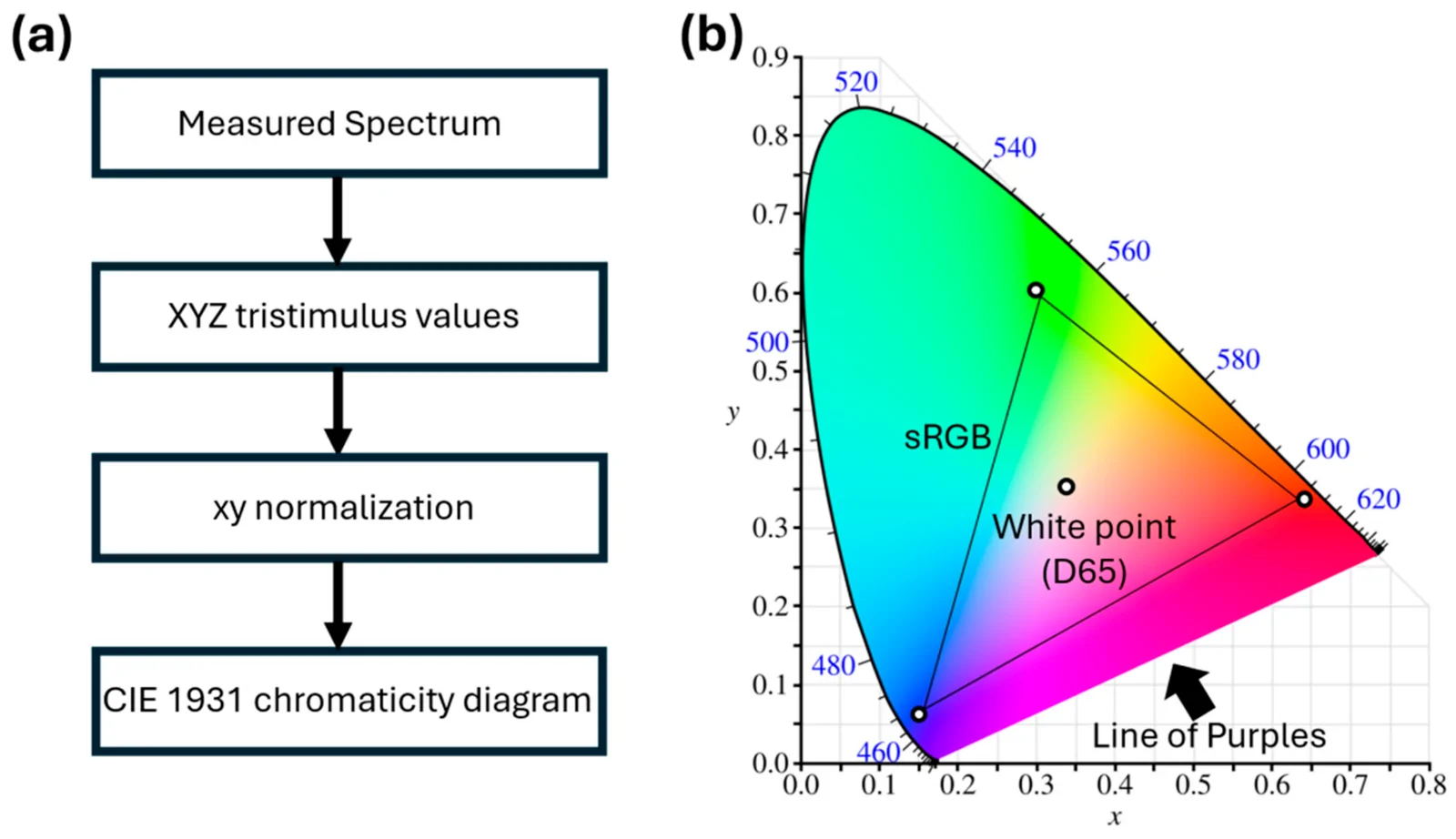
(**a**) Schematic illustration of the CIE 1931 colorimetric processing. (**b**) CIE 1931 chromaticity diagram.

Although an optical spectrum completely describes the wavelength-dependent interaction between light and a structural color, it cannot directly represent the color perceived by the human eye. Human color vision is based on the combined responses of three types of cone photoreceptors, each exhibiting different spectral sensitivities across the visible region. Consequently, multiple spectral distributions may produce identical visual sensations, a phenomenon known as metamerism, while identical spectra may appear differently under different illumination conditions or viewing environments. Therefore, an objective colorimetric framework is required to transform measured spectral information into standardized numerical coordinates that correspond to human color perception. To establish a universal standard for color representation, CIE introduced the CIE 1931 colorimetric system, which forms the foundation of modern color science. As shown in Figure 14a, rather than describing colors directly by their spectral distributions, the CIE 1931 system converts spectral information into three tristimulus values, X, Y, and Z, using standardized color-matching functions derived from the average responses of human observers [8]. These tristimulus values provide a device-independent and observer-standardized description of color, enabling quantitative comparison of optical properties among different structural color systems. The XYZ tristimulus values are subsequently normalized to obtain the chromaticity coordinates x and y, which eliminate the influence of overall light intensity while preserving chromatic information [8]. These coordinates are then represented on the well-known CIE 1931 chromaticity diagram, commonly referred to as the horseshoe diagram, providing an intuitive visualization of the relationship between spectral characteristics and perceived color, as shown in Figure 14b. As a result, the CIE 1931 chromaticity diagram has become the most widely adopted tool for evaluating the chromatic performance, color gamut, and color purity of structural colors.

The CIE 1931 colorimetric system is based on the principle of trichromatic vision, which states that any perceivable color can be reproduced by an appropriate combination of three primary stimuli. Instead of employing physical red, green, and blue primaries, the CIE defined three hypothetical primaries, X, Y, and Z, to ensure that all visible colors could be represented using positive tristimulus values. The tristimulus values are obtained by integrating the measured spectral power distribution with the standardized CIE 1931 color-matching functions, which describe the average spectral responses of a standard human observer under specified illumination conditions. Among the three tristimulus values, Y is proportional to the perceived luminance, whereas X and Z primarily contribute to chromatic information [8]. Although the XYZ tristimulus values provide a complete numerical description of color, they contain both chromaticity and luminance information. To describe color independent of brightness, the tristimulus values are normalized as x, y, and z to obtain the chromaticity coordinates. Because the three normalized coordinates satisfy x + y + z = 1, only the x and y coordinates are required to uniquely specify the chromaticity of a color [8]. Plotting these coordinates yields the CIE 1931 chromaticity diagram, which provides a two-dimensional representation of human color perception independent of luminance, as shown in Figure 14b.

The outer boundary of the chromaticity diagram, known as the spectral locus, consists of monochromatic colors corresponding to single wavelengths across the visible spectrum. Colors located near the spectral locus exhibit high spectral purity and saturation because they originate from narrowband optical responses. In contrast, colors positioned toward the center of the diagram contain broader spectral distributions or multiple wavelength components, resulting in lower saturation and a more whitish appearance. The lower straight boundary connecting the two ends of the spectral locus is referred to as the line of purples. Unlike the spectral locus, the colors along this boundary do not correspond to any single wavelength in the visible spectrum but instead arise from the simultaneous stimulation of the short- and long-wavelength cone photoreceptors. Consequently, these nonspectral colors are perceptual constructs generated by the human visual system rather than by monochromatic light. For structural colors, the chromaticity diagram provides a convenient and intuitive method for evaluating color performance. Structural colors exhibiting narrow resonance peaks and low spectral background generally produce chromaticity coordinates located closer to the spectral locus, indicating high color purity and saturation. Conversely, broadband reflection, multiple resonance modes, or elevated background scattering shift the chromaticity coordinates toward the white point, leading to desaturated or muddy colors. Therefore, the CIE 1931 chromaticity diagram has become one of the most widely adopted tools for comparing the color gamut, chromaticity distribution, and color purity of different structural color systems [191].

Beyond representing individual colors, the CIE 1931 chromaticity diagram also provides a quantitative framework for evaluating the color gamut, which refers to the range of reproducible colors achievable by a structural color system [191]. In the chromaticity diagram, the color gamut is typically represented by a polygon connecting the chromaticity coordinates of the primary colors. A larger enclosed area generally indicates a broader range of reproducible colors and a higher capability for full-color reproduction. Consequently, color gamut has become one of the most important quantitative metrics for assessing structural colors intended for applications such as displays, color printing, optical encryption, and decorative coatings. To enable objective comparison among different optical systems, the color gamut is commonly expressed relative to a standard color space, such as sRGB, Adobe RGB, or Rec. 2020. Among these standards, sRGB is the most widely adopted reference because it represents the default color space for most consumer electronic devices and digital imaging systems, as shown in Figure 14b. The color gamut coverage is generally expressed as Equation (18) [192].

(18) $$Color\;Gamut\;Coverage\left(\%\right)=\frac{A_{sample}}{A_{reference}}$$

where $A_{sample}$ is the chromaticity area enclosed by the structural color system and $A_{reference}$ is the area of the selected reference color space. Consequently, values exceeding 100% indicate that the achievable color gamut extends beyond the selected reference standard [191]. For example, structural color systems based on plasmonic nanostructures and dielectric metasurfaces have reported color gamut coverages exceeding 168.2% of the sRGB color space, demonstrating their capability to generate highly saturated colors [191]. It should be noted, however, that color gamut coverage alone does not fully characterize color performance. Because the metric is based primarily on the enclosed chromaticity area, two structural color systems with identical gamut coverage may exhibit substantially different color distributions, spectral purity, or coverage of specific color regions. Therefore, color gamut should be interpreted together with chromaticity coordinates, spectral characteristics, and perceptual color difference metrics to provide a comprehensive evaluation of structural color performance. More recently, gamut overlap and color volume have also been introduced to provide a more comprehensive evaluation of color reproduction. For instance, Mohamed ElKabbash et al. demonstrated a structural color platform capable of achieving nearly complete (>99%) coverage of both the sRGB and Adobe RGB color spaces. Furthermore, the enclosed chromaticity area reached 177.5% and 131.6% of the corresponding sRGB and Adobe RGB color gamuts, respectively [8]. This example highlights the distinction between gamut coverage and gamut area: while gamut coverage quantifies how completely a structural color system reproduces the colors within a standard color space, gamut area compares the overall extent of the achievable color gamut with that of the reference standard. Together, these complementary metrics provide a more comprehensive evaluation of color reproduction capability than either metric alone.

#### 3.2.2. CIE 1976

Although the CIE 1931 chromaticity diagram provides a standardized framework for representing perceived colors, it does not constitute a perceptually uniform color space. Equal geometric distances between two points on the chromaticity diagram do not necessarily correspond to equal visual color differences perceived by the human eye. Consequently, two colors separated by the same Euclidean distance in different regions of the CIE 1931 chromaticity diagram may appear significantly different in one case but nearly indistinguishable in another. This non-uniformity limits the applicability of the CIE 1931 system for quantitatively evaluating color differences, color stability, and fabrication accuracy. To overcome this limitation, the CIE introduced the CIE 1976 color spaces, which were specifically designed to improve perceptual uniformity [193]. Unlike the CIE 1931 chromaticity diagram, the CIE 1976 system was developed so that equal distances within the color space more closely correspond to equal perceived color differences. Among the CIE 1976 color spaces, CIELAB (L*a*b*) has become the most widely adopted standard for quantitative color evaluation in structural color research because of its intuitive representation and compatibility with color difference analysis [194]. Unlike the chromaticity coordinates in the CIE 1931 system, the CIELAB coordinates are obtained through a nonlinear transformation of the CIE 1931 XYZ tristimulus values. The CIELAB color space consists of three orthogonal coordinates. L* represents the perceptual lightness, ranging from 0 (black) to 100 (white). a* represents the red-green opponent axis, with positive values corresponding to red and negative values corresponding to green. b* represents the yellow-blue opponent axis, with positive values corresponding to yellow and negative values corresponding to blue [194]. Unlike the CIE 1931 chromaticity coordinates, which describe only chromaticity independent of luminance, the CIELAB color space explicitly separates lightness (L*) from chromatic components (a* and b*), enabling simultaneous evaluation of both brightness and color appearance.

**Figure 15:**
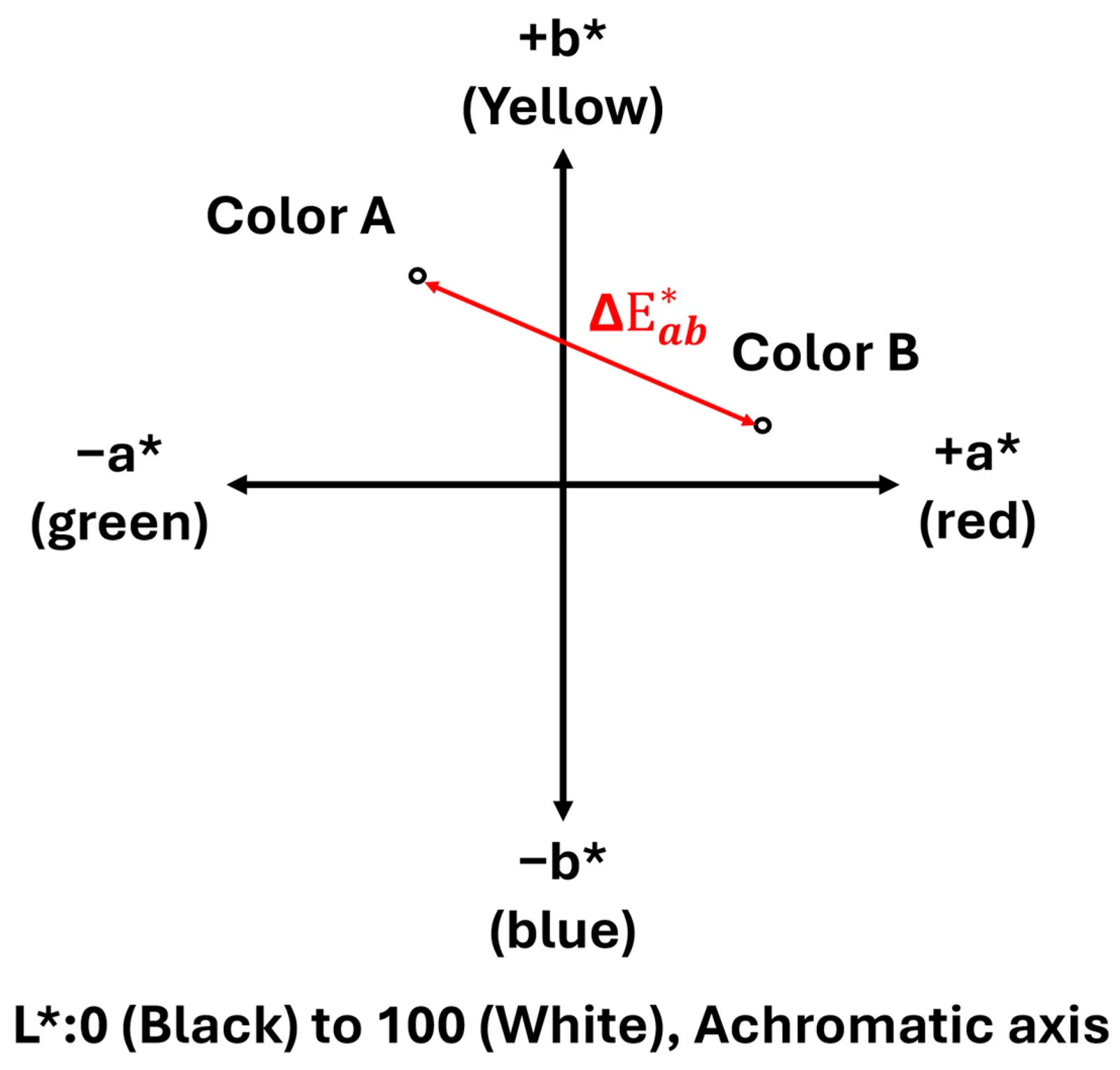
Schematic illustration of the CIE 1976 CIELAB color space and color difference (ΔE^*^_ab_).

By separating luminance from chromatic information, CIELAB provides a perceptually more uniform representation of color appearance, allowing meaningful numerical comparison between different colors. One of the greatest advantages of the CIELAB color space is that the perceptual difference between two colors can be quantified by a single numerical parameter (ΔE^*^_ab_). As shown in Figure 15, the original CIE 1976 color difference (ΔE^*^_ab_) is defined as the Euclidean distance between two points in the CIELAB color space, expressed as Equation (19) [194].

(19) $$∆E_{ab}^{*}=\sqrt{{∆L}^{*2}+{∆a}^{*2}+{∆b}^{*2}}$$

where ${∆L}^{*}$, ${∆a}^{*}$, and ${∆b}^{*}$ denote the differences in lightness, the red-green coordinate, and the yellow-blue coordinate, respectively. Because $∆E_{ab}^{*}$ represents the perceptual distance between two colors, smaller values indicate higher color similarity, whereas larger values correspond to increasingly distinguishable visual differences.

For structural color systems, ΔE has become one of the most widely adopted quantitative metrics for evaluating color performance. It is commonly used to assess fabrication repeatability, environmental stability, durability under long-term operation, agreement between numerical simulations and experimental measurements, and the optical response of dynamically tunable structural colors. Unlike chromaticity coordinates, which describe the absolute position of a color within the chromaticity diagram, ΔE directly quantifies the perceptual difference between two colors, making it particularly suitable for evaluating color consistency and device reliability [195,196]. Although the original CIE 1976 color difference, $∆E_{ab}^{*}$, provides a simple Euclidean measure of color separation in the CIELAB color space, perceptual uniformity is not perfectly maintained throughout the entire color space. To improve the agreement between calculated and perceived color differences, several modified color-difference formulas have subsequently been developed, among which CIEDE2000 is now widely used. The CIEDE2000 color difference is expressed as Equation (20).

(20) $$∆E_{00}^{*}=\sqrt{{\left(\frac{{\Delta L}^{\prime }}{K_{L}S_{L}}\right)}^{2}+{\left(\frac{{\Delta C}^{\prime }}{K_{C}S_{C}}\right)}^{2}+{\left(\frac{{\Delta H}^{\prime }}{K_{H}S_{H}}\right)}^{2}+R_{T}(\frac{{\Delta C}^{\prime }}{K_{C}S_{C}})(\frac{{\Delta H}^{\prime }}{K_{H}S_{H}})}$$

where ${\Delta L}^{\prime }$, ${\Delta C}^{\prime }$, and ${\Delta H}^{\prime }$ represent the corrected differences in lightness, chroma, and hue, respectively; $S_{L}$, $S_{c}$, and $S_{H}$ are weighting functions; $K_{L}$, $K_{C}$, and $K_{H}$ are parametric factors; and $R_{T}$ is the interaction between chroma and hue differences [197].

CIEDE2000 retains the CIELAB coordinates as the fundamental color representation but introduces corrections for differences in lightness, chroma, and hue, together with weighting and interaction terms. The resulting color difference is expressed as ΔE^*^_00_, with smaller values indicating greater perceptual similarity between two colors [198]. This metric is particularly useful for evaluating small color variations caused by fabrication deviations, environmental changes, or changes in illumination and observation geometry.

### 3.3. Quantitative Characterization of Angular Response

#### 3.3.1. Angular Peak Wavelength Shift

The angular dependence of the peak wavelength provides a direct and widely used approach for quantitatively evaluating the angular stability of structural colors. Because the perceived color of a structural color system is strongly related to its characteristic spectral wavelength, changes in the illumination or observation angle can be quantified by tracking the corresponding shift in the reflection, transmission, or absorption peak. The angular peak wavelength shift (∆λ(θ)) can be expressed as Equation (21).

(21) $$∆\lambda \left(\theta \right)=\lambda \left(\theta \right)-\lambda \left(\theta_{0}\right)$$

where λ(θ) is the peak wavelength measured at angle θ, and $\theta_{0}$ is a reference angle, typically normal incidence or another specified measurement geometry. A larger |∆λ(θ)| over a given angular range indicates stronger angular dependence, whereas a smaller wavelength shift represents greater angular stability [138]. In addition to the absolute wavelength shift, the relative wavelength shift has also been employed to facilitate comparison between structural colors located at different spectral regions. The relative wavelength shift can be expressed as Equation (22) [197].

(22) $$∆\lambda_{R}\left(\theta \right)=\frac{∆\lambda \left(\theta \right)}{\lambda \left(\theta_{0}\right)}$$

For example, Shrestha et al. reported a measured wavelength variation of only approximately 1.8% as the incidence angle increased from 0° to 80°, demonstrating a highly angle-insensitive structural color filter [199]. In addition, the wavelength variation can also be normalized by the corresponding angular change to describe the rate of spectral shift as expressed as Equation (23) [199,200].

(23) $$S_{\theta }=\frac{∆\lambda \left(\theta \right)}{∆\theta }$$

where $S_{\theta }$ represents the wavelength shift per unit angular change, expressed in nm/degree. These studies demonstrate that angular peak wavelength shift (∆λ(θ)), its normalized relative shift, and the wavelength-shift rate provide practical quantitative measures for comparing the angular spectral stability of different structural color systems. However, because the reported angular ranges, measurement geometries, and characteristic spectral features differ among studies, these parameters should be compared under clearly specified experimental conditions.

#### 3.3.2. Angular Color Difference

Although the angular shift in a characteristic spectral wavelength provides a simple and direct measure of spectral stability, wavelength variation alone does not fully describe the corresponding change in perceived color. The same spectral shift may produce different perceptual color changes depending on the initial wavelength, spectral bandwidth, and spectral profile. For instance, the human eye is particularly sensitive to green light (peak wavelength ~555 nm). Consequently, even minor spectral shifts in the green region are far more noticeable to observers than similar shifts in the blue or red regions [54]. Therefore, color difference provides a complementary approach for quantitatively evaluating the angular stability of structural colors from the perspective of human color perception. Based on the CIEDE2000 color-difference metric introduced in Section 3.2.2, the angular color difference can be expressed as Equation (24).

(24) $$∆E_{00}^{*}(\theta )=∆E_{00}^{*}[C\left(\theta \right),C\left(\theta_{0}\right)]$$

where C(θ) and $C\left(\theta_{0}\right)$ represent the CIELAB color coordinates obtained at angle θ and a reference angle $\theta_{0}$, respectively. The reference angle is commonly selected as normal incidence or another specified illumination-observation geometry. A smaller $∆E_{00}^{*}(\theta )$ indicates that the perceived color remains closer to the reference color and therefore corresponds to greater angular color stability, whereas a larger value indicates a more pronounced angular color variation.

As summarized in Table 3, no universally accepted quantitative definition or threshold has yet been established for distinguishing non-iridescent from iridescent structural colors. In many previous studies, angular stability has been evaluated qualitatively by comparing optical images or angle-resolved spectra [154,201,202,203,204], whereas only a limited number of studies have introduced explicit quantitative parameters [138,199,205,206,207]. Nevertheless, the quantitative results reported in the literature provide useful benchmarks for comparing the angular stability of different structural color systems. For example, structural colors regarded as having high angular stability have been reported to exhibit an absolute peak wavelength shift (|∆λ(θ)|) of less than approximately 15 nm over the investigated angular range, a CIEDE2000 color difference ($∆E_{00}^{*}(\theta )$), below approximately 8, or a relative peak wavelength shift (($∆\lambda \left(\theta \right)/\lambda \left(\theta_{0}\right)$), below approximately 2%. These parameters can be used as quantitative criteria for identifying non-iridescent structural colors over the specified angular range. Structural colors satisfying the applicable criterion exhibit sufficiently small spectral or perceptual variations that their classification as non-iridescent is relatively unambiguous. However, it is noticed that values exceeding these criteria do not necessarily indicate iridescence, because the available literature does not provide sufficient evidence to establish corresponding lower thresholds for iridescent behavior. Instead, such cases should be evaluated considering their complete angular optical response and measurement conditions.

### 3.4. Spatial Resolution and Pixel Limit

Besides spectral, colorimetric, and angular characteristics, the spatial resolution of structural colors is another critical performance metric, particularly for applications including ultrahigh-resolution displays, color printing, anti-counterfeiting, and optical information storage. The spatial resolution is commonly described by the pixel density, expressed as dots per inch (DPI), and the optical independence between adjacent pixels, which is characterized by pixel crosstalk. Unlike conventional pigment-based coloration, however, the achievable spatial resolution of structural colors is fundamentally constrained by the underlying optical mechanism. Different structural color platforms rely on distinct resonance or interference processes, resulting in markedly different limits in pixel miniaturization and achievable DPI.

DPI is the most widely adopted metric for describing the spatial resolution of structural color devices. It represents the number of individually addressable pixels that can be accommodated within one inch, expressed as Equation (25)

(25) $$DPI=\frac{2.54\times 10^{4}}{p}$$

where *p* is the pixel pitch (μm). Higher DPI values correspond to smaller pixels and finer image details, making this parameter particularly important for applications requiring ultrahigh-resolution color reproduction. However, the maximum achievable DPI is not determined solely by fabrication resolution but also by the optical mechanism responsible for structural coloration. Structural colors generated through different optical mechanisms exhibit markedly different spatial resolution limits. Although DPI is commonly used to describe the spatial resolution of structural color devices, the limiting factors differ substantially among various coloration mechanisms. For ink-based structural colors, the achievable resolution is generally far below the optical limit because the minimum pixel size is primarily constrained by fabrication processes, such as droplet size, particle dimensions, or printing accuracy [208,209]. In contrast, interference-based structural colors, including multilayer thin films and photonic crystals, can be fabricated with submicrometer pixel dimensions. However, further pixel miniaturization is often accompanied by reduced reflectance efficiency and degraded color saturation because the optical resonance cannot be fully established within an excessively small pixel. Consequently, the practical pixel size is frequently determined by the trade-off between spatial resolution and optical performance rather than fabrication capability alone [210,211]. Localized resonance-based structural colors, including LSPR, Mie resonances, and dielectric metasurfaces, exhibit substantially greater flexibility in pixel miniaturization because each resonant nanostructure or meta-atom can function as an independent color-generating element. As a result, these platforms have demonstrated ultrahigh pixel densities approaching 10^5^–10^6^ DPI [212,213]. At these dimensions, the dominant limitations are no longer associated with color generation itself but arise from physical effects such as near-field coupling between neighboring resonators, collective resonance, quantum nonlocality, and finite-size effects. Therefore, the ultimate spatial resolution of structural colors is governed by different mechanisms depending on the coloration platform, highlighting the importance of considering both fabrication capability and the intrinsic physical limits of the optical resonance. A representative comparison of different structural color platforms, including their reported pixel size, achievable DPI, and the dominant factors limiting further pixel miniaturization, is summarized in Table 4.

The discussion above demonstrates that the achievable spatial resolution of structural colors is determined not only by the pixel size but also by the underlying optical mechanism and the physical limitations associated with different coloration platforms. While DPI and pixel crosstalk characterize the optical independence between neighboring pixels, they do not directly reflect the structural quality within each individual pixel. In practice, structural colors exhibiting identical pixel densities may still display significantly different optical performances due to variations in structural uniformity, fabrication defects, and long-range ordering. These structural imperfections can modify the resonance conditions, increase scattering losses, broaden the spectral response, and ultimately degrade color purity and reproducibility.

Therefore, in addition to evaluating spatial resolution, quantitative characterization of the structural order and defect distribution is essential for understanding the relationship between nanostructure quality and optical performance. The following section introduces the principal methods used to quantify structural ordering and fabrication defects in structural color systems.

### 3.5. Quantifying Structural Order and Defects

The optical performance of structural colors is highly dependent on the degree of order within the underlying micro- and nanostructures. Well-ordered periodic structures can precisely control the propagation, reflection, and interference of light, resulting in vivid colors with high brightness, high saturation, and excellent reproducibility. In contrast, structural disorder and defects can significantly alter the optical response of the material. Therefore, quantifying structural order and defect density is essential for understanding and optimizing structural color systems. Defects may arise during fabrication and include variations in pore size, lattice spacing, structural distortion, vacancies, dislocations, and irregular particle arrangements. These imperfections disrupt the structural periodicity and reduce the coherence of light scattering and interference. As a result, defects often broaden the reflection spectrum, reduce color purity and brightness, and weaken angle-dependent optical effects. In severe cases, excessive disorder may even suppress the formation of distinct structural colors. Consequently, a high degree of structural order is generally required to achieve stable and well-defined optical properties. Quantitative characterization of structural order and defect density not only provides insight into the relationship between morphology and optical performance but also serves as an important tool for optimizing fabrication processes and improving the performance of structural color devices. The following section introduces the principal methods used to quantitatively characterize structural order and defect density in structural color systems.

#### 3.5.1. Orientation Order Parameter: For Assembled Colloid

The orientation order parameter is an important quantitative metric for evaluating the degree of structural order in self-assembled colloidal systems. In recent years, colloidal self-assembly has become one of the most widely used approaches for fabricating photonic structures and structural color materials because of its simplicity, scalability, and ability to produce highly ordered periodic structures [214]. However, the optical performance of these materials is strongly influenced by the degree of order within the assembled colloidal array. Consequently, reliable methods for quantifying structural order are essential, and the orientation order parameter has become an important tool for this purpose. The orientation order parameter describes the degree of orientational alignment among neighboring particles or structural domains and is defined by Equation (26) [215,216].

(26) $$S=\frac{3{cos}^{2}\theta -1}{2}$$

Its value is typically normalized between 0 and 1. An orientation order parameter close to 0 indicates a highly disordered structure in which particles are randomly distributed without a preferred orientation [215,216]. In contrast, a value approaching 1 represents a highly ordered structure with long-range periodicity and consistent orientational alignment throughout the nanostructure. Therefore, the orientation order parameter provides a direct and intuitive measure for evaluating the quality of colloidal self-assembly and comparing the degree of structural order among different samples. The importance of the orientation order parameter is particularly evident in structural color systems. Structural colors arise from the interaction of light with periodic micro- and nanostructures, and their optical properties are highly sensitive to structural order. When colloidal particles are assembled into a well-ordered lattice, constructive interference and coherent light scattering occur efficiently, producing vivid colors with high brightness and high color saturation. Conversely, structural disorder disrupts the periodic arrangement, resulting in broadened reflection spectra, reduced color purity, lower saturation, and degraded optical performance. Consequently, a strong correlation is often observed between the orientation order parameter and the optical quality of structural colors. Several studies have employed the orientation order parameter to investigate the relationship between structural order and the optical properties of colloidal PCs. For example, it has been used to evaluate structural colors fabricated from self-assembled PS spheres [217]. By quantitatively correlating the degree of colloidal ordering with optical characteristics, the orientation order parameter provides a valuable framework for understanding how structural defects and assembly quality influence the optical performance of structural color systems. Therefore, the orientation order parameter has become a common metric for quantitatively correlating structural order with the optical performance of structural color systems.

#### 3.5.2. 2D FFT Analysis

**Figure 16:**
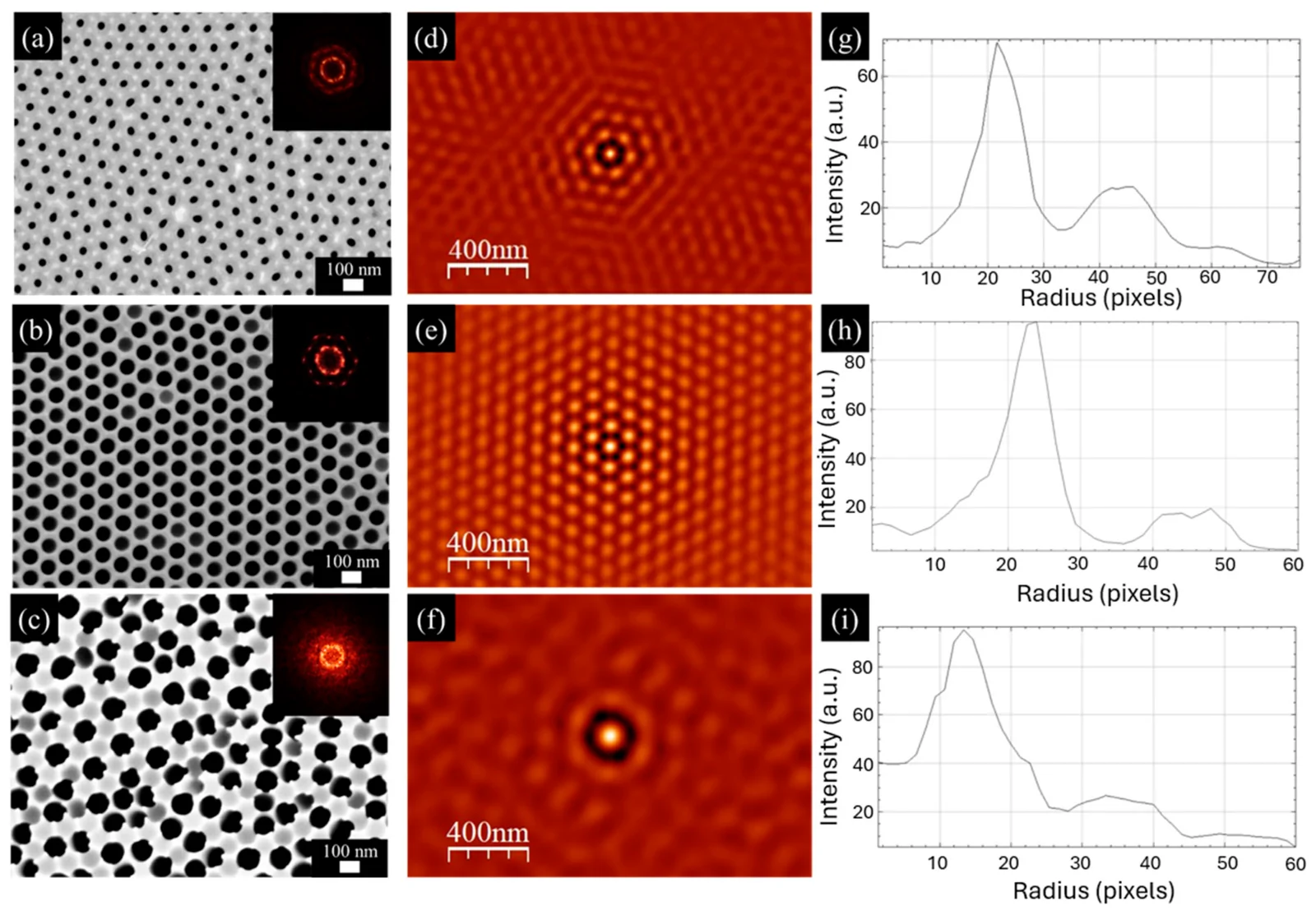
The (**a**–**c**) SEM micrographs and (**d**–**f**) pore-regularity analysis of two-step AAO from high-purity Al with second-step voltages at (**a**,**d**) 90 V (**b**,**e**) 100 V (**c**,**f**) 110 V, respectively [222]. (**g**–**i**) The radial profile converted from (**d**–**f**), respectively.

Two-dimensional fast Fourier transform (2D FFT) analysis is a powerful image-processing technique widely used to evaluate the structural regularity of nanoparticles, nanospheres, composite films, and nanostructured templates [218,219,220,221]. By transforming information obtained from SEM micrographs into the frequency domain, 2D FFT analysis enables the quantitative evaluation of structural order, symmetry, and characteristic periodic features that are often difficult to identify by direct visual inspection. Consequently, it has become an important tool for characterizing the quality of ordered nanostructures fabricated by self-assembly, anodization, lithography, and other nanofabrication techniques [222,223,224,225,226,227]. It is worth noting that the results and accuracy of 2D FFT analysis are influenced by analysis conditions such as the preprocessing procedure, field of view (FOV), and image resolution. Therefore, it is essential to compare the degree of structural ordering under consistent analysis conditions. In a highly ordered periodic structure, the 2D FFT pattern generally exhibits distinct and well-defined spots or narrow rings corresponding to characteristic spatial frequencies. As structural disorder increases, these features become broadened and diffuse because variations in lattice spacing, orientation, and defect distribution introduce a wider range of spatial frequencies. Quantitative information can be further extracted from the radial intensity profile of the 2D FFT pattern. The position of the principal radial peak represents the characteristic spatial periodicity of the structure, whereas its intensity and width reflect the degree of structural regularity. A highly ordered structure generally produces a strong and narrow FFT peak, while increasing structural disorder broadens the peak and increases its FWHM. For example, Chung et al. [222] developed a two-step mild–hard anodization process for fabricating anodic aluminum oxide (AAO) and used 2D FFT analysis to evaluate its structural regularity, as shown in Figure 16. Figure 16a–c show SEM micrographs of AAO surface morphology with 1st anodization voltage of 40/−2 V, and 2nd anodization at (a) 90/−4 V, (b) 100/−4 V, and (c) 110/−4 V, respectively. The highly ordered nanostructure shown in Figure 16b produces a clear and highly symmetric FFT pattern in Figure 16e, whereas the less ordered structure in Figure 16c produces a more diffuse FFT pattern in Figure 16f. Figure 16g–i show the corresponding radially averaged FFT intensity profiles. The less ordered structures exhibit broader radial peaks and larger FWHM values. Therefore, when the image resolution, FOV, preprocessing procedure, and FFT settings are maintained consistently, the FWHM of the principal FFT peak can serve as a quantitative parameter for comparing structural regularity.

Because the absolute FFT peak intensity and FWHM are affected by image dimensions, the number and density of structural elements, and the analyzed area, several studies have introduced normalized FFT-based parameters to enable more objective comparisons. Stępniowski et al. developed an FFT-derived regularity ratio (RR) for quantitatively evaluating nanopore arrangement in AAO and demonstrated that the effects of pore number, analyzed area, and porosity must be considered when comparing different images [223]. A representative regularity ratio used for periodic nanopore and nanotube arrays is expressed as Equation (27) [224].

(27) $$RR=\frac{I_{max}}{W_{1/2}D_{ave}}$$

where $I_{max}$ is the maximum intensity of the FFT peak, $W_{1/2}$ is its FWHM, $D_{ave}$ is the average spacing between neighboring structural elements. A larger RR corresponds to a stronger and narrower characteristic spatial-frequency peak and therefore indicates a higher degree of structural regularity [224]. The 2D FFT pattern is commonly used as indicators of structural order and defect density [222]. For structural color materials, the optical response strongly depends on the periodic arrangement of micro- and nanostructures. Because structural colors originate from diffraction, interference, or PC effects, even small variations in structural order can significantly influence color saturation, brightness, and spectral characteristics. Consequently, 2D FFT analysis has been widely used to investigate the relationship between structural order and optical performance.

In this chapter, the quantitative evaluation of structural colors is systematically reviewed. The quantitative evaluation of structural colors requires complementary metrics that characterize their optical, perceptual, and spatial performance from different perspectives. Spectral analysis provides direct information on the underlying optical response, including resonance wavelength, intensity, spectral selectivity, and sensing characteristics, thereby revealing the physical mechanisms responsible for color generation. Colorimetric analysis further transforms spectral information into standardized perceptual coordinates, enabling objective evaluation of color appearance, color gamut, and perceptual color differences through the CIE 1931 and CIE 1976 color spaces. Finally, spatial resolution metrics, including DPI, pixel crosstalk, and regularity, quantify the practical capability of structural color platforms to reproduce high-resolution images while maintaining optical independence between neighboring pixels. It should be emphasized that no single metric is sufficient to comprehensively evaluate structural color performance. For example, a narrow spectral linewidth does not necessarily guarantee high color saturation, an expanded color gamut does not imply excellent color fidelity, and an ultrahigh DPI may be accompanied by increased optical crosstalk or reduced chromatic performance. Therefore, meaningful comparison of different structural color technologies requires the combined consideration of multiple quantitative metrics rather than reliance on a single performance parameter.

Collectively, these quantification methods establish a unified framework for objectively evaluating structural colors across different optical mechanisms and application scenarios. Such a framework not only enables fair comparison among existing structural color platforms but also provides practical design guidelines for optimizing structural colors toward high color purity, wide color gamut, ultrahigh spatial resolution, and application-specific performance.

## 4. Applications of Structural Color

The unique optical properties of structural colors have enabled their application in a wide range of scientific and technological fields beyond decorative coloration. Unlike conventional pigments, structural colors can be engineered through precise control of nanostructure geometry and optical resonance, providing additional functionalities such as environmental responsiveness, tunable spectral characteristics, ultrahigh spatial resolution, and enhanced durability. Consequently, structural colors have attracted considerable attention for applications including functional coatings, chemical and biological sensing, optical spectroscopy, anti-counterfeiting, and information security. The performance requirements of these applications vary substantially and are closely associated with the quantitative metrics discussed in the previous section. For example, high color purity and environmental stability are essential for protective coatings, wavelength shift sensitivity is the primary figure of merit for optical sensing, while color gamut, spatial resolution, and structural complexity are critical for security labels and optical encryption. Therefore, understanding the relationship between structural color mechanisms and application-specific performance is essential for selecting appropriate design strategies and optimizing device functionality. The following sections highlight several representative applications of structural colors, focusing on surface protection and functional coatings, chemical and gas sensing, reflectometric interference spectroscopy (RIfS), and anti-counterfeiting and security labels. Rather than providing an exhaustive survey, these examples illustrate how different structural color mechanisms can be tailored to meet diverse functional requirements across a broad range of practical applications.

### 4.1. Surface Protection and Functional Coatings

Beyond producing vivid optical appearance, structural colors have increasingly been explored as multifunctional surface coatings that simultaneously provide optical, chemical, and thermal functionalities. Unlike conventional pigments, whose primary role is limited to coloration, structural colors are generated by precisely engineered micro- and nanostructures that can be further tailored to modify the physicochemical properties of the surface. By integrating structural coloration with functional surface engineering, these coatings can simultaneously achieve decorative esthetics, environmental durability, contamination resistance, and thermal management.

#### 4.1.1. Color Combined with Hydrophobicity and Self-Cleaning

Structural colors have attracted significant attention not only for their vibrant optical appearance but also for their potential in surface engineering. As structural color materials are increasingly applied to decorative coatings, consumer products, logos, and optical filters [228,229,230,231,232,233,234,235,236], their surface properties have become an important consideration alongside their optical performance. In particular, combining vivid structural colors with hydrophobic and self-cleaning properties has emerged as a promising direction for developing multifunctional materials [237,238,239]. The coloration of structural color systems originates from micro- and nanostructures that interact with light through interference, diffraction, or PC effects. Any environmental contamination or damage to these nanostructures can significantly degrade the optical quality of the structural colors. Therefore, developing hydrophobic structural color materials with anti-fouling and anti-adhesion properties can improve their stability, durability, and suitability for outdoor applications [237]. By carefully designing the surface morphology, structural color materials can exhibit hydrophobic behavior with low adhesion to contaminants. As a result, water droplets can easily roll off the surface, carrying away dust and other contaminants. This self-cleaning effect is particularly valuable for practical applications because it helps maintain both the visual appearance and optical performance of structural color coatings. To enhance hydrophobicity, structural color materials are typically fabricated from hydrophobic polymers or modified through surface nanostructuring or surface coatings. Polymer-based structural colors, such as those fabricated from PMMA, PS spheres, and poly(N-isopropylacrylamide), naturally exhibit hydrophobic behavior because of their intrinsic material properties [237,238,239], making them suitable for protective structural color coatings. In contrast, oxide and ceramic materials are generally hydrophilic. Therefore, additional surface nanostructuring or hydrophobic coatings are often required to improve their water repellency [240,241,242,243]. For example, Shukla et al. fabricated TiO_2_/Ti structural colors based on nanorod and thin-film configurations and quantitatively compared their wettability. The as-deposited TiO_2_ nanorod structures exhibited water contact angles of approximately 123–125°, substantially higher than the 79–94° obtained for the corresponding thin films. After UV irradiation, both structures could be transformed toward a superhydrophilic state, with the nanorod structures showing a faster wettability response. Moreover, annealing at 400 °C for 2 h produced a highly stable superhydrophilic surface, whose contact angle remained only approximately 18° even after 11 months of dark storage [240].

#### 4.1.2. Structural Color for Radiative Cooling

Recent advances in nanophotonics have demonstrated that structural colors can be strategically engineered for passive daytime radiative cooling applications [244,245]. For example, 3D AAO nanostructures have been employed as PC networks or Bragg reflectors and precisely designed to exhibit vivid structural colors across the visible spectrum without relying on light-absorbing pigments, thereby maintaining efficient passive cooling [246,247]. At the same time, these 3D porous ceramic structures exhibit exceptionally high thermal emissivity in the mid-infrared region, particularly within the atmospheric transparency window of 8–13 µm, where the emissivity can exceed 90% [248]. This high emissivity enables the nanostructured surfaces to efficiently radiate heat into outer space. Outdoor measurements under direct sunlight have demonstrated that these colorful radiative coolers can achieve temperature reductions of more than 10 °C compared with reference materials such as bare metals or aluminum foils [249]. This cooling performance is primarily attributed to the low thermal conductivity of oxide- and nitride-based materials, including SiO_2_, Si_3_N_4_, HfO_2_, or Al_2_O_3_ [244], which effectively suppress heat transfer from the surrounding environment. In addition, the thickness of the PC layer plays a critical role in determining the overall cooling performance. Studies have shown that increasing the thickness of the nanostructured alumina layer simultaneously enhances solar reflectance and selective mid-infrared emissivity. Consequently, thicker PC layers provide more effective passive radiative cooling [247]. This design strategy bridges nanoscale photonic engineering with macroscopic thermal management, providing a promising and esthetically versatile route toward sustainable, energy-free cooling technologies for buildings and vehicles.

### 4.2. Sensing

The optical response of structural colors is inherently sensitive to variations in the surrounding environment, making them attractive platforms for label-free optical sensing. External stimuli, such as changes in refractive index, chemical composition, gas adsorption, or surface binding events, can perturb the optical resonance and produce measurable variations in wavelength, reflectance intensity, or perceived color. Because these optical signals can be monitored without fluorescent or radioactive labels, structural color-based sensors offer advantages including real-time detection, simple optical readout, high stability, and low-cost implementation. Depending on the sensing mechanism and optical interrogation method, structural colors have been employed in a variety of sensing platforms. The following sections focus on three representative approaches: chemical and gas sensing, which relies on resonance shifts induced by analyte interactions; reflectometric interference spectroscopy (RIfS), which monitors interference spectral changes resulting from variations in optical thickness; and laser wavelength coupling and resonance-enhanced sensing, where coupling between an external laser source and structural resonances significantly improves detection sensitivity and spectral resolution.

#### 4.2.1. Chemical & Gas Sensing

**Figure 17:**
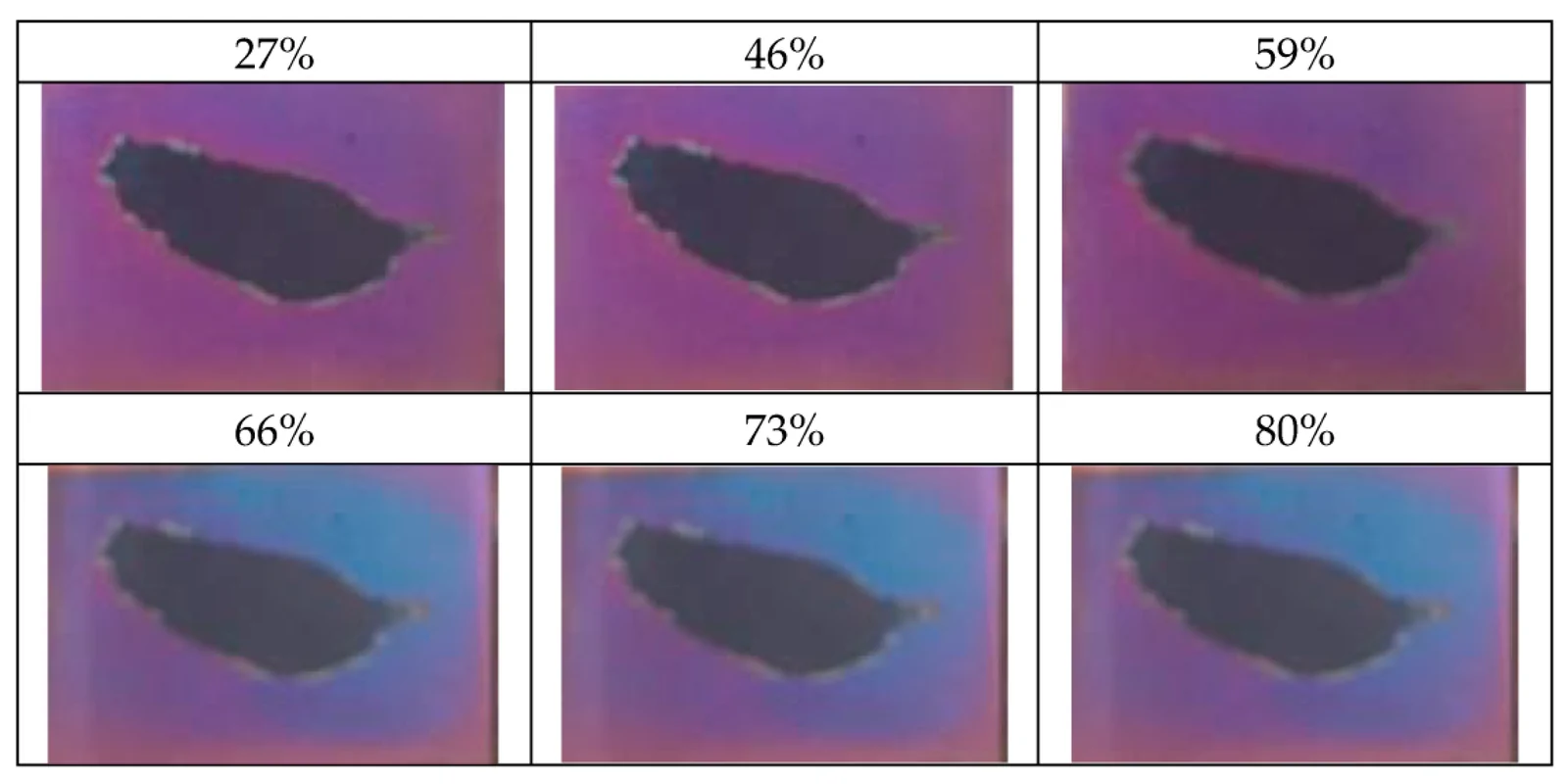
The optical AAO humidity sensor at relative humidity of 27% to 80% [256].

Structural color materials have attracted considerable attention as a novel sensing platform for chemical and gas detection [31]. Owing to their unique optical properties, structural colors can serve as highly sensitive indicators of environmental changes and have been successfully applied to humidity, gas, and ion sensing [248,249,250,251,252]. Compared with conventional sensing technologies that rely on electrical signal transduction [253,254,255,256,257,258], structural color sensors generate direct optical responses, enabling the detection of target analytes through observable color changes. This visual sensing capability is particularly attractive for portable, low-cost, and on-site monitoring applications, where rapid and intuitive interpretation of sensing results is desirable. The sensing mechanism of structural color-based humidity, gas, and ion sensors is primarily associated with changes in the effective refractive index. In thin-film, porous, or periodic nanostructured systems, air occupies part of the internal volume and contributes to the overall optical response. When target analytes, such as water vapor, gas molecules, or dissolved ions, enter the nanostructure, they partially replace the air within the pores. Because the refractive index of these analytes is generally higher than that of air, the effective refractive index of the structure increases. Consequently, the reflection peak usually exhibits a redshift toward longer wavelengths according to thin-film interference or Bragg’s law. This spectral shift is often accompanied by a visible color change, and its magnitude can be quantitatively correlated with the concentration of the target analyte. For example, a humidity nanosensor based on nanoporous alumina and the thin-film interference mechanism exhibits a clear redshift in its reflected color with increasing humidity, as shown in Figure 17 [256]. These characteristics demonstrate that structural color sensors provide a simple, intuitive, and highly visible alternative to conventional electrical sensing technologies for chemical and gas detection.

#### 4.2.2. Reflectometric Interference Spectroscopy (RIfS)

RIfS is a label-free optical sensing technique that exploits interference fringes generated by multiple reflections within thin films or porous nanostructures [41,259,260]. Unlike conventional structural color sensors that primarily monitor shifts in a single resonance wavelength, RIfS analyzes the entire interference spectrum, allowing subtle variations in optical thickness to be accurately determined. Changes in the refractive index or physical thickness of the sensing layer, induced by molecular adsorption, chemical reactions, or environmental stimuli, alter the optical path length and consequently shift the interference fringes. Because the sensing signal originates directly from interference rather than fluorescence or plasmonic enhancement, RIfS offers excellent long-term stability, simple optical instrumentation, and real-time, label-free detection. Owing to its high sensitivity to refractive index variations, RIfS has been widely employed for chemical sensing, biomolecular interaction analysis, environmental monitoring, and porous material characterization, making it one of the most representative interference-based sensing techniques in structural color research.

**Figure 18:**
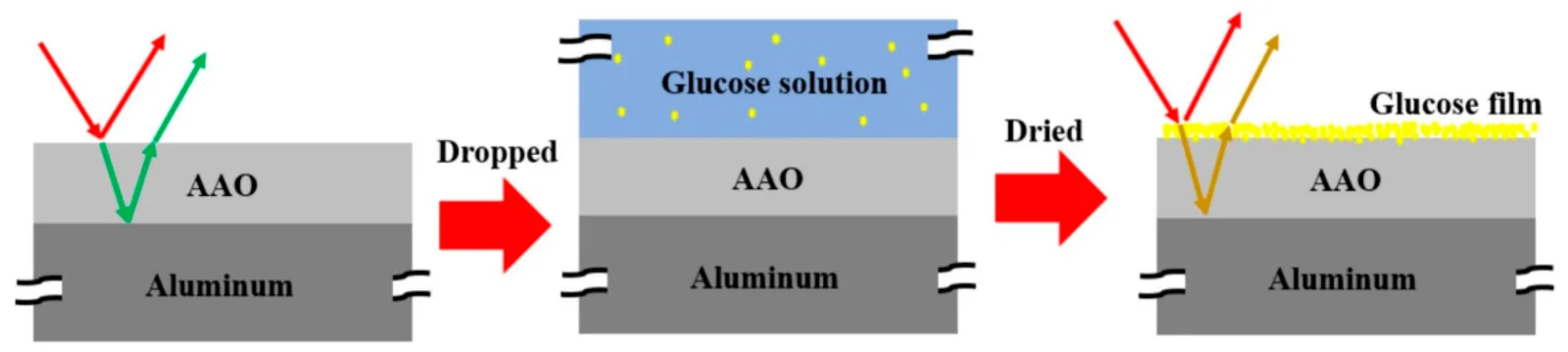
The schematic of glucose detection using one-step AAO based on RIfS. The arrows represent the incident/reflected light.

For example, Yu et al. proposed a sensitive optical sensing platform for label-free glucose detection using a simple one-step anodic aluminum oxide (AAO) substrate combined with RIfS, as shown in Figure 18. Experimental results showed that the AAO thin film prepared by short-time anodization possessed a lower interference order due to its thinner thickness. Compared to the traditional time-consuming long-term fabrication process, this film generated a larger spectral redshift under the same analyte concentration. Because the thinner AAO film had a lower interference order, a given change in optical thickness produced a larger spectral shift than that of thicker AAO films prepared by conventional long-term anodization. Consequently, this significantly enhanced the sensing sensitivity and lowered the detection limit. Furthermore, the pore structure after pore widening was also beneficial for the uniform adhesion of the glucose layer. In terms of quantitative data performance, the sensor exhibited an excellent linear response within the glucose concentration range of 400 to 800 ppm. Its linear correlation coefficient R^2^ reached 0.9957, and the sensing sensitivity was 0.033 nm/ppm, which equals 33 nm/(mg/mL) [41].

Additionally, RIfS can be combined with appropriate surface modification strategies to improve sensing sensitivity and selectivity. For example, Law et al. developed an optical sensing platform by integrating a perfluorosilanized nanoporous AAO interferometer with RIfS for the real-time, label-free detection of the formation of perfluorooctanoic acid (PFOA) self-assembled monolayers. During real-time PFOA detection, PFOA molecules bind to the perfluorosilane chains on the substrate surface through strong non-covalent fluorine-fluorine (F-F) interactions. This binding process changes the effective optical thickness, resulting in a measurable shift in the interference spectrum. The sensor exhibited a sensitivity of 4.9 ± 0.21 nm/(µg/mL), a limit of detection (LOD) of 0.53 ± 0.080 µg/mL, and a coefficient of determination R^2^ of 0.97 [260]. These examples demonstrate that RIfS-based structural color sensors can be quantitatively benchmarked using sensitivity, LOD, linear detection range, and linearity. However, direct comparison of sensitivity values requires consideration of the analyte, concentration unit, surface functionalization, and optical interrogation method.

#### 4.2.3. Spectral Matching and Resonance-Enhanced Sensing

In addition to monitoring spectral shifts induced by environmental changes, structural colors can also serve as resonant optical platforms for enhancing light–matter interactions through wavelength matching with an external laser source. Because many structural color systems exhibit narrow optical resonances with high spectral selectivity, the excitation efficiency strongly depends on the spectral overlap between the incident laser wavelength and the structural resonance. When the excitation wavelength coincides with the resonance, the local electromagnetic field can be significantly enhanced, leading to stronger optical signals and improved sensing performance.

**Figure 19:**
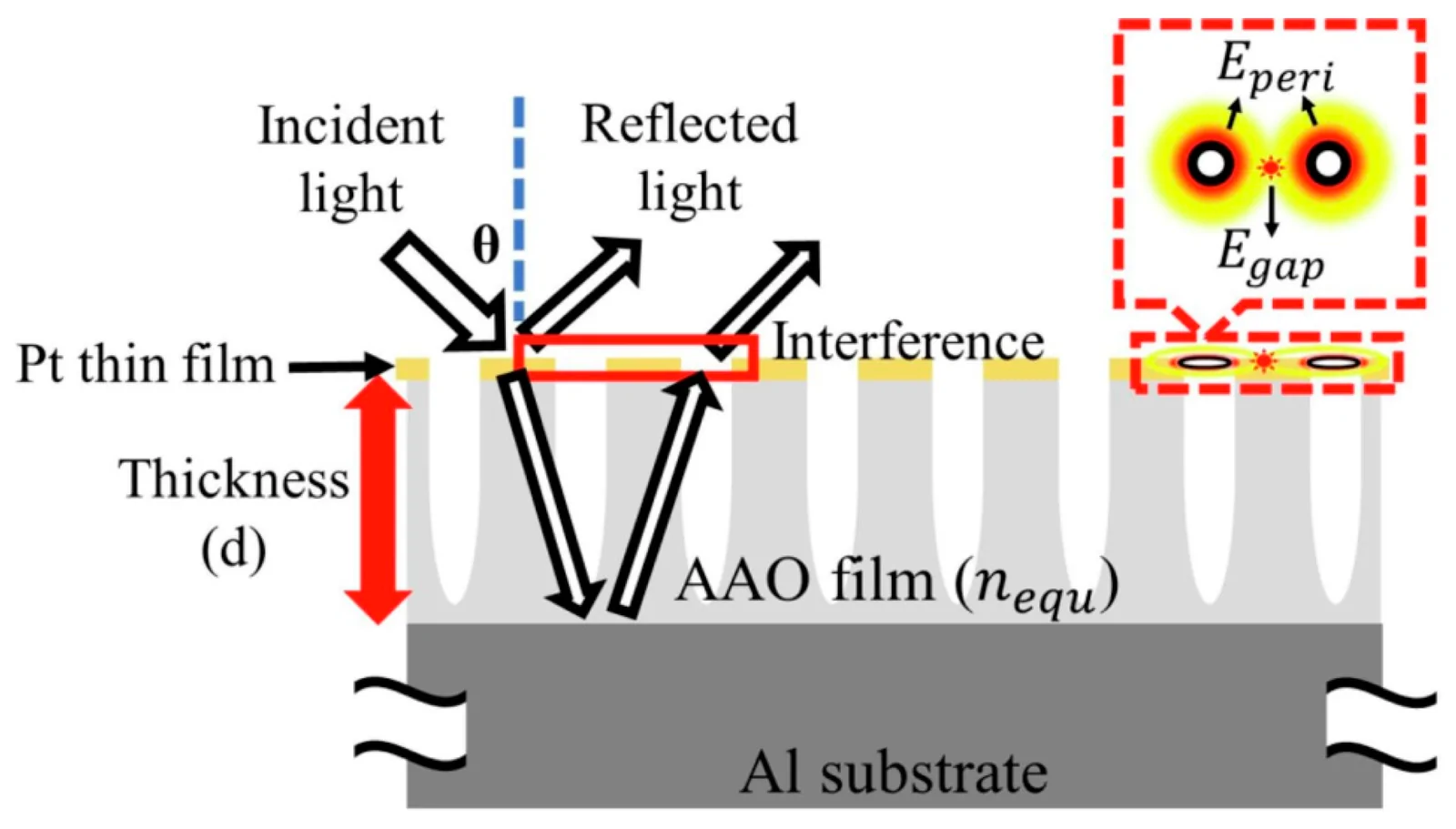
The schematic diagram of the dual-effect color and SERS mechanism [40]. The arrows represent the incident/reflected light.

This resonance-assisted excitation strategy has been widely applied to various optical sensing techniques, including Raman spectroscopy, SERS, fluorescence enhancement, and nonlinear optical sensing. By carefully designing the structural color to match the excitation wavelength, both the excitation efficiency and signal collection efficiency can be simultaneously improved. Consequently, resonance matching between the excitation laser and the structural color has become an effective approach for improving detection sensitivity, lowering the limit of detection, and enhancing the signal-to-noise ratio in resonance-based optical sensing platforms. For example, Yu et al. proposed an Al/AAO/Pt SERS substrate, as shown in Figure 19. The substrate utilized the interference between light reflected from the air-AAO/Pt interface and the AAO–Al interface to generate tunable structural colors through thin-film interference. By varying the anodization time, the thickness of the AAO layer was precisely controlled to match the excitation laser wavelength. This resonance matching reduced the reflectivity of the excitation laser, thereby increasing light coupling into the substrate. AAO thicknesses of 161, 179, 191, and 208 nm were obtained after anodization for 135, 160, 185, and 210 s, respectively. These substrates exhibited olive green, dark yellow, orange, and red structural colors. Under 532 nm laser excitation, their corresponding reflectivities were 72%, 56%, 48%, and 32%, respectively. Among them, the red substrate, with the lowest reflectivity of 32%, exhibited the strongest laser coupling and achieved approximately 6.8-fold higher SERS performance than the substrate with the highest reflectivity [40].

### 4.3. Anti-Counterfeiting and Security Labels

**Figure 20:**
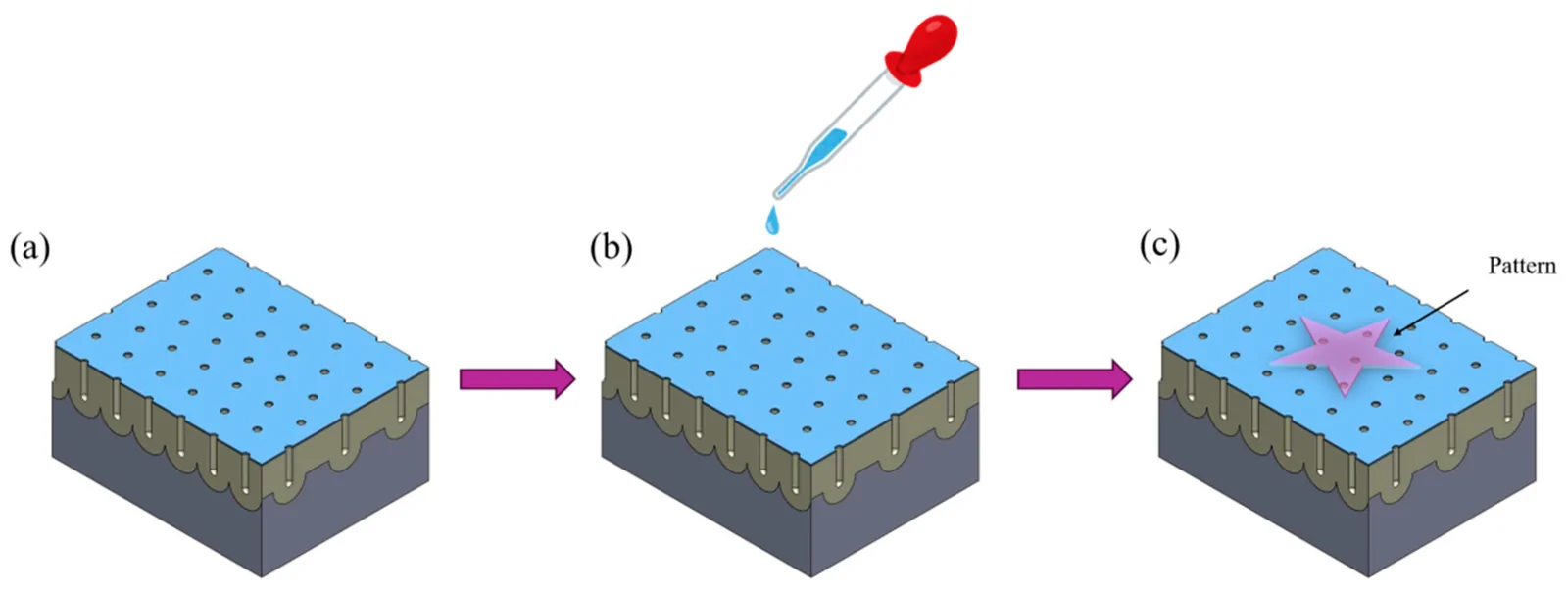
Schematic diagram of an anti-counterfeiting application using structural color: (**a**) original structural color device, (**b**) exposure to a liquid medium, and (**c**) recognition of the invisible pattern.

Structural colors have emerged as an advanced platform for anti-counterfeiting technologies owing to their unique optical properties and the complexity of their micro- and nanostructures [154,261,262,263,264]. Unlike conventional printing or pigment-based coloration, structural colors are generated through the physical interaction of light with periodic nanostructures, making them extremely difficult to replicate using traditional manufacturing methods. Among the various types of structural colors, thin-film interference- and PC-based structural colors have been widely employed for invisible patterns and security labels. The operating principle of these anti-counterfeiting systems is based on changes in the effective refractive index of the nanostructure [262,263], which is similar to the sensing mechanism discussed in Section 4.2. However, the successful visualization of hidden patterns relies on regions with different structural parameters that produce distinct wavelength shifts. These structural variations are typically fabricated using lithographic techniques. Initially, air occupies the pores or intermediate layers of the nanostructure, producing a specific reflection peak. When another medium infiltrates the nanostructure and replaces the trapped air, the effective refractive index increases, resulting in a redshift of the reflection peak [262,263,265], as illustrated in Figure 20. Figure 20 schematically illustrates (a) a structural color device, (b) infiltration by a liquid medium, and (c) patterned regions exhibiting different structural colors. Water is the most commonly used infiltration medium [262], although other materials, such as ethanol [265] or PDMS [262], have also been employed. As the reflection peak shifts toward longer wavelengths, the hidden patterns become visible. After the infiltrating medium is removed from the nanostructure, the effective refractive index decreases, causing the reflection peak to blueshift back to its original wavelength. Furthermore, the optical response can be precisely controlled by tailoring the porosity and geometry of the nanostructure. Regions with different pore sizes or porosities exhibit different wavelength shifts upon infiltration, allowing selected areas to reveal distinct colors or hidden images. This design strategy enables the fabrication of security labels with multiple optical states that are difficult to counterfeit. In addition to their dynamic optical response, structural colors inherently exhibit high brightness, high color saturation, and angle-dependent optical effects, making them readily distinguishable from conventional pigment-based colors. This strategy can also be combined with digital spectral analysis [261] or hybrid authentication based on structural color and photoluminescence [264] to further enhance anti-counterfeiting performance. Consequently, structural colors have shown great potential for invisible images, anti-counterfeiting patterns, security labels, and information protection.

The representative applications discussed above demonstrate that structural colors have evolved far beyond their original role as decorative materials. By exploiting precisely engineered optical resonances and nanostructured architectures, structural colors can simultaneously provide multiple functionalities, including environmental protection, thermal management, label-free optical sensing, resonance-enhanced spectroscopy, and information security. Although these applications differ considerably in their operating principles and target functions, they all originate from the ability to precisely manipulate light through micro- and nanostructures. More importantly, the successful implementation of these applications is closely related to the quantitative metrics discussed in the previous section. Spectral characteristics determine resonance behavior and sensing performance, colorimetric analysis enables objective evaluation of visual appearance, while spatial resolution and structural quality directly influence device reliability and practical applicability. Therefore, quantitative characterization not only provides standardized evaluation criteria but also establishes the fundamental relationship between structural design, optical performance, and functional applications. Collectively, these representative examples highlight the versatility of structural colors and demonstrate their significant potential for future multifunctional optical devices.

## 5. Conclusions and Outlook

Structural colors have developed from an optical phenomenon inspired by nature into a versatile nanophotonic platform for manipulating color through engineered light–matter interactions. In this review, structural colors were classified according to their macroscopic angular optical responses into iridescent and non-iridescent systems, while recognizing that the relationship between optical mechanism and angular response is not necessarily one-to-one. Thin films, ordered PCs, and diffraction gratings represent major routes to iridescent coloration, whereas independent resonators, quasi-amorphous structures, and engineered metasurfaces provide different strategies for reducing angular dependence. Beyond structural classification, this review emphasizes that meaningful comparison of structural colors requires quantitative characterization of their spectral, colorimetric, angular, spatial, and structural properties. The applications discussed herein further demonstrate that structural colors have evolved beyond decorative coloration toward multifunctional coatings, sensing, radiative cooling, spectroscopy, anti-counterfeiting, and information security.

Despite substantial progress, several fundamental and technological limitations still restrict the transition of structural colors from laboratory demonstrations to large-scale applications. One important challenge is the inherent trade-off among optical performance parameters. High color saturation, brightness, wide color gamut, angular stability, and ultrahigh spatial resolution cannot always be optimized simultaneously. For example, introducing structural disorder can reduce angular dependence but may broaden the reflection spectrum and decrease color saturation. Similarly, reducing pixel dimensions can increase spatial resolution but may weaken the optical resonance or increase interactions between neighboring color-generating elements. Structural color design should therefore move away from optimizing a single parameter and toward balancing multiple application-specific figures of merit.

A second major limitation concerns color visibility and spectral purity, particularly in particle-based and quasi-amorphous systems. Multiple scattering can generate a broadband optical background that competes with the desired wavelength-selective reflection, producing pale or whitish appearances. This problem is particularly important for highly saturated long-wavelength colors, especially red. Absorption management has emerged as an effective strategy for overcoming this limitation. Broadband absorbers such as carbon black, PDA, and graphene-based materials can suppress undesired multiple-scattering contributions, while absorbing core–shell or hybrid structures can selectively attenuate unwanted spectral components. However, excessive absorption can simultaneously reduce brightness. Future designs therefore need to control not only the amount of absorption but also its spectral distribution and spatial location within the photonic structure, thereby achieving an appropriate balance between background suppression, saturation, and brightness. The newly discussed absorption-management strategies illustrate this important direction toward practical high-visibility structural colors.

Fabrication scalability, structural uniformity, and reproducibility represent another major challenge. Top–down methods such as EBL provide precise control over nanoscale geometry and are particularly suitable for high-performance resonators and metasurfaces, but their cost and limited throughput remain major barriers to large-area production. Bottom–up self-assembly and printing methods provide substantially better scalability, but defects, particle-size variations, nonuniform ordering, and local structural fluctuations can lead to sample-to-sample variations in optical performance. Future manufacturing strategies will therefore need to combine the precision of top–down nanofabrication with the throughput and cost advantages of bottom–up processing, printing, replication, and self-assembly. The development of reliable large-area processes with controlled defect density will be particularly important for translating structural colors into commercial coatings, security labels, and other macroscale products.

The mechanical and environmental stability of structural colors also requires greater attention. Because their optical properties are determined directly by micro- and nanoscale geometry, contamination, abrasion, deformation, swelling, oxidation, or structural collapse can modify the optical response even when the constituent materials themselves remain chemically stable. This issue becomes particularly important for outdoor coatings, flexible devices, wearable systems, and sensors that must operate under repeated environmental exposure. Emerging multifunctional strategies that combine structural coloration with hydrophobicity, self-cleaning behavior, mechanical protection, or chemically resistant encapsulation provide promising routes to improved durability. Rather than treating optical performance and surface protection as separate design objectives, future structural color materials should increasingly integrate both functions within the same architecture.

Another unresolved issue is the lack of fully standardized methods for comparing structural color performance. Peak wavelength, peak intensity, FWHM, color coordinates, color difference, angular wavelength shift, spatial resolution, and structural-order parameters each describe different aspects of performance and are not universally applicable to every structural color mechanism. In addition, a universally accepted numerical boundary separating iridescent and non-iridescent structural colors has also not yet been established. Future studies should therefore report geometry, spectral conditions, image-analysis parameters, and relevant application-specific figures of merit more consistently. Establishing standardized characterization and reporting protocols will be essential for meaningful benchmarking among different structural-color technologies and for evaluating whether laboratory performance can be reproduced at the manufacturing scale.

Overall, the future of structural color materials is unlikely to be defined by a single optical mechanism or fabrication route. Instead, progress will depend on balancing color quality, angular response, structural precision, durability, multifunctionality, scalability, and cost according to the requirements of each application. By connecting color-generation mechanisms with quantitative evaluation methods and practical performance requirements, the framework presented in this review provides a basis for comparing different structural color platforms and for guiding the development of next-generation multifunctional and sustainable structural color designs.

## Figures and Tables

**Table 1:** A comparison of iridescent structural colors.

Structure	Mechanism of Forming Iridescence	Advantages	Limitations
Single Layer Thin Film	Interference, a blueshift with increasing viewing angle from the normal, governed by the shortening of the optical path difference.	Facile fabrication process and high tunability.	Color saturation is usually poor.
Multi-Layer Thin Film		1. MDM resonant cavities significantly enhance the saturation of colors. 2. Multilayer thin films effectively narrow the spectral peak width, achieving pure colors (e.g., pure red).	1. Single MDM structures struggle to eliminate stray/hybrid colors. 2. Involves optical absorption and complex phase shifts, increasing the difficulty of theoretical design and computation.
1D PCs		1. Compatible with various thin-film technologies and fabrication processes, offering a wide selection of materials. 2. Enables bidirectional structural color with completely different colors on the front and back sides.	1. Typically requires layer-by-layer stacking of multiple pairs, leading to a time-consuming overall fabrication process. 2. Precisely and uniformly controlling the thickness of each thin-film layer in large-area fabrication is a severe challenge.
2D PCs	Diffraction, a redshift with increasing viewing angle from the normal, governed by in-plane wavevector conservation.	Single-layer geometric structures can be realized through rapid and simple microsphere self-assembly techniques, making the fabrication process relatively fast.	The material selection and types of colloidal microspheres used for preparation are relatively limited; the resulting structural colors usually exhibit low saturation and poor visual contrast.
3D PCs-Opal	Multi-wave coherent interference driven by lattice-induced Bragg diffraction. A blueshift with increasing viewing angle from the normal, governed by the shortening of the optical path difference.	1. Typically fabricated by bottom–up colloidal self-assembly, featuring low fabrication costs and fast manufacturing speeds. 2. Microsphere colloids can be directly formulated into photonic crystal inks for writing or coating color display.	
3D PCs-Inverse Opal		1. Possesses good refractive index contrast, significantly enhancing reflection efficiency, saturation, and visual contrast. 2. Features a 3D interconnected porous network and high specific surface area, allowing easy diffusion of environmental molecules. This makes it sensitive to variations in the effective refractive index of the surrounding medium.	During the critical process of removing microsphere templates, the solid network often generates microcracks or even localized lattice collapse due to drastic volume shrinkage, internal stress concentration, or non-uniform surface tension.
3D PCs-Woodpile		Possesses the highest degree of structural regularity and geometric programmability.	Fabrication highly relies on advanced 3D micro/nano-fabrication technologies such as two-photon polymerization (TPP) and direct ink writing (DIW), resulting in extremely high equipment costs and tedious layer-by-layer stacking procedures.
Diffraction Gratings	Diffraction, a redshift with increasing viewing angle from the normal, governed by in-plane wavevector conservation.	1. Large-scale nanoimprint lithography (NIL) makes structured products like optical disks extremely low-cost, offering large-scale production capacity. 2. Introducing composite or superimposed square-wave phase grating structures can enhance saturation in the cyan bands.	Traditional single-line or single-layer square-wave designs suffer from limitations in color saturation and spectral modulation, making it particularly difficult to achieve high purity in the cyan and green bands.

**Table 2:** A comparison of non-iridescent structural colors.

Path	Structure	Mechanism	Advantages	Limitation
Independent Resonators	Metallic nanoparticles or subwavelength uncoupled arrays.	LSPR in metals.	1. Enormous extinction cross-section and vivid coloration. 2. Facile shape-dependent tuning via chemical synthesis.	1. Significant intrinsic visible light absorption loss. 2. Color decay caused by metal nanostructure oxidation.
	Dielectric microspheres or micropillars.	Mie resonance in dielectrics.	1. Low intrinsic visible absorption loss. 2. High compatibility with semiconductor processing.	1. Large imaginary part of silicon below 450 nm. 2. Optical isolation required to prevent aggregation-induced color decay.
1D quasi-amorphous structures	Vertical stacks of alternating refractive-index multilayers with random thickness fluctuations.	Interface interference of randomly thick layers smoothing the reflection band.	Color uniformity across a defect-free flat plane.	1. High production costs for stacking dozens of thin films. 2. Incompatibility with irregular curved surfaces due to flat geometry.
2D quasi-amorphous structures	Isotropic surface wrinkle structures.	Isotropic interference and scattering of specific light wavelengths.	1. High color contrast via single-plane confinement. 2. High strain sensitivity enabling advanced optical sensing applications.	High vulnerability to mechanical scratching, environmental fouling, or strain-induced color degradation.
3D quasi-amorphous structures	Short-range ordered and long-range disordered microsphere assemblies.	Coherent light scattering/interference from isotropic multi-particle distributions.	Good scalability and high commercial viability for large-area bottom–up processing.	1. Poor intrinsic color saturation. 2. Formidable challenges in generating pure long-wavelength colors, such as red.
Metasurface	Spatially engineered two-dimensional arrays of subwavelength nanostructures.	Abrupt interfacial phase discontinuities driven by geometric phase, propagation phase, or collective resonances.	1. Exceptionally precise near-field light manipulation. 2. Simultaneous control over amplitude, phase, polarization, and wavefronts.	High fabrication costs rendering it commercially unviable for passive large-area decorative coloration alone.

**Table 3:** Summary of methods, angular ranges, and quantitative metrics used to evaluate the angular stability of representative structural color systems.

Materials	Structures	Methods for Determining Angular Stability	Angular Stability Range	Quantitative Metric /Results	Ref.
SiO_2_ microspheres	3D quasi-amorphous structures	Spectra	0° to 40°	Non-quantitative	[154]
SiO_2_ microspheres	3D quasi-amorphous structures	Optical images	0°to 75°	Non-quantitative	[201]
Hollow Si	Metasurface	Spectra	0° to 60°	Non-quantitative	[202]
Latex particles	3D quasi-amorphous structures	Spectra	0° to 45°	Non-quantitative	[203]
Hydroxypropyl cellulose acrylate (HPCA) microspheres	1D quasi-amorphous structures	Spectra	15° to 45°	Non-quantitative	[204]
Cellulose nanocrystal	1D quasi-amorphous structures	Spectra	0° to 60°	(Δλ(θ)) < 10 nm	[138]
a-Si/SiO_2_	Multiple layers thin-films	Spectra	0° to 80°	($∆\lambda \left(\theta \right)/\lambda \left(\theta_{0}\right)$) about 1.8%.	[197]
Si/quartz	2D subwavelength grating	CIEDE2000	0° to 45°	$∆E_{00}^{*}(\theta )\;$<8	[205]
Ag/ SiO_x_/Ag	Multiple layers thin-films	Spectra and CIEDE2000	0° to 60°	$∆E_{00}^{*}(\theta )\;$ < 7.09 at 40° For Ag > 30 nm, (Δλ(θ)) < 5 nm up to 60°	[206]
ZnS/Ag/ZnS on SiO_2_	1D subwavelength grating	Spectra and CIEDE2000	0° to 60°	$∆E_{00}^{*}(\theta )\;$ < 6.99 at 60° and (Δλ(θ)) < 15 nm	[207]

**Table 4:** Comparison of representative structural color platforms in terms of pixel size, reported spatial resolution (DPI), and the dominant factors limiting further pixel miniaturization.

Structure	Mechanism	Pixel Size	Approximate DPI	Resolution-Limiting Factor	Ref.
Pigments or Dyes	Absorption by molecules	~25 μm	~1000	Size of each ink droplet	[208]
Photonic Ink	Interference or diffraction of photonic crystals	6.6 μm–1.2 mm	21–3848	Fabrication process (Ink)	[209]
ZnS/MgF_2_ 1D PCs	Interference	5–20 μm	1270–5080	Color manipulation concern	[210]
Ti/TiO_2_/Ti MDM structure	Interference	800 nm	3 × 10^5^	Fabrication Process (Laser Oxidation)	[211]
Borophene metasurfaces	LSPR	Tens of nm	~10^6^	Quantum non-local and fine-size effects	[212]
Si nanopillars array	Mie Resonances	338–800 nm	31,750–75,174	Collective Resonance Limit	[213]

## Data Availability

Data are presented in the coauthors’ research results, and the schematic drawing is available upon request.

## Supplementary Materials

The following are available online at https://www.mdpi.com/article/10.3390/nano16161031/s1, 3D model of the IO structure.
